# Are Non-animal Systemic Safety Assessments Protective? A Toolbox and Workflow

**DOI:** 10.1093/toxsci/kfac068

**Published:** 2022-07-13

**Authors:** Alistair M Middleton, Joe Reynolds, Sophie Cable, Maria Teresa Baltazar, Hequn Li, Samantha Bevan, Paul L Carmichael, Matthew Philip Dent, Sarah Hatherell, Jade Houghton, Predrag Kukic, Mark Liddell, Sophie Malcomber, Beate Nicol, Benjamin Park, Hiral Patel, Sharon Scott, Chris Sparham, Paul Walker, Andrew White

**Affiliations:** Unilever Safety and Environmental Assurance Centre, Bedfordshire MK44 1LQ, UK; Unilever Safety and Environmental Assurance Centre, Bedfordshire MK44 1LQ, UK; Unilever Safety and Environmental Assurance Centre, Bedfordshire MK44 1LQ, UK; Unilever Safety and Environmental Assurance Centre, Bedfordshire MK44 1LQ, UK; Unilever Safety and Environmental Assurance Centre, Bedfordshire MK44 1LQ, UK; Cyprotex Discovery Ltd, Cheshire SK10 4TG, UK; Unilever Safety and Environmental Assurance Centre, Bedfordshire MK44 1LQ, UK; Unilever Safety and Environmental Assurance Centre, Bedfordshire MK44 1LQ, UK; Unilever Safety and Environmental Assurance Centre, Bedfordshire MK44 1LQ, UK; Unilever Safety and Environmental Assurance Centre, Bedfordshire MK44 1LQ, UK; Unilever Safety and Environmental Assurance Centre, Bedfordshire MK44 1LQ, UK; Unilever Safety and Environmental Assurance Centre, Bedfordshire MK44 1LQ, UK; Unilever Safety and Environmental Assurance Centre, Bedfordshire MK44 1LQ, UK; Unilever Safety and Environmental Assurance Centre, Bedfordshire MK44 1LQ, UK; Cyprotex Discovery Ltd, Cheshire SK10 4TG, UK; Charles River Laboratories, Cambridgeshire, CB10 1XL, UK; Unilever Safety and Environmental Assurance Centre, Bedfordshire MK44 1LQ, UK; Unilever Safety and Environmental Assurance Centre, Bedfordshire MK44 1LQ, UK; Cyprotex Discovery Ltd, Cheshire SK10 4TG, UK; Unilever Safety and Environmental Assurance Centre, Bedfordshire MK44 1LQ, UK

**Keywords:** Bayesian modelling, new approach methodologies, point of departure, physiologically based pharmacokinetics, probabilistic risk assessment

## Abstract

An important question in toxicological risk assessment is whether non-animal new approach methodologies (NAMs) can be used to make safety decisions that are protective of human health, without being overly conservative. In this work, we propose a core NAM toolbox and workflow for conducting systemic safety assessments for adult consumers. We also present an approach for evaluating how protective and useful the toolbox and workflow are by benchmarking against historical safety decisions. The toolbox includes physiologically based kinetic (PBK) models to estimate systemic *C*_max_ levels in humans, and 3 bioactivity platforms, comprising high-throughput transcriptomics, a cell stress panel, and *in vitro* pharmacological profiling, from which points of departure are estimated. A Bayesian model was developed to quantify the uncertainty in the *C*_max_ estimates depending on how the PBK models were parameterized. The feasibility of the evaluation approach was tested using 24 exposure scenarios from 10 chemicals, some of which would be considered high risk from a consumer goods perspective (eg, drugs that are systemically bioactive) and some low risk (eg, existing food or cosmetic ingredients). Using novel protectiveness and utility metrics, it was shown that up to 69% (9/13) of the low risk scenarios could be identified as such using the toolbox, whilst being protective against all (5/5) the high-risk ones. The results demonstrated how robust safety decisions could be made without using animal data. This work will enable a full evaluation to assess how protective and useful the toolbox and workflow are across a broader range of chemical-exposure scenarios.

##  

The rapid development of new, non-animal approaches for conducting toxicological safety assessments has been driven by several factors. These include ethical considerations, regulatory action (animal test bans for certain types of ingredients), and the need to assure the safety of chemicals using efficient, cost-effective, and robust methods (Dent *et al.*, 2018, 2021; Thomas *et al.*, 2019). Non-animal approaches also have the potential to improve safety assessments by using more human-relevant tools through coverage of key biological pathways or targets. Next-generation risk assessment (NGRA) provides a way to integrate new approach methodology (NAM) data from various sources into the decision-making process, allowing for safety assessments to be conducted without the use of animal data. Recently, the International Cooperation for Cosmetics Regulation outlined 9 principles for the use of NGRA to make decisions on consumer safety for ingredients in cosmetics products (Dent *et al.*, 2018). In particular, the approach is (1) exposure-led, (2) hypothesis driven, (3) uses a tiered and iterative approach to make safety decisions, and (4) is designed to prevent harm. Although low tier approaches such as exposure-based waiving (Yang *et al.*, 2017) or history of safe use (Constable *et al.*, 2007; Neely *et al.*, 2011) will be sufficient to make a decision on safety for some chemical-exposure scenarios, when this is not possible, risk assessments can be completed at higher tiers using appropriate NAM-based tools and approaches. Several frameworks describing how NAMs can be integrated for safety decision making have also been developed over the last decade, most notably the SEURAT-1 tiered workflow for conducting *ab initio* risk assessments of systemic repeat-dose toxicity (Berggren *et al.*, 2017), and the next-generation blueprint of computational toxicology from the U.S. Environmental Protection Agency (EPA) (Thomas *et al.*, 2019).

To demonstrate the practical application of the frameworks and principles underpinning NGRA, detailed case studies have recently been published focusing on the *ab initio* risk assessment of specific ingredients under various exposure scenarios using NAMs (Baltazar *et al.*, 2020; OECD, 2021). A general concept throughout is that if the exposure level of a chemical in humans is far below the concentration needed for it to have any biological effect, then it is unlikely to trigger any toxicity. Estimates of systemic exposure are obtained using physiologically based kinetic (PBK) models (Bois *et al.*, 2017; Mumtaz *et al.*, 2012; Pearce *et al.*, 2017) and potential biological effects are assessed using points of departure (PODs) from *in vitro* assays (Farmahin *et al.*, 2017; Harrill *et al.*, 2019, 2021; Reynolds *et al.*, 2020). The approach is designed to be protective of human health rather than predictive of any specific toxicities (Dent *et al.*, 2018; Paul Friedman *et al.*, 2020). As such, the *in vitro* assays are selected based on whether they are able to detect very early biological perturbations, before the onset of any adverse effects. Examples of relevant assays include high-throughput transcriptomics (Harrill *et al.*, 2021), phenotypic profiling (Nyffeler *et al.*, 2020), assays for measuring cellular stress (Hatherell *et al.*, 2020), or profiling of specific biological targets, such as key receptors, enzymes, transporters, and ion channels (Bowes *et al.*, 2012). The PODs and exposure estimates can be combined into a single metric, the bioactivity exposure ratio (BER) (or margin of safety) (Baltazar *et al.*, 2020; Wetmore *et al.*, 2015). Overall, the approach is similar to traditional risk assessment, in that toxicologists do not expect animals to behave exactly as humans (Hartung, 2008; Van Norman, 2019), or to express the same adverse effects following administration of a chemical. However, no-observed-(adverse)-effect levels from toxicology studies in animals have been used as pragmatic PODs reflecting *in vivo* bioactivity for many years, which is then compared with predicted levels of consumer exposure to give a margin of safety for decision-making (SCCS, 2021c).

Although individual case studies have helped exemplify the overall NGRA approach, there is still a need to establish a standardized set of tools and workflows for obtaining an initial BER estimate when low-tier approaches are not sufficient, and determine whether these can be used reliably for a wide range of chemicals and exposure scenarios (Dent *et al.*, 2021). It is envisaged that such an approach could be used to decide, depending on the BER, whether a given chemical-exposure scenario is low risk, or whether to use higher tier approaches to refine the risk assessment further. Within this, there are various factors that will determine the overall protectiveness of such an approach, such as the “biological coverage” of the *in vitro* assays (do the assays cover enough biological effects to be protective?) (Paul Friedman *et al.*, 2020; Thomas *et al.*, 2019), or uncertainty in the accuracy of the PBK estimates (Moxon *et al.*, 2020; Paini *et al.*, 2017, 2021; Punt *et al.*, 2022; Wambaugh *et al.*, 2019), particularly when the models are parameterized using either *in silico* predictions or *in vitro* data. Furthermore, there are currently no guidelines for determining a BER threshold that represents a low safety risk. In traditional risk assessment, uncertainty factors are used that account for the intra- and interspecies differences in toxicodynamics and toxicokinetics. Typically for cosmetic applications, a margin of safety of 100 is considered acceptable to assure consumer safety and account for these variations (SCCS, 2021c). However, these have in general been established through historical precedent and experience (Renwick, 1993) that developed over many decades, which is not the case with NAMs, necessitating an alternative approach for defining suitable safety thresholds (Rusyn and Chiu, 2022).

We propose to address the above challenges in 2 steps. First, in this work, we present a core toolbox of NAMs (*in vitro* and computational) together with a workflow for how they should be used together to calculate the BER, based on recent systemic NGRA case studies (Baltazar *et al.*, 2020). We also present an approach for evaluating how protective and useful the combined workflow and toolbox are for conducting systemic safety assessment for a given chemical-exposure scenario. An important part of the approach is establishing a prototype decision model (eg, safety thresholds) for identifying low-risk exposure scenarios based on the BER. This is done in a data-driven manner, where the data from this initial (pilot) study will serve as a training set for a larger evaluation. The full evaluation of the toolbox and associated decision model will involve generating toolbox data (ie, corresponding to a test set) for a much larger set of compounds and exposure scenarios. Taking this 2-step approach can help to remove potential bias that can emerge due to post-rationalization of the data.

The evaluation approach is based on the idea of benchmarking BERs generated using the toolbox and workflow against historical safety decisions, the key principles of which are demonstrated in this work using data generated for 24 different exposure scenarios covering 10 chemicals. In summary, the objectives of this work are 3-fold:

1. Present a core toolbox of NAMs (*in vitro* and computational) together with a workflow on how they should be used together to provide an initial BER estimate for use in systemic toxicity safety assessments, obtained without the use of animal data.
2. Present a proof-of-concept study on how to evaluate the performance of the toolbox and workflow using benchmarks based on historical safety decisions.
3. Use this pilot study to establish a prototype decision model upon which to conduct the full evaluation.

## MATERIALS AND METHODS

### Overview of the Toolbox and Workflow

**Figure 1.**
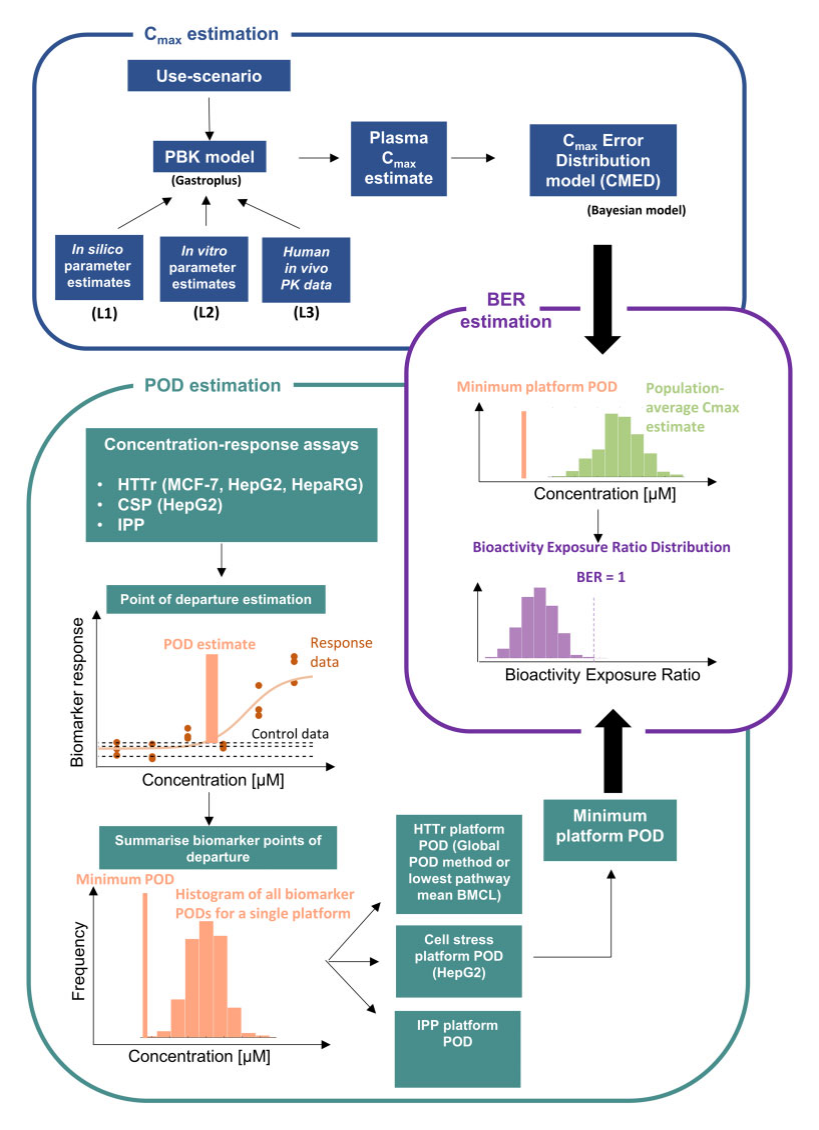
Schematic of the systemic safety toolbox and associated workflow, which comprises 3 modules: one to estimate the exposure using physiologically based kinetic (PBK) models, another to estimate the point of departure (POD) based on the cell stress panel (CSP), high throughput transcriptomics (HTTr), and *in vitro* pharmacological profiling (IPP) bioactivity data. The workflow involves combining the outputs from these 2 modules into the third module to estimate the bioactivity exposure ratio (BER).

Following previous NGRA case studies for systemic exposure (Baltazar *et al.*, 2020; OECD, 2021), the workflow is divided into 3 distinct modules (Figure 1): one for estimating the internal exposure of a chemical based on a given use-case scenario, and one for estimating the various PODs based on *in vitro* bioactivity data. Outputs from these modules are combined in the third module to estimate the BER. The approach is aimed at assessing systemic toxicity in adults; consideration of Development and Reproductive Toxicology will be addressed in a separate study.

The POD estimation module consists of 3 of the *in vitro* bioactivity platforms used in (Baltazar *et al.*, 2020) to obtain a BER estimate: high-throughput transcriptomics (or “transcriptomics” for brevity), a cell stress panel and *in vitro* pharmacological profiling. The latter 2 platforms were selected to cover off cellular stress and targeted biological effects, respectively, whereas the transcriptomics platform (generated using multiple cell models—HepG2, HepaRG, and MCF-7) was included to provide a nontargeted approach to capture biological effects potentially not detected using the other tools. Measurements for the cell stress and transcriptomics platforms are both performed 24 h after exposure to the test chemical. The data are then analyzed using concentration-response models to obtain the POD estimates in terms of the nominal concentration (ie, the total concentration expected to be present in the medium based on how the dosing solution was prepared). From this, a platform-specific POD is obtained per bioactivity platform for each chemical (referred to hereafter as the “platform POD”). Two different methods are used to estimate the transcriptomics platform: the global POD, as described in Reynolds *et al.* (2020) and the minimum BMDL (the lower bound of the pathway-average Benchmark concentration [Farmahin *et al.*, 2017]) obtained using BMDexpress2 (Phillips *et al.*, 2019). The exposure module includes PBK models that are used to estimate the plasma *C*_max_ for the benchmark chemical-exposure scenarios. In any *ab initio* risk assessment, there will be several sources from which PBK model parameters could be obtained, including *in silico* predictions, *in vitro* assays, and/or clinical measurements (Li *et al.*, 2022; Moxon *et al.*, 2020; Paini *et al.*, 2021; Punt *et al.*, 2022). These different sources can in turn affect the level of uncertainty that should be attributed to a particular PBK model prediction (where higher uncertainty is expected with models parameterized using only *in silico* predictions when compared with models calibrated against clinical data). The different parameter-estimate sources are represented as levels L1–L3, respectively. A separate Bayesian model, henceforth referred to as the *C*_max_ error distribution model, is therefore included in the toolbox to quantify the uncertainty in the *C*_max_ estimate, conditional on the parameter level being used. In the BER estimation module, the outputs from the *C*_max_ error distribution model are combined with the smallest (ie, most conservative) platform POD for a given benchmark chemical-exposure scenario to estimate a probability distribution for the BER.

### Overview of the Evaluation Approach

**Figure 2.**
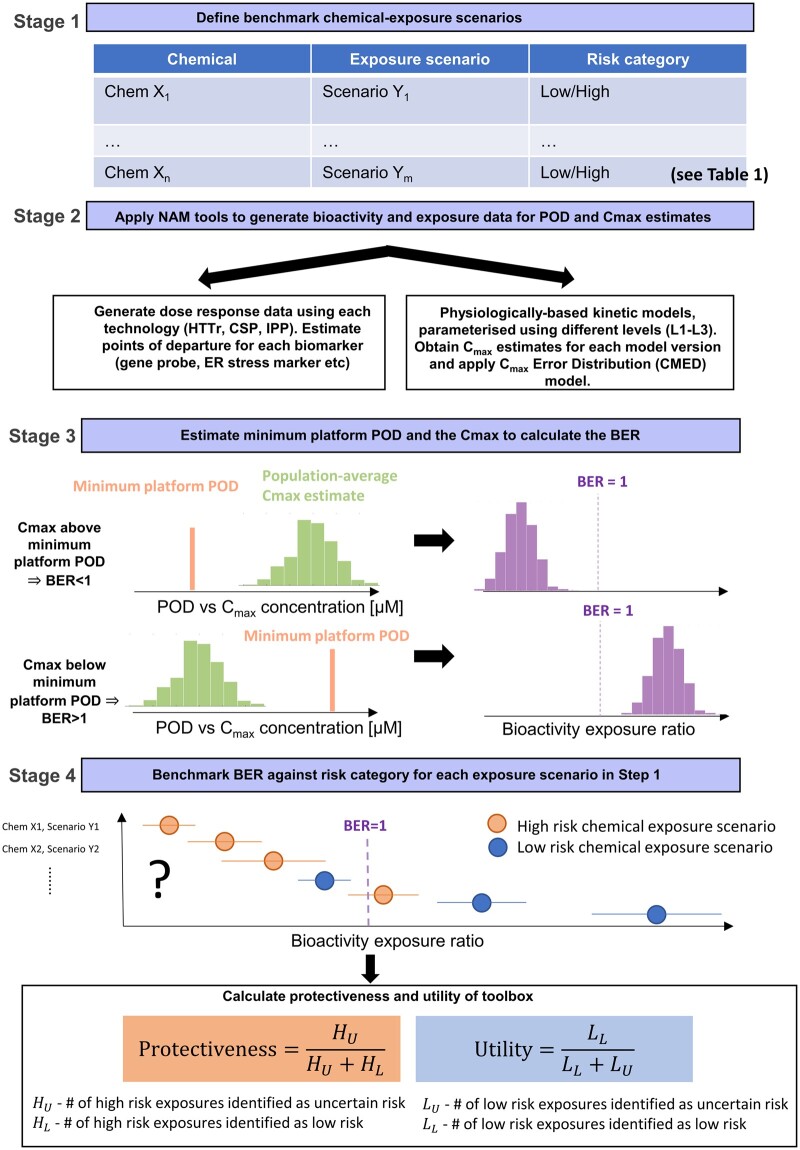
Overview of the proposed approach for evaluating the systemic safety toolbox and workflow. The approach is divided into 4 stages, involving the systematic generation and analysis of toolbox data for selected low- and high-risk benchmark chemical-exposure scenarios (Figure 1). At the final stage, bioactivity exposure ratio (BER) estimates are obtained for each chemical-exposure scenario. These BER estimates are used to understand (1) whether the BER can be used to correctly identify between low-risk benchmark exposures, based on a given decision model, and (2) to calculate the protectiveness and utility of the toolbox and thereby assess its overall performance. Results obtained in this study are used to establish a prototype decision model and associated performance metrics (ie, protectiveness and utility), which will then be used to assess the toolbox in the full evaluation.

The overall concept of the evaluation approach is to generate toolbox data (exposure estimates, PODs, and BER distributions) for a range of benchmark chemical-exposure scenarios that would either be considered low-risk (eg, existing food or cosmetic ingredients) or high-risk (eg, drugs that are systemically bioactive) from a consumer goods perspective, following the workflow as though each one were part of an *ab initio* risk assessment (Figure 2), and then assessing whether low risk scenarios can be correctly identified as such based on the toolbox BER estimates using an appropriate decision model. Here, chemical-exposure scenarios that could not be identified as low risk using the toolbox are regarded as “uncertain” risk, reflecting the fact that they could either correspond to a high risk or low risk scenario. In a real risk assessment, these chemical-exposure scenarios could be refined further using higher tier tools (which is beyond the scope of this work).

The evaluation approach comprises a 4-stage process, defined as follows. At stage 1, chemicals are selected where there is evidence that, for a defined exposure scenario, the chemical is likely to cause some form of systemic toxicity in humans (representing a high-risk exposure scenario), or for which there is a history of safe use supported by a conventional safety assessment (low-risk exposure scenario). At stage 2, concentration response bioactivity data (using high-throughput transcriptomics, the cell stress panel and *in vitro* pharmacological profiling) are generated for all the test chemicals identified at stage 1. Appropriate concentration ranges are established for each compound and each *in vitro* bioactivity platform (see the “*In vitro* bioactivity data” section). In parallel, internal exposure estimates are generated for each chemical-exposure scenario using PBK modeling. Here, *C*_max_ plasma estimates are obtained for all available parameterization levels (L1–L3, see the “Exposure estimation” section). The corresponding uncertainty in these predictions are then quantified using the *C*_max_ error distribution model. At stage 3, these results are combined to obtain probabilistic estimates of the BER for each chemical-exposure scenario at each PBK model parameterization level. At stage 4, the BER estimates and corresponding chemical exposure-scenario risk classifications that were assigned at stage 1 are combined to quantify the overall protectiveness and utility of the toolbox (see the “Decision making using BERs, protectiveness, and utility” section). The final stage of the evaluation depends on the selection of an appropriate decision model. As a first step toward this, the toolbox data generated in this work were used to explore and establish a prototype decision model (see below).

### Stage 1: Definition of Benchmark Chemical-Exposure Scenarios

At least 1 consumer exposure scenario was identified for each benchmark chemical from the literature to allow PBK modeling of either in-market or maximum use levels (foods, cosmetics), recommended treatment regimens (pharmaceuticals) or poisoning cases. A summary of the various benchmark chemical-exposure scenarios and associated risk classifications is provided in Table 1. Risk classifications of “high” or “low” were assigned to each benchmark scenario for the purpose of safety decision-making in the context of a consumer product (eg, personal care products). In other words, if the documented safety profile of the benchmark chemical-exposure was used as a decision for inclusion in a consumer product, it would be considered high or low risk accordingly.

**Table 1:** Overview of Each Chemical-Exposure Scenario and Associated Risk Classifications

Compound	Use Scenario	Risk Classification	Risk Classification Reasoning	Reference
Paraquat dichloride	Oral 35 mg/kg ingestion (poisoning)	High risk	The minimum oral human lethal dose is 35 mg/kg/day. Paraquat poisoning leads to multiorgan failure with specific pulmonary edema and fibrosis.	WHO, 1984; Bertram *et al.*, 2013
Rosiglitazone	Oral 8 mg/day	High risk	The maximum recommended daily dose for the treatment of diabetes is 8 mg/day. Rosiglitazone leads to adverse effects such as weight gain, anemia, fluid retention, and adverse effects on lipids. Importantly, fluid retention may exacerbate or lead to heart failure and other effects. A low dose of 2 mg/day shows some efficacy.	Wolffenbuttel *et al.*, 2000; Yki-Järvinen, 2004
	Oral 2 mg/day	High risk		
Doxorubicin hydrochloride	75 mg/m^2^/day infusion for 10 min	High risk	The incidence of symptomatic chronic heart failure is estimated to be 3%–4% after a cumulative dose of 450 mg/m^2^ if doxorubicin is administered as a bolus or short infusion of 45–75 mg/m^2^ every 3–4 weeks.	Biganzoli *et al.*, 2003; Injac and Strukelj, 2008; Lee *et al.*, 2010; Oakervee *et al.*, 2005; Rahman *et al.*, 2007
	4.5 mg/m^2^/day continuous infusion for 4 days, repeated every 3 weeks			
Butylated hydroxytoluene (BHT)	Dermal 0.5% in body lotion	Low risk	Used safely in cosmetic products and foods. Existent consumer risk assessment from the SCCS.	SCCS, 2021b
Oxybenzone	Dermal 2% in a sunscreen	Low risk	Used safely as a UV filter in cosmetic products. Existent consumer risk assessment from the SCCS.	SCCS, 2021a Regulation (EC) No 1223/2009
	0.5% in a body lotion	Low risk		
4-Hexylresorcinol	Oral throat lozenge (2.4 mg)	Low risk	Used safely as a throat lozenge. Antimicrobial and anesthetic effects are local only, supported by clinical data.	Matthews *et al.*, 2020; McNally *et al.*, 2012
	Dermal 0.5% face serum	Low risk	Used safely in cosmetic products. Exposure level supported by existent toxicological data.	EFSA Panel, 2014; Won *et al.*, 2014
	Oral food residue 3.3 µg/kg bw/day	Low risk	Existent consumer risk assessment from EFSA.	EFSA Panel, 2014
Caffeine	Oral dietary intake—400 mg/day	Low risk	No evidence for concern with respect to systemic toxicity from the available toxicological data, as concluded by EFSA, Health Canada, and the FDA.	Blanchard and Sawers, 1983; EFSA Panel on Dietetic Products, Nutrition and Allergies, 2015; Nawrot *et al.*, 2003
	Dermal 0.2% shampoo	Low risk		
	Oral tablets/overdose >10 g	High risk	Evidence of serious adverse systemic effects, which can result in death.	Jabbar and Hanly, 2013
	Dermal clinical (2 mg/cm^2^ of a solution containing 2.5% caffeine applied to a test area of 25 cm^2^)	Low risk	No evidence for concern with respect to systemic toxicity from the available toxicological data at this level, as concluded by EFSA, Health Canada, and the FDA. No reports of systemic effects from volunteers administered 1.25 mg topical caffeine as part of this clinical study.	Otberg *et al.*, 2008
Coumarin	Oral dietary intake 4.085 mg/day	Low risk	Used safely in flavorings and other food ingredients with flavoring properties. Existent consumer risk assessment from EFSA.	EFSA, 2008
	Oral dietary intake 0.1 mg/kg bw/day	Low risk		
	Dermal 0.38% as a fragrance in body lotion	Low risk	Used safely as a fragrance in cosmetic products. Maximum level supported by RIFM fragrance ingredient safety assessment in this product type.	Api *et al.*, 2020
Niacinamide	Tolerable daily intake (TDI) 12.5 mg/bw/day	Low risk	Used safely as a cosmetic ingredient and vitamin supplement. No evidence for concern with respect to systemic toxicity from the available toxicological data, as concluded by the Scientific Committee on Food and Scientific Panel on Dietetic Products, Nutrition and Allergies. Niacinamide is a form of vitamin B3 with a recommended intake of 10–15 mg/day of niacin equivalent.	Cosmetic Ingredient Review Expert Panel, 2005; EFSA NDA Panel, 2014; EFSA Panel on Nutrition, Novel Foods and Food Allergens, 2022
	Norwegian dietary intake 22.2 mg/day	Low risk		
	0.1% in a hair conditioner	Low risk		
	3% in a body lotion	Low risk		
Sulforaphane	Dietary intake 3.9 mg/day	Low risk	Long history of sulforaphane consumption in cruciferous vegetables. However, there is an uncertainty in the sulforaphane human exposure due to the variation of sulforaphane content across the different vegetables and its formation from glucoraphanin depending on how vegetables are prepared. The exposure selected, for which concentration in humans were available, was considered representative of a high consumption of broccoli in the U.K. population (18.5 g/day) and assumed a high concentration of sulforaphane in broccoli (37–75 mg per 100 g of fresh weight).	Hanlon *et al.*, 2009; Howard *et al.*, 1997
	Oral 20 mg 3× daily	Low risk	No evidence of systemic effects in patients given this regimen as part of a clinical trial.	Cipolla *et al.*, 2015

The risk classifications for each chemical-exposure scenario were performed based on the availability of existing toxicological information and evidence of systemic effects in humans at the listed exposures. Regulatory bodies such as European Food Safety Agency (EFSA), European Scientific Committee on Consumer Safety (SCCS), Research Institute for Fragrance Materials (RIFM), the U.S. EPA, and the U.S. Food and Drug Administration (FDA) provide comprehensive reviews of the toxicological information available for chemicals used as foods, cosmetics, and pesticides. Where available, data and conclusions from these reports were used to support the assigned risk classifications. Case reports and literature reviews were found that identified high risk levels for caffeine and paraquat dichloride that collated evidence of serious systemic effects, including the potential for death, following overdose and accidental ingestion, as listed in Table 1. For benchmark chemicals administered as a drug intended to be systemically bioactive, it was considered that although there is an inherent risk of adverse effects for a proportion of patients, even at the therapeutic dose, such pharmaceuticals are only administered where the benefit outweighs the risk, and hence were classified as high risk by default. Notably, there were no systemic effects, intended or unintended, for the administration of sulforaphane to post-surgery males in the clinical trial identified, and therefore this is classified as low risk from a consumer goods perspective (Cipolla *et al.*, 2015).

### Stage 2: Exposure Estimation

#### PBK Modeling

PBK models were developed using GastroPlus 9.8 (Simulation Plus, Lancaster, California), which is a specialist modeling software that includes modules to simulate several administration routes including intra-venous, oral, and dermal absorption. Model parameters include logP (logarithm of octanol-water partition coefficient), water solubility, unbound fraction in plasma (*f*_up_), blood: plasma ratio (*R*_bp_), hepatic intrinsic clearance (CL_int_), Madin-Darby canine kidney (MDCK) permeability (*P*_app_), pKa (logarithm of acid dissociation constant), and intestinal absorption or skin penetration, where applicable. Details on the modeling assumptions used by Gastroplus for intestinal absorption and skin penetration are provided in Supplementary Information S1, M1. Models were built for each chemical-exposure scenario following the tiered framework defined in Moxon *et al.* (2020). Adult consumers (the focus of the study) were represented by 60 kg adult female. This was selected as it was considered conservative both in terms of body weight, and potential use of cosmetics (SCCS, 2021c). The framework is split into several levels of increasing complexity and refinement depending on how the models are parameterized:

- *Level 1 (L1):* *in silico* only: chemical specific parameters are obtained using only *in silico* predictions
- *Level 2 (L2):* *in silico* and *in vitro:* values for logP, pKa, solubility, hepatic intrinsic clearance, unbound fraction in plasma, blood: plasma ratio, and intestinal absorption or skin penetration are all obtained from *in vitro* measurements when available. All other parameters are obtained using *in silico* predictions.
- *Level 3 (L3)*: *in silico*, *in vitro*, and clinical data: similar to L2 chemical-specific parameters are obtained (where available) from *in vitro* measurements. Clinical data are then used to further refine estimates of key parameters through a process of model calibration. The selection of the parameters to be calibrated is based on sensitivity analysis results and expert judgement. In every case, the clinical data used in the calibration of a PBK model was for different exposure scenario other than the one being modeled. For example, when generating the L3 dermal caffeine exposure predictions (see Table 1), the model was first calibrated against clinical data from a separate caffeine intravenous infusion study (see Supplementary Information S1, T1 for details).

*In silico* parameter estimates were sourced using ADMET Predictor (v.9.0). *In vitro* and clinical data used for the L2 and L3 model parameterizations were sourced from the literature (see Supplementary Information S1, T1 for references). With predicted properties, classification was conducted using the extended clearance classification system, which provides information about the dominant route of clearance from the body (Varma *et al.*, 2015). If the dominant clearance mechanism was predicted to be renal, the kidney clearance rate was determined by the formula *f*_up_ × GFR, otherwise kidney clearance was set to zero. Tissue-to-plasma partitioning coefficients (*K*_t:__p_) was calculated in GastroPlus using the Berezhkovskiy method (Berezhkovskiy, 2004; Lukacova *et al.*, 2008), assuming chemical distribution into the tissues is perfusion limited. Dermal administration was modeled with the mechanistic dermal absorption module in GastroPlus. A set of diffusion and partitioning coefficients parameters of the chemical in various skin layers (ie, the stratum corneum, epidermis, and dermis) was either predicted based on the input physicochemical properties or fitted against available *ex vivo* skin penetration experiments (Li *et al.*, 2022).

PBK simulations were performed at each parameterization level (L1–L2, and where possible, L3), from which corresponding *C*_max_ estimates were obtained (see Supplementary Information S1, T1).

#### C_max_ Uncertainty Quantification

##### C_max_ error distribution model specification

Uncertainty in PBK *C*_max_ estimates was quantified using an inductive approach, whereby a Bayesian *C*_max_ error distribution model was used to learn the distribution of the prediction errors at each PBK level. This was done using a dataset that comprised exposure scenarios for which either measured *C*_max_ taken from clinical studies, or PBK *C*_max_ estimates (or both) were available. The purpose of the model is that, once conditioned on the available data, it could then be used to estimate the distribution of the prediction error of *C*_max_ for new chemicals or exposure scenarios not in the dataset by assuming the prediction error for a new chemical can be considered a sample from the distribution of errors observed for previous chemicals and scenarios. For the purposes of modeling, PBK predictions and measured *C*_max_ values are assumed to be transformed to their base-10 logarithm (ie, log10). The major assumptions underpinning the *C*_max_ error distribution model are:

1. The target of the prediction is the population average *C*_max_.
2. Measured log10(*C*_max_) values (eg, from a clinical study) are normally distributed with respect to the true (unobserved) population average log10(*C*_max_), with small variance.
3. PBK parameterization L3 predictions are normally distributed with respect to the true (unobserved) population average log10(*C*_max_).
4. PBK parameterization L2 log10 predictions are normally distributed with respect to the L3 log10 predictions.
5. PBK parameterization L1 log10 predictions are normally distributed with respect to L2 log10 predictions.
6. The variance between PBK log10 predictions at adjacent levels (eg, L1 and L2) is dependent on the number of shared parameters between the 2 levels.
7. The distribution of prediction errors at each level is exposure and chemical agnostic, only the parameterization level (ie, L1, L2, and L3) is assumed to be important.

A consequence of these assumptions is that for 2 different chemical-exposure scenarios with the same parameterization level (eg, L2, with both having 2 parameters informed using *in vitro* data, etc.), the variance of the predictive distributions would be the same (although the mean would be different).

##### Model equations

The notation ${\hat{\zeta }}_{i,j}^{\text{clinical}}$ is used to denote the base-10 logarithm of the measured *C*_max_ value for chemical i under exposure j, and $\zeta_{i,j}$ denotes the base-10 logarithm of the true (unobserved) corresponding population average *C*_max_. From assumption 1, *C*_max_ estimates follow a Gaussian (normal) distribution with mean $\zeta_{i,j}$ and standard deviation $\sigma_{\text{clinical}}$, so that the sampling distribution is expressed as:

$${\hat{\zeta }}_{i,j}^{\text{clincial}}∼N\left(\zeta_{i,j},\sigma_{\text{clinical}}\right).$$

From assumption 2, it is assumed that clinical study estimates of the true population-average *C*_max_ are at best to within an error of 10% (1.1-fold) with probability .95 and at worst an error to within 2-fold with probability .95, so that a credible range for $\sigma_{\text{clinical}}$ would be [0.0225, 0.155]. The prior distribution for $\sigma_{\text{clinical}}$is therefore chosen as

$$\sigma^{\text{clincal}}∼\text{InverseGamma}\left(4.6,0.22\right)$$

to capture this.

From assumption 3, the sampling distribution for a L3 PBK estimate (the log base 10 value of which is denoted by ${\hat{\zeta }}_{i,j}^{L3}$), conditional on the true population average *C*_max_ is given by

(1) $${\hat{\zeta }}_{i,j}^{\text{L3}}∼N\left(\zeta_{i,j}+\alpha_{L3},\sigma_{L3,\text{pop}}\right),$$

where $\alpha_{L3,\text{pop}}$ is the standard deviation of the L3 PBK estimate error and $\alpha_{L3}$ is included to capture any bias in predictions.

According to assumptions 4 and 6, the variance of the difference between PBK predictions at adjacent levels is modeled as an increasing function of the number of parameters with different values (if all parameters were shared, the difference is zero). The sampling distribution of a L2 PBK estimate (denoted by ${\hat{\zeta }}_{i,j}^{L2}$), conditional on the L3 PBK estimate is modeled as

$${\hat{\zeta }}_{i,j}^{\text{L2}}∼N\left({\hat{\zeta }}_{i,j}^{\text{L3}}+\alpha_{L2},\frac{\sigma f_{i,j}^{L2,L3}}{\beta +f_{i,j}^{L2,L3}}\right),$$

where $f_{i,j}^{L2,L3}$ is the fraction of parameters with different values between L2 and L3, β and σ control the rate of growth of the standard deviation of the difference in predictions as the number of parameters grows and $\alpha_{L2}$ encodes any bias between PBK L2 and L3 predictions.

For the established set of predictions, the maximum proportion of parameters which change values between levels 2 and 3 is 0.38. In the case that we are predicting an unobserved L3 prediction, where the parameters which would be calibrated is unknown, we set $f_{i,j}^{L2,L3}=0.5$ to maximize the standard deviation whilst staying reasonably close to the maximum number of parameters which are modified at L3.

From assumptions 5 and 6, the same structure is used to model the sampling distribution of L1 predictions, conditional on L2 predictions:

$${\hat{\zeta }}_{i,j}^{\text{L1}}∼N\left({\hat{\zeta }}_{i,j}^{\text{L2}}+\alpha_{L1},\frac{\sigma f_{i,j}^{L1,L2}}{\beta +f_{i,j}^{L1,L2}}\right),$$

where $\alpha_{L1}$ encodes for any bias between L1 and L2 predictions. Between levels 1 and 2, typically, a larger proportion of parameters change value than between L2 and L3. Therefore, when it is unknown how many PBK parameters are informed by *in vitro* data at L2, we set $f_{i,j}^{L1,L2}=1$ for the purposes of defining the standard deviation of an L1 prediction conditional on an L2 prediction.

To complete the model, the following prior distributions are used to regularize parameter estimates:

$$\begin{array}{c} \alpha_{k}∼\text{HalfNormal}\left(0,0.5\right)\;\text{for}\;k\in \{L1,L2,L3\},\\ \sigma_{L3,\text{pop}}∼\text{HalfNormal}\left(0,1\right)\;,\\ \sigma \;∼\text{HalfNormal}\left(0,1\right)\;,\\ \beta \;∼\text{HalfNormal}\left(0,0.5\right)\;. \end{array}$$

##### Data processing and model fitting

The training set for the *C*_max_ error distribution model was constructed as follows. PBK *C*_max_ estimates for all parameterization levels (L1–L3) and clinically measured *C*_max_ values were obtained (where possible) for a total of 30 chemical-exposure scenarios listed in Supplementary Information S1, T2. This set includes the 24 exposure scenarios listed in Table 1, together with additional exposure scenarios included purely for the training of the Bayesian model (they were not included in the BER analysis due to either lack of bioactivity data or a suitable risk classification). This includes PBK *C*_max_ for 4 dermal exposures (obtained at parameterization levels L1–L3) obtained from Li *et al.* (2022) and 2 exposure scenarios for valproic acid. Clinically measured *C*_max_ estimates were only available for 11 of the scenarios. Overall, for 6 of the 30 exposure scenarios (those for hexylresorcinol, paraquat, and sulforaphane), L3 PBK model predictions were not possible as suitable clinical data for model calibration were not available.

The posterior distribution of the model parameters was evaluated using Monte Carlo Markov chain algorithms implemented in the probabilistic programming language Stan (Carpenter *et al.*, 2017). Missing L3 and measured *C*_max_ values were included as parameters within the model, the values of which were inferred during the fitting process. Data processing and visualization was performed using Python 3.8 with packages PyStan v2.19, NumPy v1.19, SciPy v1.6, pandas v1.2, and matplotlib v3.3. The predictive distributions were then generated by drawing from the posterior distribution.

##### Model evaluation

The predictive performance of the *C*_max_ error distribution model was evaluated using a leave-one-exposure-out strategy, whereby for the 11 exposure scenarios where measured *C*_max_ was available, the ability of the model to predict measured *C*_max_ following its removal from the training set was assessed. This was done for all parameterization levels where a PBK *C*_max_ estimate was available. To generate a prediction of the measured *C*_max_ value at level *X* of the framework (for a given exposure scenario), the corresponding clinically measured *C*_max_ was removed along with PBK *C*_max_ estimates at levels *X* + 1 and higher. The model was then retrained on the reduced dataset and the predictive distribution for the clinically measured *C*_max_ was compared against the withheld value. This process was repeated separately for each chemical-exposure scenario with a clinically measured *C*_max_ estimate (ie, for each of the PBK estimates for that exposure scenario, at each parameterization level).

### Stage 2: *In Vitro* Bioactivity Data

#### Experiments

##### Materials

Test chemicals were purchased from Sigma-Aldrich (Dorset, UK), LGC Standards, and Cambridge Bioscience. The identity and purity were confirmed by 1H-NMR, 13C-NMR, LC-MS, and HPLC conducted at Selcia Lab. The same batch of chemical was tested across all the bioactivity assays mentioned below.

##### Dose confirmation

Based on their physicochemical properties, 7 chemicals were identified as potentially difficult to test, ie, challenges were anticipated with regards to achieving nominal concentration under *in vitro* assay test conditions due to potential binding to plastic surfaces, losses due to volatility, or low solubility. Typically, these issues are not considered in *in vitro* NAM studies investigating the bioactivity of chemicals, but were considered here due to the quantitative nature of the data and the need to ensure PODs can confidently be used in decision-making. To confirm the total medium concentration attained under typical test conditions, bioanalytical methods were developed at Charles River Laboratories to determine concentrations of butylated hydroxytoluene, coumarin, doxorubicin, oxybenzone, paraquat dichloride, sulforaphane, and valproic acid in HepG2 medium (DMEM supplemented with 10% FBS, 25 µg/ml penicillin and 25 µg/ml streptomycin). Mass spectrometric detection of the test compound was optimized and appropriate chromatography conditions derived. Sample plates (separate to the bioactivity assays) were prepared at Cyprotex and stored frozen at −80°C until transport to Charles River Laboratories for analysis. Sample preparation was performed by the precipitation of matrix proteins with solvent, followed by centrifugation and analysis of compound recovered in the supernatant by ultra-performance liquid chromatography—tandem mass spectrometry (UPLC-MS/MS).

##### Concentration range setting

The maximum concentration to be tested in the cell stress and the transcriptomics platforms were set for each compound and cell line using an initial cytotoxicity prescreen using Cellular ATP and LDH release measurements (see Supplementary Information S1, T3 and Supplementary Information S2: Cytotoxicity). These results then used to set the *in vitro* pharmacological profiling screening concentrations (see below). For the cell stress and the transcriptomics platforms, a standardized concentration setting procedure was used which involved defining the maximum concentration based on the minimum of either a chemical’s solubility limit or the concentration at which cytotoxicity is observed. The dilution series used for each chemical was such that the minimum concentration was approximately 4 orders of magnitude smaller than the maximum concentration. The cell stress and transcriptomics platform dilution series for each chemical are given in Supplementary Information S1, T4.

##### *In vitro* pharmacological profiling

The *in vitro* pharmacological profiling platform contains 63 targets with known safety liabilities that were tested in binding, enzymatic, coactivator recruitment, and luciferase assays. Forty-four of the targets have been associated with *in vivo* adverse drug reactions (Bowes *et al.*, 2012) and they include: 24 G-protein-coupled receptors (GPCRs), 7 enzymes, 2 nuclear receptors, 8 ion channels, and 3 transporters. A further 19 targets implicated in developmental toxicity were added to the panel based on a literature search (Escher *et al.*, 2022; OECD, 2020; Wu *et al.*, 2013). They included 15 nuclear receptors, 2 enzymes, 1 GPCR, and 1 structural protein. For the full list of the *in vitro* pharmacological profiling targets and associated assays, refer to Supplementary Information S2.

Screening was initially performed using a fixed concentration of each chemical in 2 replicates. The standard accepted default concentration for this type of assay is 10 µM (Jenkinson *et al.*, 2020). However, for 6 of the test chemicals (caffeine, coumarin, niacinamide, oxybenzone, paraquat, and valproic acid), cytotoxicity was only detected at concentrations over 100 µM (based on the cellular-ATP and LDH release concentration-response data, as described above). In this case, the screening assays were repeated at 100 µM to ensure potentially important safety liabilities were not missed.

Screening assays that showed specific binding of the chemical greater than 50% relative to the control agonist/antagonist binding were followed-up through the generation of concentration-response data. The concentration-response was carried out at 8 concentrations in 2 technical replicates. The choice of concentrations was informed by the percent of inhibition/stimulation from the screening phase so that both plateaus of the concentration-response curves could be experimentally observed.

##### High-throughput transcriptomics

Sequencing high-throughput transcriptomics was performed using TempO-Seq (BioClavis) version 2 of the human whole transcriptome panel. HepG2, MCF7, and HepaRG cells (all in a 2D format, see Supplementary Information S1, T5) were treated for 24 h with 7 concentrations of each chemical using 0.5% DMSO as a solvent control (see Supplementary Information S1, T4).

Following treatment, cells were washed in calcium and magnesium-free PBS. After removal of all residual PBS, 2× TempO-Seq lysis buffer (BioSpyder Technologies, proprietary kit) was diluted to 1× with PBS and added at a volume of 1 µl per 1000 cells with a minimum of 10 µl per well and incubated for 10 min at room temperature. Following lysis, the samples were frozen at −80°C prior to sequencing.

TempO-Seq analysis was performed as described previously (Yeakley *et al.*, 2017), with a targeted sequence depth of 200 mapped read counts per transcript including the use of the general attenuation panel. Raw count data were produced using the STAR algorithm (Dobin *et al.*, 2013) and TempO-Seq R software package.

##### Cell stress panel

All compounds were tested using the recently developed cell stress panel (Hatherell *et al.*, 2020). The panel comprised biomarkers that cover 8 key stress pathways (Simmons *et al.*, 2009), mitochondrial toxicity, and general cell health. The panel (Supplementary Information S1, T3) was expanded to include the biomarkers phospho-p53 (DNA damage), SRXN1 (oxidative stress), and NFAT5 (Osmotic stress) for broader coverage of the cell stress pathways. In this data set, 3 biological replicates were performed for each assay with 2 technical replicates per concentration tested along with an increased number of DMSO controls on the plate to control for within-plate effects when conducting concentration-response analysis, as described in Hatherell *et al.* (2020) (see Supplementary Information S1, M2 for the plate layout). HepG2 cells were treated for 24 h at 8 concentrations (Supplementary Information S1, T4 and T5).

#### Point of Departure Estimation

##### *In vitro* pharmacological profiling

PODs for the *in vitro* pharmacological profiling platform comprised EC_50_ values (concentration producing a half-maximal response) and IC_50_ values (concentration causing a half-maximal inhibition of the control agonist response). These were obtained for all targets for a given chemical identified during the screening phase (see above). The screening data for butylated hydroxytoluene, niacinamide, and sulforaphane were negative in all assays and so no *in vitro* pharmacological profiling PODs were obtained for these chemicals. For all other chemicals, the platform POD was given by the minimum EC_50_ or IC_50_.

The EC_50_ and IC_50_ were determined using a Bayesian model of the concentration-response curves that were modeled using the Hill equation (Labelle *et al.*, 2019). The priors for IC_50_ were set to the median experimental concentration, the slope was set to 1.0 and low and high concentration responses were set to 0% and 100%, respectively.

##### BIFROST analysis of high-throughput transcriptomics and cell stress panel data (global POD)

High-throughput transcriptomics and cell stress panel concentration-response data were analyzed using a novel Bayesian method. The approach is here-on referred to as the BIFROST method (Bayesian inference for region of signal threshold). BIFROST is used to estimate the “global POD,” which represents an estimate of the minimum effect concentration across all genes (transcriptomics data) or biomarkers (cell stress panel), for a given chemical. The method quantifies uncertainty in the POD as a probability distribution for each biomarker or gene analyzed. Up to 100 or so, PODs may be obtained from the cell stress panel and potentially 1000s of gene-level PODs may be obtained from the transcriptomics data, per chemical. The BIFROST method uses all individual distributions of PODs to calculate the global POD. The BIFROST method was first published for the analysis of cell stress panel data in Hatherell *et al.* (2020) and later for the analysis of transcriptomics data in Reynolds *et al.* (2020). Briefly, the approach aims to construct a hierarchical description of the different sources of variance in a concentration-response dataset. Sources of variance may include, for example, the effect of a treatment, biological variance, technical variance, batch effects as well as platform-specific variance such as sampling variability for reads in the transcriptomics data. These considerations make full specification of the model specific to a particular experimental design. Full details of the method as applied to both cell stress panel and transcriptomics data are provided in Supplementary Information S1, M2 and M3, respectively.

##### BMDExpress2 analysis (BMDL)

Raw counts were processed using the R package DESeq2 (Love *et al.*, 2014) separately per chemical/cell-line dataset. Probes were filtered to include only those which had a median count, across all samples, of 5 or above and samples were filtered to only include those with more than a sum of 2.5 million counts within the remaining probes and with a mapped read percentage over 55%. Outliers were removed where biological replicates had a correlation of <85% and could identified using principal component analysis.

Data were normalized using the negative binomial distribution in DESeq2 with model “∼ VESSEL_ID + CONCENTRATION” where “VESSEL_ID” is given per treatment 384 well plate and is identified as a strong source of variation between biological replicates, especially in HepaRG cells, and therefore set as a confounding factor. Rlog-transformed normalized counts were used as input into benchmark response (BMR) modeling software BMDExpress2 (Phillips *et al.*, 2019) where data were modeled to calculate PODs per chemical/cell-line dataset. Within BMDExpress2, probes were first filtered for a significant concentration response using a Williams Trend Test with threshold *p* < .05 and minimum fold change of 1.5 across concentrations tested. The data were then modeled using 6 parametric models (Poly 2, Hill, Power, Exponential 3, 4, and 5, with recommended default configurations). Benchmark concentration (BMD) values with upper (BMDU) and lower (BMDL) confidence interval bounds were determined for each probe based on a BMR factor of 10% using the model which produced the lowest Akaike information criterion value. (note here “D” is being used in the various acronyms instead of “C,” which reflects the fact that the methodology was originally developed for *in vivo* dose studies, even though the transcriptomics data was concentration based). Pathway enrichment analysis was performed within BMDExpress2 using probes which had (1) a BMD between 10-fold less than the lowest tested concentration and the highest concentration tested; (2) BMD upper to lower ratio less than 40; and (3) a model fit *p* value more than .1. Pathways were deemed to be significantly enriched if pathways had a 2-tailed fishers *p* value less than .1, over 2 probes in the input data set were found in the pathway and 1 or more probes in the pathway passed the previously listed probe significance criteria. The mean BMDL was calculated by taking the mean of all significant probe level BMDLs in the given Reactome pathway. For each chemical-treated cell line, the lowest pathway mean BMDL was determined as the POD, and so the POD defined here is the lowest observed concentration that shows significant pathway perturbation (Farmahin *et al.*, 2017).

### Stage 3: Bioactivity Exposure Ratios

BERs were defined as the ratio between the minimum platform POD (or simply, the minimum POD) and the estimated *C*_max_ distribution. The set of possible platform PODs from which the minimum was computed were:

1. The minimum IC_50_ or EC_50_ from the *in vitro* pharmacological profiling platform (if available).
2. The global POD from the cell stress panel when analyzed using the BIFROST method.
3. The global POD from the transcriptomics platform (1 for each cell line), obtained using the BIFROST method.
4. The minimum pathway BMDL from the transcriptomics platform (1 for each cell line), obtained using BMDExpress2.

The BER distributions were then constructed each chemical-exposure scenario and associated PBK parameterization level (L1–L3) as follows. Random samples of the estimated *C*_max_ were drawn from the posterior the *C*_max_ error distribution model for the corresponding exposure scenario and PBK level. For each *C*_max_ sample (denoted $C_{i}$), a corresponding BER sample value (denoted $B_{i}$) was calculated as $B_{i}=P/C_{i}$, where P is the minimum platform POD for the relevant chemical. Various statistics for a given BER distribution (estimated values, credible intervals, etc.) were then computed from the $B_{i}$ samples.

When calculating the BER distributions for different subsets of platform PODs, the distributions were calculated as described above, the only difference being that minimum platform POD was instead calculated across the given subset.

### Stage 4: Decision-Making Using Bioactivity Exposure Ratios: Protectiveness and Utility

To decide whether an exposure is low risk or not, based on the BER distribution, a confidence threshold was first set (*p*_threshold_) such that an exposure was regarded as low risk if the probability that the BER >1 exceeded *p*_threshold_ (ie, if Prob.(BER > 1) > *p*_threshold_). Otherwise, it was regarded as uncertain risk (to reflect the fact that in reality, the exposure scenario could either be low-risk or high-risk). Different *p*_threshold_ values were explored for each PBK level. In summary, the following prototype decision model was considered for the toolbox, so that for a given PBK level:

1. Classify an exposure as low risk if the probability of the BER exceeds 1 is above *p*_threshold_.
2. Classify an exposure as uncertain risk if the probability the BER is greater than 1 is below *p*_threshold_.

Given the decision model described above, the protectiveness of the toolbox was defined as the proportion of high-risk exposures classified as uncertain risk (ie, not classified as low risk), whereas the utility of the toolbox was defined as the proportion of low-risk exposures correctly identified as low risk. The equations defining these 2 metrics are provided in Figure 2, stage 4. It is important to note that although definitions for protectiveness and utility are similar to that of sensitivity and specificity used with binary classifiers, they are not directly equivalent. This is because chemical-exposures scenarios not identified as low risk are identified as uncertain risk (ie, the exposure could be either high or low risk), rather than high risk (which would be the case if the toolbox was equivalent to a standard binary classifier).

### Data Repository

Raw experimental data for the 3 bioactivity platforms, together with detailed reports for the high-throughput transcriptomics and cell stress panel analysis, and a summary of the PBK model predictions, are provided through the Dryad digital repository, available at: https://doi.org/10.5061/dryad.fbg79cnx1.

## RESULTS

###  

#### Estimating Benchmark Exposures Using Physiologically Based Kinetic Models

To exemplify the toolbox evaluation approach outlined in Figure 2, each stage of the evaluation was performed for the 24 low-risk and high-risk benchmark chemical-exposure scenarios defined at stage 1 of the evaluation (see Table 1). As part of stage 2, corresponding PBK *C*_max_ estimates needed to be generated for each scenario. However, to reflect how the toolbox might be used within a real *ab initio* risk assessment, consideration was given to the different information sources that could be used to parameterize the PBK models. Following Li *et al.* (2022) and Moxon *et al.* (2020), 3 levels of parameterization were identified: L1 (level 1: parameters are based solely on *in silico* predictions), L2 (level 2: parameters are informed through a combination of *in silico* predictions and *in vitro* data), and L3 (level 3: as with L2, but where clinical data, obtained for an exposure scenario other than the one being modeled, is used to further refine the estimates of key model parameters). L1 corresponds to an initial estimate of the systemic exposure to a chemical within a risk assessment, whereas L2 and L3 are intended to provide additional refinements (if required) to the L1 and L2 estimates, respectively. Overall, the intention is that the PBK estimates become more accurate as the levels are ascended. However, for a novel chemical, it is unlikely that the clinical data required for L3 would be available, and so risk assessments would initially be restricted to L1 and L2. To explore the relative accuracy of the different parameterization levels, and how this may impact decision-making, PBK estimates were obtained for each chemical-exposure scenario in Table 1, together with additional exposure scenarios from Li *et al.* (2022) (see Materials and Methods), at parameterization levels L1, L2, and (where possible) L3. This was supplemented with corresponding measured *C*_max_ values from relevant clinical studies (when available).

**Figure 3.**
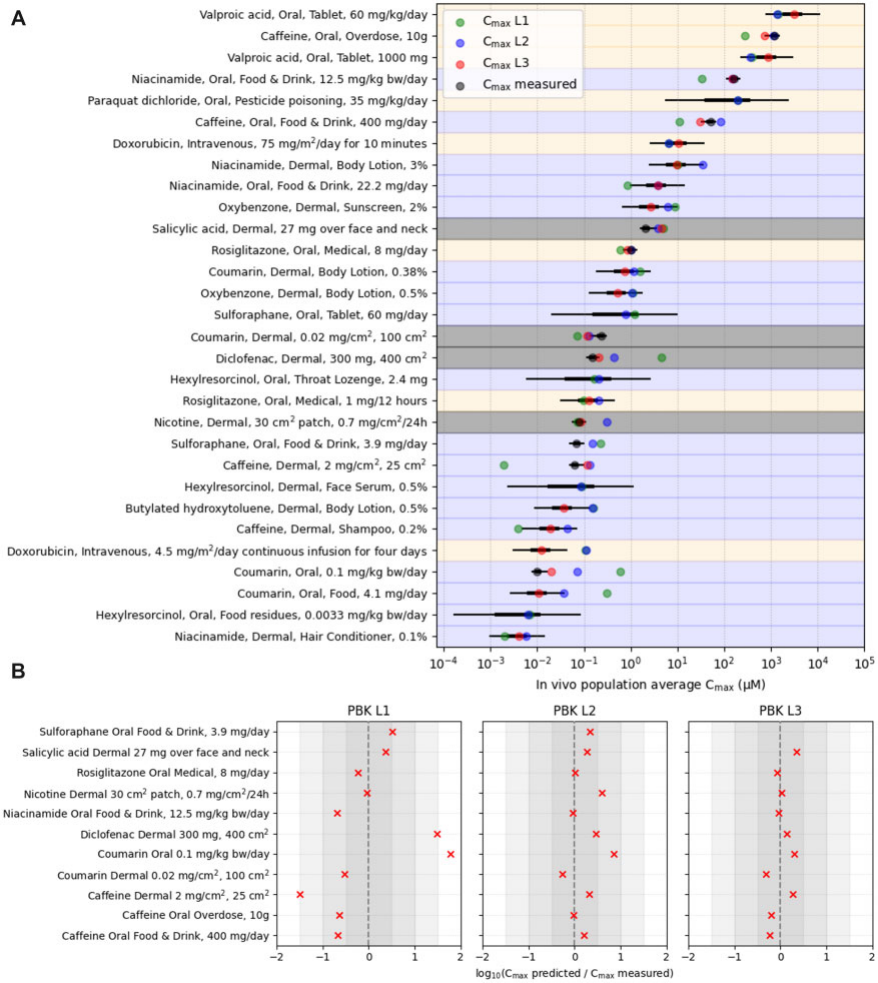
Overview of the physiologically based kinetic (PBK) model estimates and *C*_max_ error distribution model results. A, Distributions representing the uncertainty of the population average *C*_max_, conditional on all available exposure information, for all exposure scenarios used to train the *C*_max_ error distribution model. Thin lines cover a centered 95% interval and thick lines a 50% interval of the distribution. The distribution variance is smallest when the measured *C*_max_ is available for the exposure scenario (gray points). The variance is largest when only L1 and L2 PBK estimates are available (green and blue, respectively). Background colors indicate the risk category for each benchmark chemical-exposure scenario assigned at stage 1 (blue—low, orange—high). The 4 dermal exposure scenarios from Li *et al.* (2022) (used only for training the Bayesian model) are indicated in gray. B, A comparison between *C*_max_ PBK estimates at different parameterization levels and the corresponding measured *C*_max_ values (for the 11 exposure scenarios where these values were available), provided in terms of a ratio between estimated and measured *C*_max_ (red crosses). The shading indicates how far the ratio is from 1 (given by the vertical dashed line). Crosses to the left of the dashed line correspond to *C*_max_ values that were underpredicted by the PBK models, whereas to crosses to the right correspond to values that were overpredicted.

A complete summary of the PBK model predictions and measured *C*_max_ values are provided in Supplementary Information S1, T2 along with accompanying references. The results are also summarized in Figure 3A, where the risk categories assigned to each benchmark chemical-exposure scenario are also indicated. Overall, the estimated *C*_max_ values range from 0.004 to 4000 µM across all benchmark chemical-exposure scenarios considered. Here, larger *C*_max_ values tend to be associated with “high risk” benchmark classifications whilst the lower exposures are associated with “low risk” benchmark classifications. Thus, for the benchmark exposures considered in this work, levels of systemic exposure alone offered a moderate degree of separation between high and low risk exposures in the absence of any potency information (ie, PODs) from the bioactivity platforms.

The results show that the estimated values of the chemicals not only differ among different dosing and exposure routes, but also differ at the 3 PBK levels, showing that different parameterizations have a large influence on the outcome. A comparison between the 11 measured *C*_max_ values that were available and the corresponding PBK *C*_max_ estimates indicated that the PBK predictions become more accurate as the parameterization levels are ascended (Figure 3B).

#### Quantifying Uncertainty in PBK *C*_max_ Model Estimates

Overall, for a novel chemical and exposure scenario (ie, where no corresponding measured clinical data are available), a greater degree of uncertainty should be associated with an L1 prediction than an L2 or an L3 prediction. Quantifying the magnitude of these uncertainties remains a challenge, and to this end a Bayesian *C*_max_ error distribution model was developed that could provide a probabilistic description of the associated errors. The approach uses inductive reasoning, wherein the model is trained on 1 set of data (ie, the PBK *C*_max_ estimates and clinical measurements in Supplementary Information S1, T2) to obtain a posterior distribution for each of the Bayesian model parameters, which are then used to make predictions about the error associated with PBK *C*_max_ estimates not in the training set. In practice, the model essentially places a normal distribution around the base-10 logarithm of the PBK *C*_max_ estimate (ie, so that the logarithm of the PBK estimate corresponds to the mean of the distribution), with the variance of the distribution reflecting the error induced at the relevant PBK level, which is learned via the training set.

After training the model on the data in Supplementary Information S1, T2, predictive distributions of the population average *C*_max_ were generated for each exposure scenario (Figure 3A). The distributions represent a probabilistic estimate of what the “true” *C*_max_ is based on all available data. The variance of the distribution for each exposure scenario reflects the inferred precision of the estimate (ie, the size of the error between the PBK estimate and the “true” value), with smaller variances indicating more precise estimates. Overall, the precision associated with a given exposure scenario is dependent on what the highest PBK level was in the training set. There are 11 exposures for which measured *C*_max_ is available; these have the most precise *C*_max_ estimates overall. A further 14 exposures have PBK *C*_max_ estimates at all 3 levels, but no direct measurements of *C*_max_ are available; these estimates are inferred by the model to be less precise. Finally, there are 5 exposures which only have PBK estimates at L1 and L2 (ie, all the exposure scenarios associated with associated with hexylresorcinol, sulforaphane, and paraquat); these estimates are inferred to be the least precise overall.

The distributions obtained from the model can be considered fit for purpose only if they can be demonstrated to be well-calibrated (Dawid, 1982). For example, if a 95% prediction interval is generated for many exposure scenarios, that interval should contain the quantity of interest (eg, the true population average *C*_max_ value) with a frequency of 95%. Calibration was assessed using a leave-one-exposure-out strategy (see Materials and Methods). Rather than predict the population average *C*_max_ (which is unobserved), predictive distributions were generated for every measured *C*_max_ value after its removal from the training data. This was done for each PBK level for all 11 exposure scenarios where measured *C*_max_ was available (making a total of 32, noting that there was no L3 PBK estimate for the sulforaphane oral exposure scenario). Prediction intervals from these distributions are presented in Supplementary Information S1, F1. The 95% prediction interval covers 32/32 (100%) of the measured values. Fifty percent prediction intervals cover 18/32 (56%) of the measured values. Overall, these results indicate that the prediction intervals are largely in agreement with corresponding empirical frequencies, implying that the prediction intervals provided by the model for a given PBK estimate do reflect reasonably well how likely it is that they do in fact cover the true clinical *C*_max_ value (ie, 95%, prediction interval should cover the true value 95% of the time, etc.).

In general, for models that are well-calibrated, narrower prediction intervals indicate more precise estimates. Precision of the predictions at each PBK parameterization level can be viewed in the frame of how much the upper interval endpoint (eg, the 95th percentile), which may serve as an upper bound to an uncertain estimate, exceeds the measured value. The 95th percentile of the inferred distribution from L1 PBK estimates exceeds the measured *C*_max_ as much as 5100-fold with a geometric mean (across the set of all predictions) of 76-fold. At L2, the 95th percentile exceeds the measured value up to 99-fold with a geometric mean of 23-fold. At L3, the exceedance is as much as 9.0-fold with a geometric mean of 4.0-fold. These results, based on the 11 chemical-exposure scenarios for which measured *C*_max_ was available, indicated that the PBK estimates become more precise with higher model parameterization levels, consistent with what could be expected, and that (for this dataset) the *C*_max_ error distribution model provides a reasonable quantification of the associated PBK model estimation errors.

#### Points of Departure Across *In Vitro* Bioactivity Platforms

**Figure 4.**
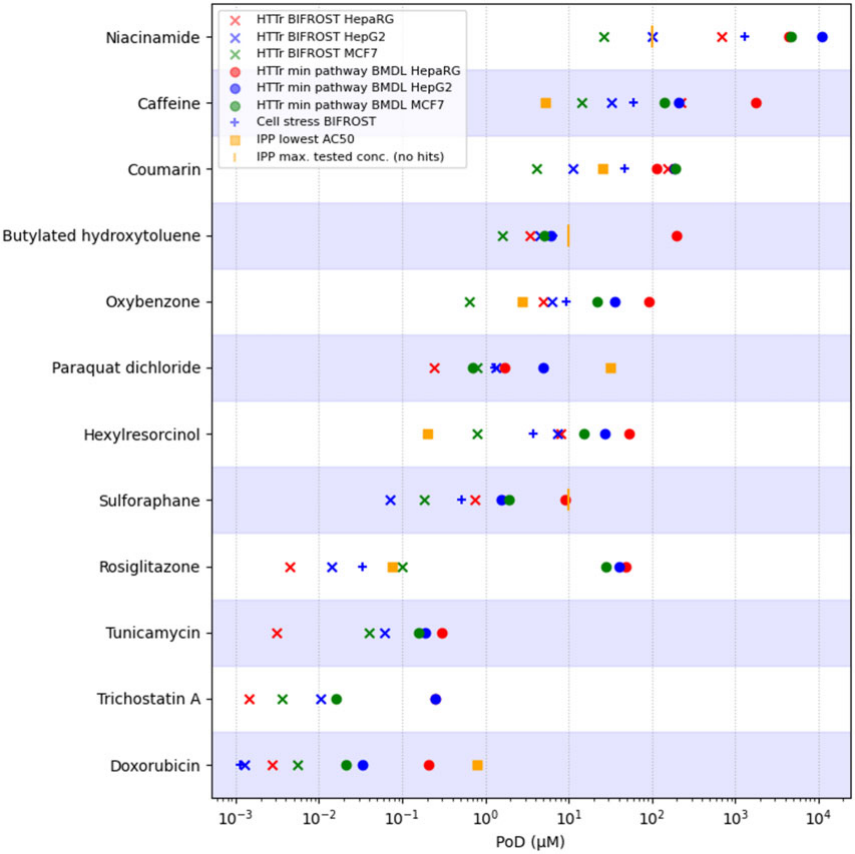
Overview of platform PODs (*in vitro* pharmacological profiling—IPP, cell stress panel, and high-throughput transcriptomics—HTTr) obtained using the toolbox for each of benchmark chemicals. High-throughput transcriptomics data were generated for 3 cell lines (MCF7, HepaRG, HepG2) and analyzed using 2 different methods (BMDexpress and BIFROST), resulting 6 transcriptomics platform PODs per chemical. Positive controls for the transcriptomics platform (Tunicamycin and Trichostatin A) are also included.

An important consideration within the evaluation (Figure 2) is the biological coverage of the toolbox bioactivity platforms and understanding whether the associated PODs are sufficiently protective. Thus, in addition to generating exposure estimates for each exposure scenario in the evaluation, at stage 2 (Figure 2) corresponding concentration-response data were also generated using the 3 toolbox bioactivity platforms (cell stress panel, *in vitro* pharmacological profiling, and high-throughput transcriptomics). Typically, studies investigating the bioactivity of test chemicals in *in vitro* test systems do not take into consideration the physicochemical properties of the test chemicals and the possibility that they may be difficult to test within assay conditions, ie, due to occurrence of plastic or protein binding, for example. This was a potential concern for 7 of the test chemicals (butylated hydroxytoluene, coumarin, doxorubicin, oxybenzone, paraquat dichloride, sulforaphane, and valproic acid), and therefore a separate dose-confirmation study was performed in parallel to determine whether for those test chemical, the expected nominal concentrations could be achieved under assay conditions (see Supplementary Information S1, F2). Of those investigated, valproic acid was the only chemical where all measured concentrations differed from nominal by more than 2-fold, indicating potential systematic issues with the testing of this particular test substance within the toolbox bioactivity platforms. Because of this, the bioactivity data for valproic acid were not considered further. For all other test chemicals, individual PODs were obtained for each biomarker (gene probe count, glutathione content level, etc.) within a bioactivity platform, resulting in platform PODs for 10 benchmark chemicals (Figure 4 and Supplementary Information S1, T6).

Estimation of PODs from high-throughput transcriptomics data is an active area of research and there is considerable debate about the selection of which method or POD definition is most appropriate for NGRA (Baltazar *et al.*, 2020; Farmahin *et al.*, 2017; Harrill *et al.*, 2019, 2021; Reynolds *et al.*, 2020). To begin to explore the potential impact of selecting one approach over another, the transcriptomics data were analyzed using 2 different methods, BMDExpress2 and BIFROST, resulting in 2 different POD estimates per transcriptomics dataset. Importantly, the interpretation of the PODs generated by the 2 methods are different: the global PODs generated by BIFROST represents the minimum effect concentration based on individual gene expression changes (in response to the chemical treatment), whereas the BMDExpress2 BMDL PODs represent the lowest concentration at which mechanistic changes occur, inferred by Reactome pathways, and an estimate of apical endpoints (Farmahin *et al.*, 2017).

Overall, the various platform PODs varied across datasets by 6 orders of magnitude, from nanomolar to millimolar concentrations. The most potent chemical was doxorubicin (a chemotherapy drug), with platform PODs starting at ∼1 nM (obtained for both the cell stress and HepG2-transcriptomics data), whereas the least potent chemical was niacinamide (which is typically used as an ingredient in consumer products), with platform PODs starting at ∼26 µM (obtained for the HepG2 and MCF-7 transcriptomics data). For most chemicals (7 out of 10), the smallest PODs tended to come from the transcriptomics platform when analyzed using BIFROST, except caffeine and hexylresorcinol (the smallest PODs were obtained using the *in vitro* pharmacological profiling platform) and doxorubicin (obtained using cell stress panel). The BMDL-based transcriptomics platform PODs obtained using BMDExpress2 tended to be several orders of magnitude larger than the global PODs obtained using BIFROST (due to averaging over several gene level BMDLs), reflecting the fact that higher concentrations are needed to trigger coordinated biological pathway responses. Overall, these results were as expected, because the transcriptomics platform had been included in the toolbox to provide a broad coverage of effects that may not be detected using *in vitro* pharmacological profiling and the cell stress platform and so was expected to lead to more conservative (and therefore protective) POD estimates, at least when considering the transcriptomics global PODs obtained using BIFROST.

To understand the extent to which the biological perturbations observed using *in vitro* pharmacological profiling and the cell stress platforms were reflective of the assays capturing known biological mechanisms, a comparison between the observed responses from the 2 platforms and the known biological effects was performed for all 10 chemicals (see Supplementary Information S1, sections T7, F3, and F4 and Supplementary Information S2).

For most of the chemicals (9/10), the cell stress and *in vitro* pharmacological profiling platforms provided coverage of at least one of their known modes of action (pharmacological or toxicological). For *in vitro* pharmacological profiling, the lowest global PODs for hexylresorcinol, caffeine, and rosiglitazone were all associated with known biological targets of those chemicals (cyclooxygenase inhibition, adenosine 2A antagonism, and PPAR-gamma agonism, respectively). Other specific targets were also identified for other chemicals (paraquat, coumarin, and doxorubicin), even though these were not among the lowest effects detected. In particular, this was only possible for coumarin and paraquat because the initial screening concentration for the *in vitro* pharmacological profiling had been modified from the standard 10 µM to a higher concentration of 100 µM, to take into consideration chemicals with relatively low potency in the assay. Using a lower screening concentration would have resulted in these effects being missed. However, not all known targets for every chemical were detected using the panel: for example, carbonic anhydrase II effects have been reported elsewhere for coumarin (Maresca *et al.*, 2009; Maresca and Supuran, 2010), but were not detected in this work.

The cell stress panel was designed to characterize 10 major pathways (including mitochondrial toxicity and oxidative stress) involved in homeostatic processes and was shown to correctly identify chemicals which perturb 1 or more of these pathways (Hatherell *et al.*, 2020). Across the chemicals tested in this work, various known cell stress effects were detected using the panel. For example, sulforaphane is a soft electrophile that is a common component of various foods and has widely been hypothesized to exert antioxidant effects through upregulation of NRF2. Consistent with this, sulforaphane was found to cause an upregulation of GSH content and oxidative stress at subcytotoxic concentrations. Similarly, subcytotoxic effects on mitochondrial respiration (ie, measured using the extracellular flux assay) were observed for chemicals associated with mitochondrial toxicity such as rosiglitazone (Hu *et al.*, 2015) and paraquat (Baltazar *et al.*, 2014; Huang *et al.*, 2016).

The cell stress panel was only recently developed, and so additional attention was given to investigating reproducibility of the panel when generated at the same laboratory in 2 independent studies. Cell stress panel data for 6 of the chemicals used in the (Hatherell *et al.*, 2020) study (coumarin, caffeine, doxorubicin, niacinamide, rosiglitazone, and sulforaphane) were therefore repeated a second time in this study. Overall, results were comparable. In particular, the global POD generated via the BIFROST method (rather than the PODs of individual biomarkers) is a key output of the cell stress panel because it is used to estimate the BER. A comparison between this for each of the repeated chemicals is provided in Supplementary Information S1, F5. PODs for the higher potency chemicals (doxorubicin, rosiglitazone, and sulforaphane) were within 2-fold of each other. Global PODs for the lower potency chemicals (caffeine, coumarin, and niacinamide) were less concordant, ranging from 3.5 to 12-fold apart, and were lower than those obtained in Hatherell *et al.* (2020). The reasons for this are 2-fold: (1) the modification of the experimental design because the work reported in Hatherell *et al.* (2020) to correct for plate effects (see Materials and Methods) has resulted in increased sensitivity to detect weaker magnitude responses and (2) testing to higher concentrations of chemicals compared with the initial work reported in Hatherell *et al.* (2020) allows for more confidence in hits because a larger response is generally observed at concentrations greater than the minimum effect concentration.

Together, these results indicated that although the cell stress and *in vitro* pharmacological profiling panels could be used to detect various known modes of toxicity, the transcriptomics platform data analyzed using BIFROST (compared with the PODs obtained using BMDExpress2) typically provided the most conservative POD estimate across the different *in vitro* assays.

#### Estimating the Bioactivity Exposure Ratio Using New Approach Methodologies

**Figure 5.**
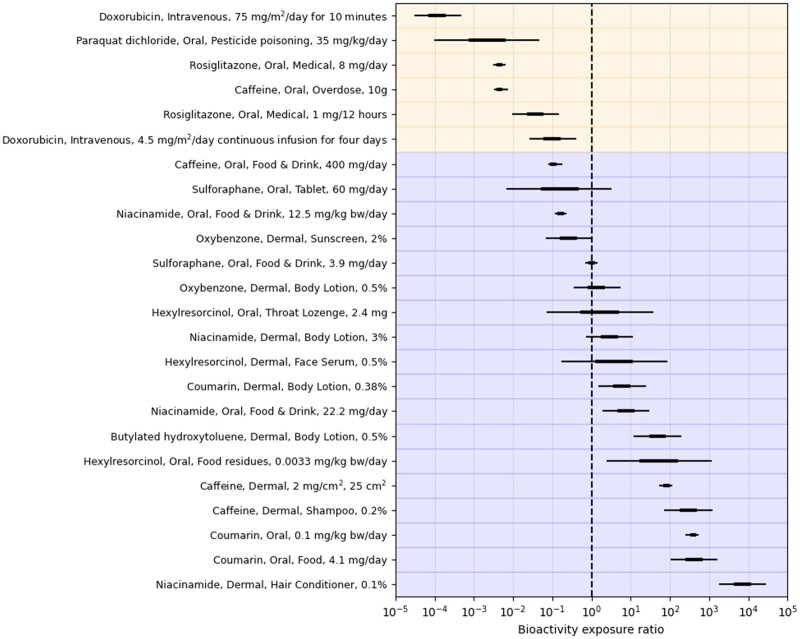
Centered 50% and 95% credible intervals summarizing the distribution of the bioactivity exposure ratio (BER) when using all available predicted *C*_max_ estimates. Background colors indicate the assigned risk category for each benchmark chemical-exposure scenario assigned at stage 1 (blue—low, yellow—high). The vertical dashed line indicates a BER equal to 1.

At stage 3 of the evaluation (Figure 2), BER distributions were obtained for all the benchmark chemical-exposure scenarios, using the distributions from the *C*_max_ error distribution model and the minimum POD (see Materials and Methods). As such, the BER distributions provide a probabilistic description of what the true BER is, given the uncertainty in the PBK *C*_max_ estimates. As a first step in benchmarking, the toolbox data against historical safety decisions, the BER distributions were compared with the risk classifications assigned at stage 1 to each the benchmark chemical-exposure scenario (Figure 5). Here, exposure scenarios are ranked by the median estimated BER, from smallest to largest along the *y*-axis, and color-coded according to their assigned risk-categories (see Table 1). BER credible ranges are plotted along the *x*-axis (with the relative width of these ranges being driven by the *C*_max_ error distribution model and are the same as in Figure 3). Of note, a BER < 1 indicates the plasma *C*_max_ is above the minimum POD measured across the bioactivity platforms. Based on this ranking, the first 6 exposure scenarios in Figure 5 were all high-risk benchmark chemical-exposure scenario (see Table 1) and all have a median BER less than 1, whereas the last 13 were all low-risk benchmark chemical-exposure scenarios and have a median BER between 0.1 and 10,000. The expected correlation (point-biserial) between the BER and assigned benchmark risk categories (ie, obtained at stage 1) is −0.73 with a centered 95% interval from −0.77 to −0.67 (here, the correlation is negative indicating high-risk exposures are associated with lower BERs). Overall, the BERs for some low risk scenarios (eg, ones associated with caffeine, sulforaphane, niacinamide, or oxybenzone) overlap with BERs associated with high-risk scenarios (eg, from exposure to the drugs rosiglitazone and doxorubicin). Overall, this was as expected, because not all *in vivo* bioactivity will necessarily lead to adverse effects. For example, the POD driving the BER for caffeine corresponds to the adenosine A2A receptor, its main target, and so the caffeine oral drink exposure (which was classified as low risk) is expected to have a BER < 1, reflecting the fact that there is an activity threshold below which A2A receptor activity is not expected to cause adverse health effects.

#### Decision Models and Quantifying Protectiveness and Utility

Overall, the results presented in Figure 5 indicated that the BER obtained from the toolbox may be used to correctly identify many of the low-risk benchmark chemical-exposure scenarios whilst being protective against the high-risk ones, provided a suitable decision model was used. The final stage of the evaluation approach involves assessing the utility and protectiveness of the toolbox for a given decision model. The utility reflects the proportion of low-risk benchmark chemical scenarios correctly identified as such using the toolbox data and decision model (see below), whereas the protectiveness is the proportion of high-risk scenarios not identified as low risk (and are instead identified as uncertain risk).

The decision model used in this work is based on the idea of setting a threshold value on the BER, such that chemical-exposure scenarios with a true BER value >1 (ie, Prob.(BER > 1)) are identified as low risk (ie, reflecting the case where *C*_max_ is below the minimum POD), whereas exposure scenarios with a true BER < 1 are identified as uncertain risk (ie, could in reality be either low or high risk). To understand how this decision model could be implemented with the BER distributions, which provide a probabilistic description of what the true BER value is, given the uncertainty in the *C*_max_ PBK estimates, BER distributions were generated for each exposure scenario and PBK level (L1–L3). Using these, Prob.(BER > 1) was then calculated for each chemical-exposure scenario. Examples based on 3 of the caffeine benchmark exposure scenarios are given in Table 2 (see Supplementary Information, Section M4 for the results for all 24 benchmark chemical-exposure scenarios). Here, it can be seen that Prob.(BER > 1) is close to 1 for the low-risk caffeine shampoo (0.2%) dermal exposure scenario (Table 2, rows 1–3) at all 3 PBK levels (L1–L3). Conversely, for the high-risk caffeine oral overdose exposure scenario (Table 2, rows 7–9), Prob.(BER > 1) is close to zero for all 3 PBK levels.

**Table 2.** Probability That the BER >1 for 3 Exposure Scenarios for Caffeine for All 3 PBK Parameterization Levels

Chemical	Route	Exposure	Level	Risk	Prob. BER > 1	BER 2.5th Quantile	BER 50th Quantile	BER 97.5th Quantile
Caffeine	Dermal	Shampoo, 0.2%	L1	Low	1.00	17	1700	180 000
Caffeine	Dermal	Shampoo, 0.2%	L2	Low	1.00	8.6	200	4400
Caffeine	Dermal	Shampoo, 0.2%	L3	Low	1.00	80	290	1100
Caffeine	Oral	Food and drink, 400 mg/day	L1	Low	.42	0.0057	0.63	63
Caffeine	Oral	Food and drink, 400 mg/day	L2	Low	.08	0.0050	0.11	2.6
Caffeine	Oral	Food and drink, 400 mg/day	L3	Low	.01	0.054	0.20	0.77
Caffeine	Oral	Overdose, 10 g	L1	High	.06	0.00022	0.024	2.5
Caffeine	Oral	Overdose, 10 g	L2	High	.00	0.00038	0.0083	0.19
Caffeine	Oral	Overdose, 10 g	L3	High	.00	0.0020	0.0080	0.032

In the “Prob. BER > 1” column, BER probabilities vary from 1 (indicating high certainty that the BER exceeds 1) to 0, indicating high certainty that they do not exceed 1. Probabilities close to .5, indicating high uncertainty with respect to which side of one the BER falls.

In general, Prob.(BER > 1) was small for all the high-risk benchmark chemical-exposure scenarios included in the study, at all PBK levels, indicating that using BER value of 1 may provide a high degree of protectiveness (ie, so that none of the high-risk benchmark exposure scenarios are identified as low risk). However, Prob.(BER > 1) was not large for all the low-risk benchmark scenarios, reflecting the fact that some of these may be identified as uncertain risk when using the toolbox. For example, the low-risk caffeine food and drink exposure scenario (Table 2, rows 4–6), Prob.(BER > 1) was 0.42 when using the L1 prediction, reflecting the uncertainty of whether the true population-average *C*_max_ exceeds the minimum POD. However, at higher PBK levels, Prob.(BER > 1) was far lower at L2 and L3 (0.08 and 0.01, respectively), indicating increased certainty that the true population average *C*_max_ is greater than the minimum POD. As discussed above, this prediction is consistent with the mode of action of caffeine at normal levels of consumption.

Although the BER > 1 probabilities in Table 2 may be used by a risk assessor to guide the overall decision-making process, a threshold value on Prob.(BER >1) needs to be assigned to complete the decision model. This threshold on the probability, denoted *p*_threshold_, can be regarded as a measure of the confidence required to identify an exposure as low risk (ie, that BER >1), and as such we refer to it as a “confidence threshold” hereafter. To explore this systematically, empirical estimates of the protectiveness and utility of the toolbox were obtained for each PBK level (Supplementary Information S1, F6). For the benchmark exposure scenarios considered in this work, full protectiveness could be achieved at every PBK level if the decision was to accept a confidence threshold (*p*_threshold_) of as little as .25. Higher confidence thresholds maintain full protectiveness but cause a decrease in utility.

The fact that the *C*_max_ error distribution model essentially places a normal distribution about the base-10 logarithm of PBK *C*_max_ estimates leads to a useful mathematical property of the BER distributions. First, it means that BERs are also normally distributed on a log10 axis, so that the geometric mean of the distribution is equal to the BER point estimate (ie, the ratio between the PBK *C*_max_ estimate and the minimum POD). Second, it means that requiring that Prob.(BER > 1) > *p*_threshold_ is equivalent to requiring that the BER point estimate is above a certain threshold value. This threshold value is henceforth called the “threshold BER.” In general, the variance of a BER distributions depend on which PBK level the distribution corresponds to (see, eg, Supplementary Information S1, F7). This, in turn, means that the threshold BER will also vary by PBK level. For example, from a risk assessment perspective, a reasonable confidence threshold at each PBK level would be 95% (ie, *p*_threshold_ = .95) (see Supplementary Information S1, F7, red curves). At L1, this is equivalent to requiring that the BER point estimate exceeds a threshold BER of 35; at L2, that it exceeds a threshold BER of 7.1; at L3, that it exceeds a threshold BER of 2.5.

A potentially undesirable feature of any decision model is that if a “low risk” decision at PBK parameterization L1 is reclassified at L2 or L3, ie, as the PBK model is refined further through additional data. This could happen when *C*_max_ is underestimated at L1, causing it potentially to appear as low risk, but at higher PBK levels the estimate of *C*_max_ becomes more accurate, increasing to be closer to its true value, thereby leading to a change in the risk classification. To ensure that “low risk” decisions are unlikely to change when moving from 1 PBK level to the next, more stringent confidence thresholds (or, equivalently, threshold BER values) need to be used at lower PBK levels than at higher ones. Intuitively, this is because lower PBK levels use more uncertain forms of parameterization (eg, *in silico* versus *in vitro* data sources).

The confidence thresholds required at each PBK level to ensure that the probability of overturning a “low risk” decision is no more than .1 was calculated (see Supplementary Information S1, M5 for full details of the calculation). The confidence thresholds were calculated by starting at the highest PBK level (L3) and setting the confidence threshold needed to determine whether an exposure is low risk. Here, we chose *p*_threshold_ = .95 (as above), ie, 95% of the BER distribution for a given exposure needs to be greater than 1 to be considered low risk. Next, the confidence threshold needed at L2 was calculated. The variance of the BER distributions at L2 is greater than at L3, and therefore a larger confidence threshold (*p*_threshold_ = .97) is required to ensure low risk decisions at L2 are retained with high probability at L3. In other words, a greater proportion of the BER distribution needs to be >1 at L2 (97%) than at L3 (95%) due to the lower accuracy of L2 PBK models. Similarly, at L1, which has an even higher variance in the BER distribution, the confidence threshold needs to be *p*_threshold_ = .98.

Finally, requiring 95% (ie, *p*_threshold_ = .95) of the L3 BER distribution >1 is equivalent to a threshold BER of 2.5 (blue curve, Supplementary Information S1, F7), as before. Similarly, the 97% requirement (*p*_threshold_ = .97) at L2 is equivalent to requiring a threshold BER of 11 (blue curve, Supplementary Information S1, F7); at L1, the 98% requirement (*p*_threshold_ = .98) corresponds to a threshold BER of 110 (blue curve, Supplementary Information S1, F7). A summary of the resulting decision model is provided in Table 3.

**Table 3.** Summary of Prototype Decision Model

PBK Level	Threshold BER Required for Exposure to Be Identified as Low Risk	Confidence Threshold (*p*_threshold_) Required for Exposure Scenario to Be Identified as Low Risk	Probability of Overturning Low-Risk Decision at Next PBK Level	Empirical Utility	Empirical Protectiveness
1	110	.98	.1	3/18 (17%)	6/6 (100%)
2	11	.97	.1	6/18 (33%)	6/6 (100%)
3	2.5	.95	—	9/13 (69%)	5/5 (100%)

Based on this analysis, the prototype decision model was defined as follows:

1. Conclude low risk at PBK L1 if the BER point estimate >110; ie, equivalently if the probability that BER >1 exceeds the confidence threshold (*p*_threshold_) of .98.
2. Conclude low risk at PBK L2 if the BER point estimate >11; ie, equivalently if the probability that BER >1 exceeds the confidence threshold (*p*_threshold_) of .97.
3. Conclude low risk at PBK L3 if the BER point estimate >2.5; ie, equivalently if the probability that BER >1 exceeds the confidence threshold (*p*_threshold_) of .95.

**Figure 6.**
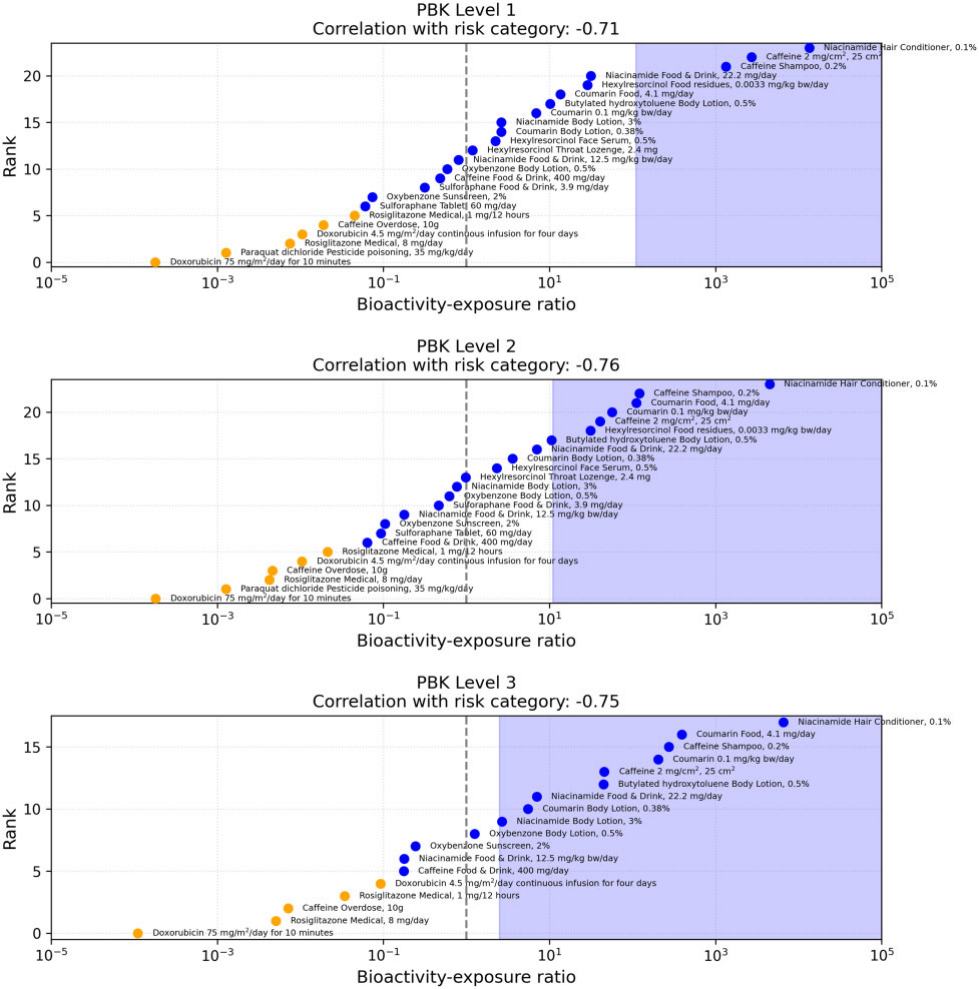
Chemical-exposure scenarios with a BER point estimate outside the blue-shaded region would be identified as “uncertain” risk under this decision model. The gray-dashed line corresponds to BER = 1.

The outcome of applying this decision model to the toolbox data for the 24 benchmark chemical-exposure scenarios is illustrated in Figure 6. Exposure scenarios within the blue-shaded region are identified as low risk, whereas ones outside this region are considered uncertain risk. The empirical utility and protectiveness conditional on this decision model is reported in Table 3. Although utility of the toolbox increases with the PBK level being used, it can be seen that the requirement of consistent decision-making means that many of the low-risk exposures will be identified as uncertain risk at L1 and L2 (low risk exposures outside of the blue region in Figure 6).

Basing the decision criteria on the BER point estimates provides a more pragmatic way to determine whether an exposure is low risk or not, as it no longer requires information about the BER distribution which was obtained using the (Bayesian) *C*_max_ error distribution model. Overall, the threshold BER values can be viewed as equivalent to traditional safety factors used in risk assessment, although the key difference is that they were derived in a data-driven manner using the toolbox.

#### Impact of Choosing Different Bioactivity Platforms on Toolbox Performance

To explore the relative contribution of the different bioactivity platforms on the toolbox and workflow outputs, the BER distributions (and associated protectiveness and utility metrics) were recalculated using various platform POD subsets, as summarized in Supplementary Information S1, T8.

Basing the BER on *in vitro* pharmacological profiling PODs alone did not offer full protectiveness across all PBK levels. Similarly, BMDexpress-derived PODs from transcriptomics data also failed to protect against at least 1 high-risk exposure for every combination of cell type and PBK parameterization level. Only PODs obtained with the BIFROST method (used to analyze the transcriptomics and cell stress panel data) offered full protectiveness against all the high-risk benchmark exposure scenarios. Maximal utility and protectiveness (of 92% and 100%, respectively) was attained (at PBK parameterization L3) when basing the BER either on only the cell stress panel or only the HepaRG transcriptomics data.

The size of the data being used in this analysis is relatively small, and so it is not possible to determine which combination of bioactivity platforms provides optimal levels of protectiveness and utility. However, this analysis illustrates how the protectiveness and utility metrics can be used to explore the relative impact of choosing 1 set of bioactivity platforms over another (ie, for inclusion in the toolbox) in terms of resulting safety decisions.

## DISCUSSION

Within NGRA, there is an ongoing need to determine whether NAMs can be used to make safety decisions that are protective of human health. Recently, we demonstrated how this could be done using a hypothetical case study in which coumarin was used as an ingredient in various consumer products (Baltazar *et al.*, 2020). A key aspect of that work was that a BER estimate (or margin of safety) obtained using NAMs (*in vitro* assays, PBK models, etc.) was combined with other toxicity data (eg, *in silico* predictions) to make safety decisions. However, it remains unclear the extent to which the tools and approaches used in that work could be applied more generally to assure systemic safety for wider range of chemical-exposure scenarios. At present, approaches designed to build confidence in NAMs are typically focused on hazard identification rather than their use in risk assessment, and in many cases involve validating the *in vitro* results against animal endpoint data. Moreover, in line with the principles of NGRA (Dent *et al.*, 2018), a key objective is to be protective of human health, rather than necessarily predictive of various adverse effects in animals. This distinction is particularly important in the context of systemic toxicity, where a broad range of potential adverse outcomes must be covered, many of which are often not fully characterized in terms of mechanism of action or adverse outcome pathways.

To address the above considerations, we propose here a systemic safety toolbox and workflow (Figure 1) based on the early-tier assays and models used in the (Baltazar *et al.*, 2020) study, together with an approach for evaluating how protective and useful it is (Figure 2). A key principle of the approach is benchmarking NAM data against existent safety decisions made using traditional methods, where the various tools (*in silico* and *in vitro*) are used together and assessed in the context of their intended use, ie, risk assessment. As such, this work provides an important basis for a full evaluation, to be conducted subsequently.

**Figure 7.**
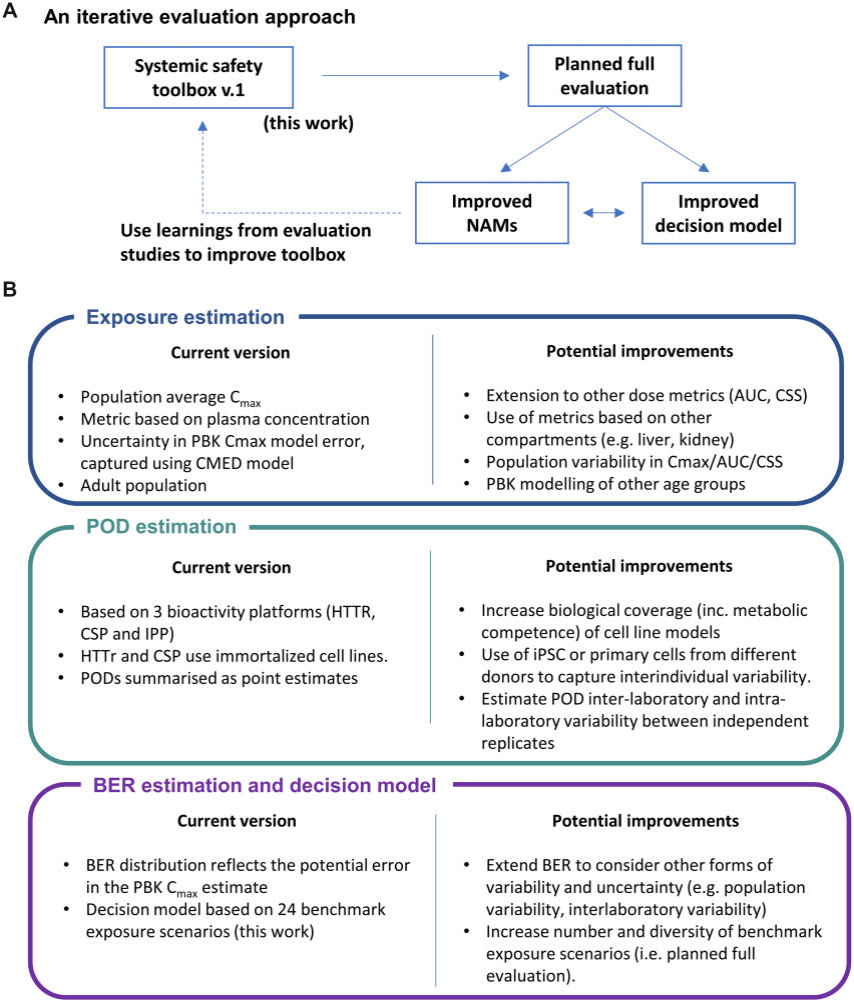
A, Summary of the iterative approach for evaluating and then refining the toolbox beyond the current version (ie, version 1). B, Overview of current toolbox version and potential improvements for future iterations. Abbreviations: iPSC, induced pluripotent stem cell; AUC, area under curve; CSS, steady-state concentration; BER, bioactivity exposure ratio; CMED, *C*_max_ error distribution; PBK, physiologically based kinetic; HTTr, high-throughput transcriptomics; CSP, cell stress panel; IPP, *in vitro* pharmacological profiling; POD, point of departure.

As a first step in establishing a toolbox for assessing a systemic safety, various pragmatic choices were made in its composition. Potential limitations include: (1) the biological coverage of the cell models and assays used in the 3 bioactivity platforms (including the metabolic competence of the cells); (2) PODs were estimated in terms of nominal concentration, and aspects important to *in vitro* to *in vivo* extrapolation, eg, the free concentration, were not explicitly considered. The dosing issues we found with valproic acid indicate that, for certain chemicals, it may be desirable to monitor exposures within the same *in vitro* systems used to generate PODs; (3) *C*_max_ was used as a metric to quantify exposure levels, which may not always be appropriate (eg, in cases when the exposure duration is important, in which case other metrics such as area under the curve or the steady-state concentration should also be considered); (4) population variability, either in terms of interindividual toxicokinetics or toxicodynamics (ie, PODs), which may be important when considering sensitive subpopulations or age groups other than adult, were not considered; (5) the concentration-response data were generated at a single timepoint (24 h). However, the overall concept of this work is to test the hypothesis that the toolbox and associated workflow is sufficiently protective. As such, testing, for example, the biological coverage of the cell assays and the choice of the 24-h timepoint and use of population average *C*_max_, is part of the evaluation. Furthermore, the learnings from the full evaluation can be used to identify which of these limitations are critical to the overall protectiveness and utility of the toolbox, so that improvements are introduced in an iterative manner (see Figure 7A). A summary of the current version of the toolbox and potential improvements is provided in Figure 7B. Finally, the results presented in Supplementary Information S1, T8 illustrate how the utility and protectiveness metrics can be used to assess the relative impact on safety decision-making of using 1 NAM over another, something that until now has been lacking.

Overall, depending on the PBK model parameterization level used to estimate *C*_max_, up to 69% of the low-risk benchmark exposure scenarios can be identified as such with the toolbox using the decision model defined in Table 3, whilst being protective against all the high-risk ones. The decision model is based on the probability of whether the BER exceeds 1, with the distribution reflecting uncertainty in the PBK estimates (quantified using the *C*_max_ error distribution model). This approach is reasonable, provided the PODs obtained from the bioactivity platforms are sufficiently protective and *C*_max_ is a conservative representation of the exposure. We demonstrated how the different decision points could either be based on different confidence threshold on whether the BER exceeds 1, or equivalently on different safety thresholds of the BER point estimate (see Table 3). This latter set of thresholds are analogous to traditional safety factors, except they were derived using a Bayesian statistical model, rather than being based on historical precedent and experience (Renwick, 1993). Importantly, from a decision-making perspective, only the PBK *C*_max_ estimate and minimum POD are required to determine whether an exposure is low risk or not, forgoing the need to explicitly consider the Bayesian statistical model, thereby vastly simplifying the approach. However, it should be stressed that the decision model is specific to the toolbox as described in Figure 1, including the *in vitro* cell models and assays, the computational models used to analyze the data or generate the exposure estimates. If for example the high-throughput transcriptomics data were analyzed only using BMDexpress, and not BIFROST, the decision model would need to be altered accordingly. Furthermore, the *C*_max_ error distribution model presented in the work was evaluated against 11 chemical-exposure scenarios where measured *C*_max_ was available, and it will be important to assess how well the model performs using a wider range of chemicals and exposure routes. In general, the use of Bayesian statistical models in toxicology have been explored in a variety of contexts (see, eg, Chiu *et al.*, 2017; Lazic *et al.*, 2018; Maertens *et al.*, 2022; Reynolds *et al.*, 2019, 2020; Wambaugh *et al.*, 2019; Williams *et al.*, 2020) and could be readily extended to capture other sources of uncertainty associated with the toolbox (eg, variability between replicates of an *in vitro* experiment conducted in separate labs or on different occasions, etc.), provided the necessary experimental data are available.

Several of the low-risk benchmark exposures could only be identified as uncertain risk using the toolbox, reflecting potential for the relevant chemicals triggering bioactivity at those exposures. For example, caffeine intake from the normal consumption of beverages (up to 400 mg caffeine/day) was predicted to be bioactive (BER < 1) and therefore was not identified as low risk using the toolbox. This is perhaps not surprising, given that caffeine is a psychoactive drug, and normal consumption of caffeinated drinks does lead to systemic bioactivity. When first assigning benchmark risk categories, we categorized this exposure as “low risk,” because although some individuals are sensitive to low levels of caffeine, 50 mg is widely considered safe for the general adult population without the need to apply any risk-benefit considerations. For this reason, it was challenging to assign an appropriate risk category to this chemical-exposure scenario. First, this highlights importance of capturing population variability (both in terms of biokinetics and biodynamics) in future iterations of the toolbox (ie, because, for some small fraction of the population, normal caffeine consumption could be considered “high risk” from a consumer goods perspective). One way to address this could be to use the approach set out in WHO (2018), which provide chemical-agnostic distributions that capture the uncertainty associated with interindividual differences in sensitivity across a population. These could be further refined using Bayesian models that quantify the population variability both in terms of PODs (eg, using primary cells or induced pluripotent stem cell models from multiple donors) (Blanchette *et al.*, 2022; Chiu *et al.*, 2017) or internal exposure levels (eg, using Bayesian PBK models) (Krauss *et al.*, 2013; Wambaugh *et al.*, 2019). Second, the prediction by the toolbox that normal caffeine consumption will result in systemic bioactivity reinforces that this toolbox needs to be used as part of a tiered approach to safety assessment. In practice, for chemical exposures identified as uncertain risk using the toolbox, the decision of whether or not to continue refining the risk assessment using higher tier tools may depend both on the biological effects detected using the toolbox (ie, whether the perturbation of a particular pathway could be indicative of an adverse effect or an adaptive response) and the size of the BER (contrast, eg, the median BER for the chemotherapy drug doxorubicin, which is ∼0.001, with the median BER for the normal consumption of caffeine, which is ∼0.1). Higher tier tools that could be used to investigate whether systemic bioactivity predicted by the toolbox could indeed result in adverse effects (either by refining the bioactivity characterization or the exposure estimates) include microphysiological systems (Ewart *et al.*, 2018; Peterson *et al.*, 2020), use of primary-derived cell models and other bespoke assays for investigating specific biological processes (eg, aligned to specific adverse outcome pathways [Carusi *et al.*, 2018; Villeneuve *et al.*, 2014]), including transport (Bajaj *et al.*, 2020) or metabolism of the test chemical (Miranda *et al.*, 2021).

The results obtained for the small set of benchmark chemical-exposure scenarios in Table 1 have given us sufficient confidence to move on to the full evaluation. The benchmark chemical-exposure scenarios to be used in the full evaluation must cover a wide range of different mechanisms, potencies, and exposure scenarios whilst limiting the appearance of extremes or biases in the final set of benchmarks. For example, if the evaluation consisted solely of either highly potent chemicals like doxorubicin, or those that are relatively inert like coumarin or niacinamide, one could obtain almost perfect separation between low-risk and high-risk exposures based on the BER, but this would not be representative of the wider chemical-exposure space that consumer safety occupies. Given the limitations discussed above, it is quite possible that “version 1” of the toolbox (as defined in this work), using the proposed decision model in Table 3, will not be protective for all the high-risk benchmark chemical-exposure scenarios that will be used in the full evaluation. Defining *a priori* the exact number of chemicals or exposure scenarios needed to complete the evaluation and finalize the decision model is currently not possible given the lack of available data. However, using the iterative approach defined above (see Figure 7A), the data generated in the full evaluation can be used to update and refine the decision model where necessary, provided whatever hypothesized improvements are then tested with another independent dataset. Through this process, it is anticipated that a “final” toolbox and decision model can be attained, although this may require several iterations.

Overall, the decision model summarized in Table 3 is significantly less stringent than that recently proposed by Health Canada. There, it was suggested that, for screening assessments conducted under the Canadian Environmental Protection Act, 1999, ToxCast data and high throughput toxicokinetics (see egPaul Friedman *et al.*, 2020; Pearce *et al.*, 2017, ) may be used as a line of evidence to support a decision of not toxic when the BER >1000 (Health Canada, 2021). The use of this relatively large BER may reflect the current lack of consensus on how to deal with uncertainty when using NAMs for decision-making. However, it is envisaged that the approach outlined in this work, based on the use of predefined benchmarks, data-driven hypothesis generation, and systematic testing, can help build such a consensus and ensure that NAMs can confidently be used to make safety decisions.

## CONCLUSIONS

A core toolbox of NAMs (*in vitro* and computational) and associated workflow was developed that, in an initial evaluation, can be used to provide BERs which appeared to enable protective systemic safety decisions to be made without using any animal data. This work will enable a full evaluation of the performance of the toolbox to assess its protectiveness and utility across a broader range of chemical-exposure scenarios. Furthermore, this pilot study has identified important limitations of the NAMs used, which can be addressed in future iterations of the toolbox.

## SUPPLEMENTARY DATA

Supplementary data are available at *Toxicological Sciences* online.

## DECLARATION OF CONFLICTING INTERESTS

The authors declared no potential conflicts of interest with respect to the research, authorship, and/or publication of this article.

## Supplementary Material

kfac068_Supplementary_Data
