# Supplementary material for: Are Non-animal Systemic Safety Assessments Protective? A Toolbox and Workflow
Source: Toxicol Sci. 2022 Jul 13;189(1):124–47. doi: 10.1093/toxsci/kfac068 (PMC9412174; doi:10.1093/toxsci/kfac068)
Supplement: kfac068_Supplementary_Data [file kfac068_supplementary_data.docx]

**Supplementary Information S1**

Are non-animal systemic safety assessments protective? A toolbox and workflow

Alistair M. Middleton^1^*, Joe Reynolds^1^, Sophie Cable^1^, Maria Teresa Baltazar^1^, Hequn Li^1^, Samantha Beven^2^, Paul L. Carmichael^1^, Matthew Philip Dent^1^, Sarah Hatherell^1^, Jade Houghton^1^, Predrag Kukic^1^, Mark Liddell^1^, Sophie Malcomber^1^, Beate Nicol^1^, Benjamin Park^2^, Hiral Patel^3^, Sharon Scott^1^, Chris Sparham^1^, Paul Walker^3^, Andrew White^1^

^1^Unilever Safety and Environmental Assurance Centre, Colworth Science Park, Sharnbrook, Bedfordshire, MK44 1LQ, United Kingdom

^2^Discovery Services, Charles River, Chesterford Research Park, CB10 1XL, United Kingdom

^3^Cyprotex Discovery Ltd, No. 24 Mereside, Alderley Park, Macclesfield, Cheshire, SK10 4TG, United Kingdom.

Contents

[Supplementary Modeling Information 3](#_Toc106373749)

[M1. Gastroplus modelling assumptions 3](#_Toc106373750)

[M2. Concentration-response analysis of cellular stress data 3](#_Toc106373751)

[M3. Concentration-response analysis of HTTr data using the BIFROST method 10](#_Toc106373752)

[M4. Probability of BER >1 16](#_Toc106373753)

[M5. Consistent Decision Making 19](#_Toc106373754)

[Distributions of BERs predicted by the CMED model 19](#_Toc106373755)

[Derivation of confidence thresholds to ensure low risk decisions are robust 20](#_Toc106373756)

[Thresholds for consistent decision making 22](#_Toc106373757)

[Supplementary Tables 24](#_Toc106373758)

[T1. Parameters for PBK Models 24](#_Toc106373759)

[T2. PBK Cmax estimates 34](#_Toc106373760)

[T3. Cell Stress Panel: Biomarkers and Reagents 35](#_Toc106373761)

[T4. Cell Stress Panel and HTTr dosing 38](#_Toc106373762)

[Cell Stress Panel 38](#_Toc106373763)

[HTTr 39](#_Toc106373764)

[T5. Cell information 40](#_Toc106373765)

[Cell Stress Panel 40](#_Toc106373766)

[HTTr cell lysate generation 41](#_Toc106373767)

[T6. Summary of the PODs estimated for each bioactivity platform 41](#_Toc106373768)

[T7. Summary of the bioactivity observed for each of the 11 chemicals and comparison to the known mechanism of action 42](#_Toc106373769)

[T8. Impact of choosing different bioactivity platforms on toolbox performance 43](#_Toc106373770)

[Supplementary Figures 44](#_Toc106373771)

[F1. Cmax Error Distribution model cross validation 44](#_Toc106373772)

[F2. Dose confirmation data 45](#_Toc106373773)

[F3. Cell stress 46](#_Toc106373774)

[Caffeine 46](#_Toc106373775)

[Coumarin 47](#_Toc106373776)

[Paraquat dichloride 48](#_Toc106373777)

[Butylated Hydroxytoluene (BHT) 49](#_Toc106373778)

[Oxybenzone 50](#_Toc106373779)

[4-Hexylresorcinol 51](#_Toc106373780)

[Sulforaphane 52](#_Toc106373781)

[Rosiglitazone 53](#_Toc106373782)

[Niacinamide 54](#_Toc106373783)

[Doxorubicin hydrochloride 55](#_Toc106373784)

[F4. IPP 56](#_Toc106373785)

[Caffeine 56](#_Toc106373786)

[Coumarin 56](#_Toc106373787)

[Doxorubicin Hydrochloride 57](#_Toc106373788)

[4-Hexylresorcinol 58](#_Toc106373789)

[Oxybenzone 61](#_Toc106373790)

[Paraquat 67](#_Toc106373791)

[Rosiglitazone 69](#_Toc106373792)

[F5. Cell Stress Panel Reproducibility 71](#_Toc106373793)

[F6. Utility and protectiveness of the toolbox for different confidence thresholds. 71](#_Toc106373794)

[F7. Toolbox bioactivity exposure ratio decision model. 72](#_Toc106373795)

[References 74](#_Toc106373796)

# Supplementary Modeling Information

## M1. Gastroplus modelling assumptions

Oral exposure predictions were obtained with a generic Advanced Compartmental And Transit (ACAT) model in GastroPlus. The ACAT model represents the physiology of the gastrointestinal tract by a series of nine compartments, each of which is divided into four subcompartments: unreleased, undissolved, dissolved, and enterocyte. The transit time and size of each compartment were related to the physiology being modelled. Each compartment was further characterized by pH, bile salt concentration, and surface enhancement factors, which take into account the increase in the available surface for absorption due to the presence of villi and microvilli. A detailed description of the model can be found in the GastroPlus manual (reference: Gastroplus manual, 2018. Version 9.6. May. Simulations Plus, Inc, Lancaster, CA). Briefly, the model assumes that the regional absorption rate coefficient for each gastrointestinal compartment is the product of the effective permeability Peff (representing an average permeability across the entire gastrointestinal tract) and an absorption scale factor (ASF) specific for each gastrointestinal compartment. An estimate of ASF for each compartment is obtained with Opt logD Model SA/V 6.1 based on input parameters LogP and pKa. The dissolution model used was the Johnson model (Lu et al., 1993) in which the particle radius was considered constant and set at default 25 um. Chemical specific parameters LogP, Pka, solubility and Peff as input were either determined in silico, in vitro or calibrated. All the other parameters were the default parameters of GastroPlus.

Dermal exposure predictions were obtained with a generic Transdermal Compartmental Absorption and Transit model (TCAT) model in GastroPlus. The GastroPlus TCAT dermal module is a complex mechanistic model of dermal absorption that predicts skin penetration from diffusion and partitioning coefficients of the chemical in various skin layers, i.e., the stratum corneum, epidermis and dermis. The dosing scenario is also required, including the dose, the site of application and its area. The dermal module has the capability to include formulation related parameters, such as partitioning between vehicle and water, evaporation, etc. A detailed description of the model can be found in the GastroPlus manual (reference: Gastroplus manual, 2018. Version 9.6. May. Simulations Plus, Inc, Lancaster, CA). Chemical specific parameters, i.e., diffusivity and partition coefficient of chemicals in vehicle and various skin layers, were either obtained from in silico QSAR models or were determined by fitting the model to the ex vivo skin penetration data, i.e. absorption into the receptor fluid or/and distribution in different skin layers (adjusting parameters until there is minimal difference between model output and the experimental data). The parameters determined in this fashion can then be used to inform the *in vivo* model of skin penetration. All the other parameters were the default parameters of GastroPlus.

## M2. Concentration-response analysis of cellular stress data

**Plate Design**

Cells are treated with the test substance(s) within a 384-well plate. The arrangement of treatments is illustrated in Figure 1 below. Briefly, all treatments for a single test substance are located within a single column of the plate (notation CX TY corresponds to chemical X, treatment concentration Y). There are eight distinct treatment concentrations, with two technical replicates each, located in adjacent rows. The highest treatment concentrations are located in rows A and B. Every third column on the plate contains DMSO controls only (notation DMSO). Up to two test substances used as positive controls may be present on the plate (notation PCX TY). If present, they are located in columns 3 and 4. For each assay, three biological replicates are prepared, with each biological replicate using a distinct plate.


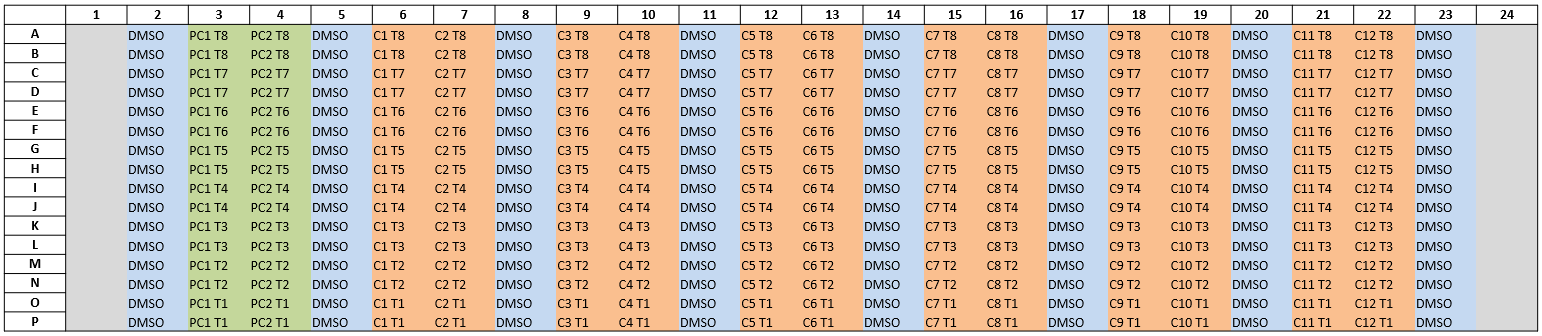


Figure 1 Schematic of the 384-well plate design. Wells in blue columns contain DMSO controls. Green columns contain the positive control chemicals used by Cyprotex. Each orange column contains one of the 12 treatment chemicals of interest. The highest treatment concentrations are in rows A and B, and lowest in rows O and P.

**Data Normalisation**

Data are normalised prior to concentration-response analysis. Denote by $y_{i,j}^{\text{raw}}$ the raw measurement on row $i$ in column $j$ if the position corresponds to a treatment with a test substance (or positive control) or denote by $z_{i,j}^{\text{raw}}$ if the position corresponds to a DMSO control. Raw values first undergo a robust logarithmic z-transformation. Let $\bar{z}^{\text{raw}}=\text{median}_{i,j}\left(z_{i,j}^{\text{raw}}\right)$ be the median of the raw DMSO control measurements. We first calculate the base-2 log fold change from the average of the DMSO control as

$$y_{i,j}^{\text{log}}=\log_{2}\left(\frac{y_{i,j}^{\text{raw}}}{\bar{z}^{\text{raw}}}\right) , z_{i,j}^{\text{log}}=\log_{2}\left(\frac{z_{i,j}^{\text{raw}}}{\bar{z}^{\text{raw}}}\right) .$$

Next, we calculate the median absolute deviation of this quantity as

$$\text{MAD}_{z}^{\log}=\text{median}_{i,j}\left(\left|z_{i,j}^{\log}-\text{median}_{i,j}\left(z_{i,j}^{\log}\right)\right|\right)$$

which is then used to rescale the log fold change to

$$y_{i,j}^{\log\text{scaled}}=\frac{y_{i,j}^{\log}}{\text{MAD}_{z}^{\log}} , z_{i,j}^{\text{log} \text{scaled}}=\frac{z_{i,j}^{\log}}{\text{MAD}_{z}^{\log}} .$$

The log fold change may exhibit considerable drift across a plate, as illustrated by the example in Figure 2 (top left). A second normalisation step attempts to correct for the drift by first fitting a Gaussian process (GP) plane to the transformed DMSO control measurements followed by extrapolating this plane to all wells on the plate.

Let the vector $z^{\text{log} \text{scaled}}$ contain all normalised control responses $z_{i,j}^{\text{log} \text{scaled}}$ such that $\left|z_{i,j}^{\text{log} \text{scaled}}\right|<6$. Requiring the absolute value to be less than six removes the most extreme outliers from the normalisation step. We define a likelihood

where $\mathbf{0}$ is the zero-vector, is the identity matrix and the elements of the covariance matrix $K$ are defined as

$$K\left(u_{i,j},v_{i,j};\eta,\rho\right)=\left\{\begin{matrix} \eta^{2} & \text{if} i=j , \\ \eta^{2}\exp\left(-\frac{(u_{i}-v_{i})^{2}+(u_{j}-v_{j})^{2}}{\rho^{2}}\right) & \text{if }i\neq j . \end{matrix}\right.$$

An optimisation algorithm (the *minimize* function provided in the SciPy library for Python) is used to find $\hat{\eta}$, $\hat{\rho}$ and $\hat{\sigma}$ which maximise this likelihood. Conditional on the optimal values of these parameters, normalised responses are calculated as

To see the effect this normalisation step may have, see the example in Figure 2.


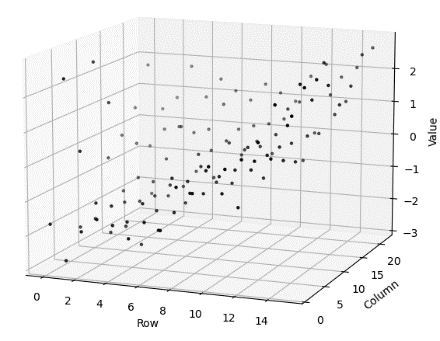

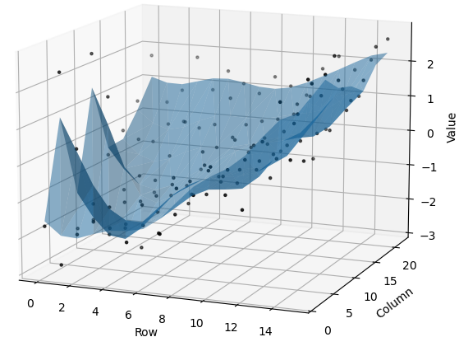

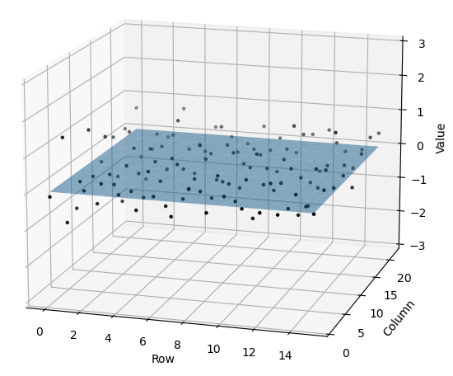


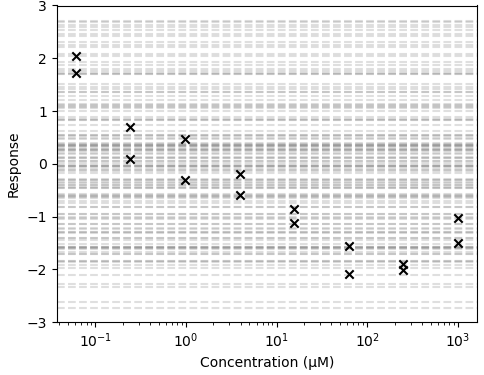

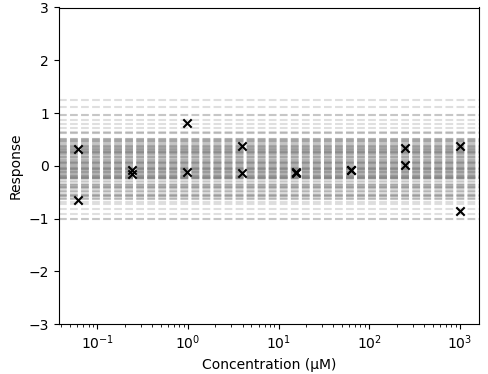


Figure 2 Top left: Example of drift in the response in solvent control samples across the plate. Top middle: Gaussian process plane fit to the response and used to interpolate the expected response in non-DMSO control well positions. Top right: normalised control data obtained by subtracting off the Gaussian process plane. Bottom left: response for one of the test substances (crosses) and DMSO controls (horizontal lines) on the plate prior to GP normalisation. Bottom right: the same data points as seen in the bottom left plot post GP normalisation.

**Concentration Response Modelling**

***Bayesian inference***

Normalised concentration-response data are analysed using a Bayesian statistical model. An early version of this model was first published in (Hatherell et al., 2020) and later a modified version for analysis of high-throughput transcriptomics concentration-response data was published in (Reynolds et al., 2020). Briefly, the approach attempts to describe all components of the variance in concentration-response data within a single, multi-level model. The relationship between concentration of the test substance and the expected response is modelled non-parametrically using a Gaussian process with a bespoke kernel designed for concentration-response datasets. The published model has been modified to the specific plate design used within this series of experiment. Indeed, the plate design was chosen with the downstream modelling in mind to ensure identifiability of parameters describing important components of the variance in the data.

Bayesian inference proceeds by first specifying a model of the data, , under consideration. In the Bayesian framework, a model is a multivariate[[1]](#footnote-2) probability distribution is assumed to be an adequate approximation of the sampling distribution of the data , conditional on a set of model parameters $\theta$. The sampling distribution of the data, conditional on model parameters $\theta$, is assumed to have the density . The specification of prior distribution on the set of model parameters, denoted $p\left(\theta\right)$, allows us to specify, a priori, a plausible range of values for the set of model parameters. The prior can be regarded as a set of assumptions used to regularise parameter estimates. Of interest in Bayesian inference is the posterior probability distribution which is defined in terms of the model, prior and distribution of data using Bayes theorem, which takes the form

.

The posterior distribution describes the joint distribution of model parameters compatible with both the data and the prior distribution. This distribution encodes the variance of each parameter estimate, which is regarded as a measure of the precision to which we can estimate the parameter using the data. For the concentration-response modelling discussed here, the posterior distribution of the model parameters informs a distribution over the expected response corresponding to a concentration of test substance less than the maximum concentration tested. This distribution allows one to calculate points of departure for use in NGRA.

***Concentration-response model***

To specify the multi-levelled nature of the model and data, the following notation is used. Subscripts $i$ are used to denote distinct treatment concentrations, subscripts $j$ are used to denote the plate on which an observation is made (biological replicates) and subscripts $k$ are used to denote repeat observations of the same treatment within a plate (technical replicates).

Let $y_{i,j,k}$ denote the *k*^th^ within-plate replicate of the normalised response to treatment with a single test substance at treatment concentration $i$, on plate $j$. Data for each test substance on a plate are analysed independently, however, each analysis shares the same DMSO control data. The sampling distribution for the normalised treatment response is modelled as a Student’s t-distribution

$$y_{i,j,k}\sim t\left(\nu,y_{i,j},\sigma\right)$$

with degrees of freedom $\nu$, scale $\sigma$ and location $y_{i,j}$, which corresponds to the latent median normalised response under treatment $i$ on plate $j$. The degrees of freedom parameter is given the weakly informative prior distribution

$$\nu\sim\text{Gamma}\left(2,0.1\right)$$

as recommended by Juárez and Steel (Juárez & Steel, 2010), whilst the scale parameter $\sigma$ is assigned the prior

$\sigma\sim\text{HalfNormal}\left(0,1\right)$.

Denote by $\mathbf{y}_{j}$ a vector whose elements are $y_{i,j}$ - that is, the vector of all distinct treatment responses for a single test substance on plate $j$. The sampling distribution for this vector is modelled as

$$\mathbf{y}_{j}\sim\text{MultivariateNormal}\left(\mathbf{0},K(\mathbf{x},\mathbf{x})\right)$$

where $\mathbf{x}$ is the vector of base-10 logged treatment concentrations corresponding to each element of $\mathbf{y}_{j}$ and $\mathbf{0}$ is a vector of zeros. The same sampling distribution is assumed for the response on all plates. The covariance matrix $K$ is defined in terms of its elements

$$\begin{matrix} K(x_{i},x_{j})=\left\{\begin{matrix} \alpha^{2}\text{Corr}(x_{i},x_{j},\rho,\theta) & \text{if} x_{i}\leq\theta\text{and} x_{j}\leq\theta, \\ \alpha\left(\alpha+\eta\left(x_{i}-\theta\right)^{2}\right)\text{Corr}(x_{i},x_{j},\rho,\theta) & \text{if} x_{i}>\theta\text{and} x_{j}\leq\theta, \\ \alpha\left(\alpha+\eta\left(x_{j}-\theta\right)^{2}\right)\text{Corr}(x_{i},x_{j},\rho,\theta) & \text{if} x_{i}\leq\theta\text{and} x_{j}>\theta, \\ \left(\alpha+\eta\left(x_{i}-\theta\right)^{2}\right)\left(\alpha+\eta\left(x_{j}-\theta\right)^{2}\right)\text{Corr}(x_{i},x_{j},\rho,\theta) & \text{if }x_{i}>\theta\text{and} x_{j}>\theta, \end{matrix}\right. \\ \text{where Corr}(x_{i},x_{j},\rho,\theta)=\exp\left(-\frac{\left(x_{i}-x_{j}\right)^{2}}{\rho^{2}\left(1+\left(x_{i}-\theta\right)^{2}\right)\left(1+\left(x_{j}-\theta\right)^{2}\right)}\right) . \end{matrix}$$

The covariance structure induces the property that the variance in the normalised response across plates increases for concentrations above some threshold concentration $\theta$. The magnitude parameter $\eta$ controls the rate at which the variance increases with respect to concentration of test substance and $\alpha$ allows for any variance of the normalised response between plates which occurs independently of the test chemical. The length-scale parameter $\rho$ controls the between-concentration correlation in the response. The correlation function above biases the normalised response to be more highly correlated within a plate the further the test concentration is away from $\theta$. This reduces the tendency to overfit to the upper part of sigmoidal-like concentration-response shapes. The parameters $\alpha$, $\eta$ and $\rho$ are assigned the prior distributions

The prior distribution for $\theta$ depends on whether the test substance is one of the positive controls or not. For test substances which are not the positive control, the maximum concentration of the test substance was set close to a cytotoxic concentration determined from a preliminary cytotoxicity study and the lowest concentration 4^7^=16,384-fold lower than this (using a 4-fold dilution series, with eight test concentrations). The concentration range is designed to ensure there is a high probability that the true value of $\theta$ falls within the tested concentration range, in the event that the test substance induces a change in the measured response. The prior on $\theta$ in this case is

.

This prior has the property that the a priori probability of a chemical inducing a response less than the maximum test concentration ($\theta<\max(\mathbf{x})$) is 0.5 (maximally uncertain). Furthermore, this prior penalises values of $\theta$ as the test concentration decreases below the maximum concentration tested, with stronger penalties for values of $\theta$ below the minimum tested concentration.

If instead the test substance is a positive control, test concentrations are those chosen historically by Cyprotex. For these concentration ranges, it is often the case that there is a strong response at the lowest concentration tested and consequently the prior on $\theta$ is changed to

.

This prior distribution assigns a small probability to the event that the test substance does not induce a response ($\theta>\max(\mathbf{x})$). The reduction in the scale of the prior is there to heavily penalise thresholds far below the minimum test concentration. The penalty is applied on the basis that if a response is induced at the minimum test concentration, the threshold no effect concentration $\theta$ is poorly identified from the available data.

The sampling distribution for DMSO control responses is specific to the plate design used. Let $z_{j,k,l}$ be the $k$th repeat DMSO control response on plate $j$ in column $l$. The sampling distribution is

$$z_{j,k,l}\sim t\left(\nu,z_{j,l},\sigma\right)$$

where $\nu$ and $\sigma$ are as defined above. The median response in each column, on each plate, is assigned the sampling distribution

with $\alpha$ the same as above. In this specification, the sampling distribution for DMSO control responses mirror the sampling distribution for treatment responses when the test concentration is less than the minimum effect concentration $\theta$. However, all DMSO control responses within a column are assumed to be uncorrelated with each other.

***Concentration-response curves***

The sampling statements in the previous section collectively define the right hand side of equation for each concentration-response dataset. The posterior distribution (left hand side of equation ) is used to define concentration-response curves compatible with the data. Concentration-response curves are evaluated over a vector of 100 hypothetical treatment concentrations ranging from $\theta$ to the maximum tested experimental treatment concentration. Denote by $\mathbf{x}'$ the vector of treatment concentrations. The expected response for biological replicate $j$ is given by $\mathbf{y}'$ which is calculated as the marginal distribution of a multivariate normal such that

$\mathbf{y}'_{j}=K\left(\mathbf{x}',\mathbf{x}\right)K(\mathbf{x},\mathbf{x})^{-1}\mathbf{y}_{j}$,

where the matrix $K$ is defined in equation .

***Distribution of PoD and concentration-dependency score***

PoDs are calculated as the first concentration at which the expected response deviates by one normalised unit away from the expected response at $\theta$. The joint distribution of the model parameters induces a distribution over possible concentration-response curves compatible with the data for each biological replicate which in turn induces a distribution of the PoD for each biological replicate. The proportion of the PoD distribution less than the maximum concentration-tested is termed the *concentration dependency score* (CDS) and is taken as a statistical measure of the confidence that each biological replicate induces a response in the measured biomarker at a test concentration less than the maximum tested. Mathematically, the CDS is the cumulative density function of the distribution of the PoD evaluated at the maximum tested test concentration.

**Computation**

Data processing, normalisation and visualisation was performed using Python 3.8 and packages NumPy 1.19, pandas 1.2, SciPy 1.5, matplotlib 3.3. Evaluation of posterior distribution is achieved using MCMC sampling via use of the methods provided in the probabilistic programming language Stan (Carpenter et al., 2017a). For each dataset, 4 chains of length 5,000 samples are generated, discarding the first half of each chain, leaving 10,000 samples from the posterior distribution. Additional arguments to Stan include setting the *adapt_delta* parameter to 0.95.

***Multimodality***

Occasionally, MCMC chains do not mix well within the posterior (as determined by the convergence check$\hat{R}\neq1)$ indicating multimodality in the posterior distribution. Multimodality may occur when multiple, distinct values of the PoD would be consistent with the model and data. When this occurs, concentration-response plots are inspected visually and the prior distribution on $\theta$ is adjusted to bias the PoD estimate to a chosen part of the concentration range. Observed responses in other datasets may be used to make this judgement. Refitting the model using the more informative prior usually resolves convergence problems. In the very rare occasion that multimodality occurs even under the restricted prior, we run 4 chains of length 20,000 samples and retain the chain with the lowest expected value of $\theta$. Any bespoke choices made at this stage are recorded in the summary reports containing concentration-response fits.

**Dataset summary**

In total, 1215 concentration-response datasets were analysed using this approach. Datasets are obtained from 18 assays covering 42 distinct biomarkers. 12 chemicals have data for every assay and biomarker and a further 22 chemicals have data for specific assays and biomarkers because they are used as positive controls.

## M3. Concentration-response analysis of HTTr data using the BIFROST method

**Overview**

The BIFROST (Bayesian inference for region of signal threshold) approach proceeds firstly, by inferring PoDs, independently, at the probe level. Secondly, a global PoD is inferred in terms of the lowest (with respect to dose) responding probes. Probe-level PoDs are inferred as probability distributions encompassing the uncertainty in both location of the PoD and differential expression following exposure less than the maximum tested concentration. The probability distribution for a PoD is obtained from the posterior probability distribution of a set of model parameters of a concentration-response model conditioned on the raw counts for a probe. This approach was first published in (Reynolds et al., 2020).

The BIFROST approach has some notable differences to tools such as BMDexpress2 (Phillips et al., 2019). A major difference is the use of Bayesian statistics to infer model parameters, in contrast to maximum likelihood estimation. The use of Bayesian statistics defines a natural framework for both regularisation of parameter estimates and uncertainty quantification. In contrast, BMDexpress2 uses non-regularised maximum likelihood estimation and bootstrapping techniques for estimation of PoD uncertainty. A second major difference is the use of a semi-parametric model for concentration-response modelling in the BIFROST approach – the flexibility of which allows the same model to be fit to the data for every probe. In contrast, in BMDexpress2, a small set of parametric models is fit to the data and the ‘best’ model is selected using the Akaike information criterion. Lastly, the BIFROST method uses raw counts for each probe directly. Factors which might be used to normalise data prior to concentration-response modelling in BMDexpress2 are applied to model parameters, rather than the data itself. This approach allows for greater complexity is the description of the variance structure of the data - which is required for robust PoD inferences.

**Definitions**

**Treatment** – exposing cells to some chemical of interest for the purposes of assessing whether the exposure results in differential genetic expression.

**Control group** – collection of samples of the solvent control which are grouped for the purpose of concentration-response analysis.

**Plate Design**

Cells are treated with the test substance(s) within a 384-well plate. The arrangement of treatments is illustrated in Figure 3. Briefly, all treatments for a single test substance are located within a single column of the plate (notation CX TY corresponds to chemical X, treatment concentration Y). There are seven distinct treatment concentrations, with a single technical replicate of each, located in every second row. The highest treatment concentrations are located in rows B and C. Columns 2, 6 and 10 contains DMSO controls (notation DMSO). Positive controls (trichostatin A and tunicamycin) are located in column 9. A separate plate is used for the five biological replicates.

Figure 3 Plate design used for the HTTr platform. Each column holds either a) all treatments for a single compound or b) all treatments for a solvent control or c) DMSO solvent controls.

**Data processing**

***Removal of probes with low counts***

Data processing begins by first establishing a matrix of raw counts with samples for the treatment and solvent controls as columns and probes as rows.

Probes with a mean or median raw count less than five across all treatment samples are discarded. Counts for solvent controls are ignored at this step.

***Sample filtering***

Samples are discarded if any of the following are true:

1. The mean raw count across all probes is less than 100.
2. The raw count is equal to zero for more than 25% of probes.
3. The percentage of mapped reads is less than 50% for the sample.

***Assignment to treatment and control groups***

Samples located at the same position on each plate are grouped for the purposes of concentration-response analysis. This results in distinct groups for each treatment concentration and several groups of solvent control samples.

Solvent controls are located in three columns on the plate. In the first two columns, solvent controls are location within every second row. In the final column, solvent controls are located in every row. Solvent controls are further divided up into four ‘control columns’ which contain solvent controls from every adjacent row. Thus, the last, full column of solvent controls is split into two groups at this step. The defined groups correspond to distinct dosing plates.

**Concentration-response analysis**

***Indexing***

Every count is assigned a sequence of indices used to define the data structure. These include:

- An index $i=1,\ldots,N_{\text{probe}}$ denoting the probe to which the count corresponds. $N_{\text{probe}} \sim13,000$ for each dataset.
- An index $j=1,\ldots,N_{\text{well}}$ denoting the position (well) within the exposure plate. $N_{\text{well}}\sim35$ for each dataset.
- An index $k=1,\ldots,N_{\text{plate}}$ denoting the plate from which the count was measured. $N_{\text{plate}}=5$ for each dataset.
- An index $l=1,\ldots,N_{\text{column}}$ denoting the column group of the count. $N_{\text{column}}=5$ for each dataset, 1 corresponding to the treatment, 4 for solvent controls.

Note, the column group $l$ is fixed for each index $j$ and so to simplify notation below is dropped with the exception of specifying parameters specific to a column group.

***Treatment concentration***

The nominal concentration of test substance in well $j$ is denote as $x_{j}$. For solvent control wells, $x_{j}=0$.

***Estimation of sample bias***

The bias in counts in each sample is estimated as in (Reynolds et al., 2020). Denote by $\beta_{j,k}$ the estimate of bias in the sample in well $j$ on plate $k$, then

$$\beta_{j,k}=\text{median}_{i}\left(\frac{c_{i,j,k}}{\text{median}_{\text{j},\text{k}}\left(c_{\text{i},\text{j},\text{k}}\right)}\right)$$

where $c_{i,j,k}$ is the raw count for probe $i$ in group $j$ on plate $k$.

**Model structure**

***Likelihood of observing raw count***

The sampling distribution of the raw count of probe $i$ in well $j$ on plate $k$ is assumed to be

$$c_{i,j,k}\sim\text{Poisson}\left(\beta_{j,k}\exp\left(y_{i,j,k}\right)\right)$$

where $y_{i,j,k}$ is the natural logarithm of the expected count for probe $i$ in well $j$ on plate $k$.

*Variability between plates*

The natural logarithm of the expected count between plates is assumed to vary according to a Student’s t-distribution with degrees of freedom $\nu$. The sampling distribution is expressed as

$$y_{i,j,k}\sim t\left(\nu,\bar{y}_{i,j},\sigma\right)$$

where $\bar{y}_{i,j}$ is the mean of the natural logarithm of the expected count across plates for probe $i$ in well $j$. The scale parameter $\sigma$ governs the between-plate variance. Note that the above two equations improve upon the published approach in which a negative binomial distribution was used since the higher kurtosis this distribution allows offers increased robustness to outliers (Reynolds et al., 2020).

***Variability between groups***

Variability between treatment groups is modelled as

where $\mathbf{\mu}_{i,l}$ is the vector $\left[\mu_{i,l},\mu_{i,l},\ldots,\mu_{i,l}\right]^{T}$ of length equal to the number of treatment groups. The repeated component $\mu_{i,l}$ is defined as the expected natural logarithm of the count for a treatment concentration equal to zero for probe $i$ in column group $l$. This structure implies that we explicitly allow the baseline count to be distinct for column groups. The scale parameter $s_{i}$ allows for variability between the average response in each well. This offers protection against any systematic drift in the response due to position on the plate. is the identity matrix and $K_{i}$ is the kernel of the Gaussian process used to describe correlation in the response due to the treatment concentration, defined as

$$K_{i}\left(x_{u},x_{v}\right)=\left\{\begin{matrix} 0 & \text{if} x_{u}\leq0 \text{or} x_{v}\leq0 , \\ \eta_{i}^{2}\left(x_{u}-\theta_{i}\right)^{2}\left(x_{v}-\theta_{i}\right)^{2}\exp\left(-\frac{\left(x_{u}-x_{v}\right)^{2}}{\rho_{i}^{2}\left(1+\left(x_{u}-\theta_{i}\right)^{2}\right)\left(1+\left(x_{v}-\theta_{i}\right)^{2}\right)}\right) & \text{if} x_{u}>0 \text{and} x_{v}>0 , \end{matrix}\right.$$

where $\theta_{i}$ is the threshold concentration below which the measured response in each well can be considered independent of other wells for probe $i$. $\eta_{i}$ governs how quickly the response may grow above the threshold $\theta_{i}$ and $\rho_{i}$ controls the length scale (smoothness) of the change in the expected response.

***Shrinkage for baseline response***

Whilst the baseline response is allowed to be distinct for each column group, estimates are shrunk towards each other using the hierarchical structure

.

**Prior distributions**

The following prior distributions are as specified in (Reynolds et al., 2020), with the exception of $s_{i}$ which is converted to a boundary-avoiding prior and $\sigma_{i}$ and $\mu_{i}^{c}$ which are not defined in this reference. Table 1 (below) provides a summary of the model parameters and associated sampling distributions.

,

,

$\nu\sim\text{Gamma}\left(2,0.1\right)$,

$s_{i}\sim\text{InverseGamma}\left(1,0.1\right)$,

,

,

$\eta_{i}\sim\text{InverseGamma}\left(3,10\right)$,

$\rho_{i}\sim\text{InverseGamma}\left(3,10\right)$.

**Concentration-response curves for estimation for points-of-departure**

Concentration-response curves are evaluated over 100 uniformly spaced values from $\theta_{i}$ to $\max_{j}x_{j}$. Let $\mathbf{x}'$ be the vector of concentration values over this range and $\mathbf{y}'$ the corresponding expected response. Then

where $\mathbf{\mu}_{i,l}$ and $\mathbf{\mu}'_{i,l}$ are vectors containing the parameter $\mu_{i,l}$ ($l$ denoting the treatment column group) of length equal to the number of distinct treatment doses and 100, respectively. The vector $\mathbf{x}$ is composed of the elements $x_{j}$ where $j$ indexes a treatment group and the vector $\bar{\mathbf{y}}_{i}$ is the corresponding vector of the responses $\bar{y}_{i,j}$ in these groups.

**Probe-level points-of-departure**

Let $z_{i}$ be the distribution of the natural logarithm of the expected count for probe $i$ assuming a treatment concentration of zero. The sampling distribution is induced as

A point of departure for probe $i$ is denoted as $\hat{x}_{i}$ and defined as the lowest treatment concentration such that the expected response intersects the boundaries of a centred 95% credible interval for $z_{i}$.

**Global point-of-departure**

A global point-of-departure is defined as in (Reynolds et al., 2020), subject to a decrease in the range of quantiles considered for contribution to the PoD. The minimum PoD (across probes) at quantile $q$ is defined as

$$\text{PoD}_{\min}(q)=\inf_{i} F_{i}^{-1}(q)$$

where $F_{i}^{-1}$ is the quantile function (inverse CDF) of the PoD for probe $i$. The global PoD is defined as

$$\text{PoD}_{\text{global}}=\int_{0.25}^{0.75} \text{PoD}_{\min}(q)p(q) \text{d}q$$

where $p(q)$ is a uniform distribution on the quantile $q$.

**Concentration-response curves for visualisation**

When visualising concentration-response curves, the vector $\mathbf{x}'$ is defined as 100 uniformly spaced points between the minimum and maximum treatment concentrations, excluding zero.

| Parameter | Definition | Prior/sampling distribution |
| --- | --- | --- |
| $c_{i,j,k}$ | Raw count for probe $i$ in group $j$ on plate $k$ | $\text{Poisson}\left(\beta_{j,k}\exp\left(y_{i,j,k}\right)\right)$ |
| $\beta_{j,k}$ | Estimate of the bias in raw counts for the sample in group $j$ on plate $k$ | Point estimate: $\beta_{j,k}=\text{median}_{i}\left(\frac{c_{i,j,k}}{\text{median}_{\text{j},\text{k}}\left(c_{\text{i},\text{j},\text{k}}\right)}\right)$ |
| $x_{j}$ | Base-10 logarithm of the treatment concentration for group $j$ | NA – value defined by experimental design |
| $y_{i,j,k}$ | Natural logarithm of the expected count for probe $i$ in group $j$ on plate $k$ | $t\left(\nu,\bar{y}_{i,j},\sigma\right)$ |
| $\bar{y}_{i,j}$ | Expected value of $y_{i,j,k}$ after averaging across plates |  |
| $\nu$ | Degrees of freedom parameter governing shape of sampling distribution for biological replicates within the same position | $\nu\sim\text{Gamma}\left(2,0.1\right)$ |
| $\sigma_{i}$ | Scale parameter governing variability of $y_{i,j,k}$ between plates | $\text{HalfNormal}\left(0,1\right)$ |
| $\mu_{i}$ | Expected value of $\bar{y}_{i,j}$ for treatment groups when the concentration is zero |  |
| $\mu_{i,l}$ | Expected value of $\bar{y}_{i,j}$ across all control groups |  |
| $\sigma_{i}^{\text{column}}$ | Standard deviation of baseline log count across column groups. |  |
| $s_{i}$ | Standard deviation of $\bar{y}_{i,j}$ owing to non-treatment variability such as position on plate | $\text{InverseGamma}\left(1,0.1\right)$ |
| $\theta_{i}$ | Threshold treatment concentration below which treatment counts are assumed to be uncorrelated |  |
| $\eta_{i}$ | Hyperparameter of GP kernel governing how quickly the response can grow with respect to concentration | $\eta_{i}\sim\text{InverseGamma}\left(3,10\right)$ |
| $\rho_{i}$ | Hyperparameter of GP kernel governing the length scale (smoothness) of the response | $\rho_{i}\sim\text{InverseGamma}\left(3,10\right)$ |

Table 1 Summary of model parameters and associated sampling/prior distributions.

**Computation**

Data processing and visualisation is realised using Python 3.8 with the packages NumPy 1.19, pandas 1.2, pystan 2.19 and SciPy 1.6. Scripts for visualisation use matplotlib 3.3. The concentration-response model defined above is realised in the programming language Stan (interfaced to using PyStan) (Carpenter et al., 2017b).

For each probe, 20 chains of length 1000 iterations are run. The final set of samples is thinned by a factor of 10 to keep file sizes manageable. Final output contains 1000 samples for the PoD for each probe.

## M4. Probability of BER >1

A distribution for the bioactivity exposure ratio is calculated owing to uncertainty in the PBK C_max_ estimate. This distribution is calculated using PBK prediction uncertainty model for each benchmark exposure and each C_max_ for the three PBK parameterisation levels. The probability that the BER exceeds one is listed in Table 2. Visual representations of distribution are presented in Figure 4.

Table 2 Probability that BER>1 for different Cmax estimates for each each benchmark exposure.

| **Chemical** | **Route** | **Exposure** | **Level** | **Risk** | **Prob. BER>1** | **BER 2.5th quantile** | **BER 50th quantile** | **BER 97.5th quantile** |
| --- | --- | --- | --- | --- | --- | --- | --- | --- |
| Butylated hydroxytoluene | Dermal | Body Lotion, 0.5% | L1 | Low | 0.85 | 0.10 | 11 | 1100 |
| Butylated hydroxytoluene | Dermal | Body Lotion, 0.5% | L2 | Low | 0.97 | 0.85 | 17 | 370 |
| Butylated hydroxytoluene | Dermal | Body Lotion, 0.5% | L3 | Low | 1.00 | 13 | 47 | 180 |
| Caffeine | Dermal | 2 mg/cm2, 25 cm2 | L1 | Low | 1.00 | 54 | 3400 | 230000 |
| Caffeine | Dermal | 2 mg/cm2, 25 cm2 | L2 | Low | 1.00 | 2.9 | 62 | 1300 |
| Caffeine | Dermal | 2 mg/cm2, 25 cm2 | L3 | Low | 1.00 | 13 | 46 | 170 |
| Caffeine | Oral | Food & Drink, 400 mg/day | L1 | Low | 0.42 | 0.0057 | 0.63 | 63 |
| Caffeine | Oral | Food & Drink, 400 mg/day | L2 | Low | 0.08 | 0.0050 | 0.11 | 2.6 |
| Caffeine | Oral | Food & Drink, 400 mg/day | L3 | Low | 0.01 | 0.054 | 0.20 | 0.77 |
| Caffeine | Oral | Overdose, 10g | L1 | High | 0.06 | 0.00022 | 0.024 | 2.5 |
| Caffeine | Oral | Overdose, 10g | L2 | High | 0.00 | 0.00038 | 0.0083 | 0.19 |
| Caffeine | Oral | Overdose, 10g | L3 | High | 0.00 | 0.0020 | 0.0080 | 0.032 |
| Caffeine | Dermal | Shampoo, 0.2% | L1 | Low | 1.00 | 17 | 1700 | 180000 |
| Caffeine | Dermal | Shampoo, 0.2% | L2 | Low | 1.00 | 8.6 | 200 | 4400 |
| Caffeine | Dermal | Shampoo, 0.2% | L3 | Low | 1.00 | 80 | 290 | 1100 |
| Coumarin | Oral | 0.1 mg/kg bw/day | L1 | Low | 0.81 | 0.089 | 7.1 | 630 |
| Coumarin | Oral | 0.1 mg/kg bw/day | L2 | Low | 1.00 | 4.2 | 89 | 1700 |
| Coumarin | Oral | 0.1 mg/kg bw/day | L3 | Low | 1.00 | 57 | 200 | 730 |
| Coumarin | Dermal | Body Lotion, 0.38% | L1 | Low | 0.69 | 0.034 | 3.3 | 390 |
| Coumarin | Dermal | Body Lotion, 0.38% | L2 | Low | 0.87 | 0.28 | 6.0 | 130 |
| Coumarin | Dermal | Body Lotion, 0.38% | L3 | Low | 0.99 | 1.6 | 5.8 | 22 |
| Coumarin | Oral | Food, 4.1 mg/day | L1 | Low | 0.88 | 0.18 | 15 | 1500 |
| Coumarin | Oral | Food, 4.1 mg/day | L2 | Low | 1.00 | 8.5 | 180 | 3800 |
| Coumarin | Oral | Food, 4.1 mg/day | L3 | Low | 1.00 | 110 | 410 | 1500 |
| Doxorubicin | Intravenous | 4.5 mg/m2/day continuous infusion for four days | L1 | High | 0.03 | 0.00013 | 0.012 | 1.2 |
| Doxorubicin | Intravenous | 4.5 mg/m2/day continuous infusion for four days | L2 | High | 0.00 | 0.00089 | 0.017 | 0.33 |
| Doxorubicin | Intravenous | 4.5 mg/m2/day continuous infusion for four days | L3 | High | 0.00 | 0.026 | 0.098 | 0.38 |
| Doxorubicin | Intravenous | 75 mg/m2/day for 10 minutes | L1 | High | 0.00 | 1.9E-06 | 0.00023 | 0.026 |
| Doxorubicin | Intravenous | 75 mg/m2/day for 10 minutes | L2 | High | 0.00 | 1.4E-05 | 0.00031 | 0.0063 |
| Doxorubicin | Intravenous | 75 mg/m2/day for 10 minutes | L3 | High | 0.00 | 3.2E-05 | 0.00012 | 0.00043 |
| Hexylresorcinol | Dermal | Face Serum, 0.5% | L1 | Low | 0.67 | 0.027 | 2.7 | 280 |
| Hexylresorcinol | Dermal | Face Serum, 0.5% | L2 | Low | 0.81 | 0.19 | 4.0 | 77 |
| Hexylresorcinol | Oral | Food residues, 0.0033 mg/kg bw/day | L1 | Low | 0.93 | 0.32 | 35 | 3700 |
| Hexylresorcinol | Oral | Food residues, 0.0033 mg/kg bw/day | L2 | Low | 0.99 | 2.5 | 51 | 1100 |
| Hexylresorcinol | Oral | Throat Lozenge, 2.4 mg | L1 | Low | 0.56 | 0.014 | 1.4 | 150 |
| Hexylresorcinol | Oral | Throat Lozenge, 2.4 mg | L2 | Low | 0.63 | 0.076 | 1.7 | 32 |
| Niacinamide | Dermal | Body Lotion, 3% | L1 | Low | 0.69 | 0.026 | 3.1 | 380 |
| Niacinamide | Dermal | Body Lotion, 3% | L2 | Low | 0.56 | 0.062 | 1.3 | 26 |
| Niacinamide | Dermal | Body Lotion, 3% | L3 | Low | 0.95 | 0.75 | 2.9 | 11 |
| Niacinamide | Oral | Food & Drink, 12.5 mg/kg bw/day | L1 | Low | 0.51 | 0.0090 | 1.1 | 100 |
| Niacinamide | Oral | Food & Drink, 12.5 mg/kg bw/day | L2 | Low | 0.22 | 0.013 | 0.30 | 6.2 |
| Niacinamide | Oral | Food & Drink, 12.5 mg/kg bw/day | L3 | Low | 0.02 | 0.044 | 0.19 | 0.83 |
| Niacinamide | Oral | Food & Drink, 22.2 mg/day | L1 | Low | 0.95 | 0.43 | 39 | 3800 |
| Niacinamide | Oral | Food & Drink, 22.2 mg/day | L2 | Low | 0.95 | 0.57 | 12 | 250 |
| Niacinamide | Oral | Food & Drink, 22.2 mg/day | L3 | Low | 1.00 | 2.0 | 7.5 | 28 |
| Niacinamide | Dermal | Hair Conditioner, 0.1% | L1 | Low | 1.00 | 170 | 16000 | 1600000 |
| Niacinamide | Dermal | Hair Conditioner, 0.1% | L2 | Low | 1.00 | 340 | 7500 | 170000 |
| Niacinamide | Dermal | Hair Conditioner, 0.1% | L3 | Low | 1.00 | 1900 | 7100 | 26000 |
| Oxybenzone | Dermal | Body Lotion, 0.5% | L1 | Low | 0.44 | 0.0061 | 0.72 | 83 |
| Oxybenzone | Dermal | Body Lotion, 0.5% | L2 | Low | 0.50 | 0.047 | 0.98 | 22 |
| Oxybenzone | Dermal | Body Lotion, 0.5% | L3 | Low | 0.69 | 0.37 | 1.3 | 4.9 |
| Oxybenzone | Dermal | Sunscreen, 2% | L1 | Low | 0.14 | 0.00081 | 0.085 | 9.4 |
| Oxybenzone | Dermal | Sunscreen, 2% | L2 | Low | 0.12 | 0.0074 | 0.17 | 3.6 |
| Oxybenzone | Dermal | Sunscreen, 2% | L3 | Low | 0.02 | 0.071 | 0.26 | 0.93 |
| Paraquat dichloride | Oral | Pesticide poisoning, 35 mg/kg/day | L1 | High | 0.00 | 1.3E-05 | 0.0014 | 0.16 |
| Paraquat dichloride | Oral | Pesticide poisoning, 35 mg/kg/day | L2 | High | 0.00 | 9.9E-05 | 0.0022 | 0.045 |
| Rosiglitazone | Oral | Medical, 1 mg/12 hours | L1 | High | 0.12 | 0.00052 | 0.053 | 6.5 |
| Rosiglitazone | Oral | Medical, 1 mg/12 hours | L2 | High | 0.02 | 0.0017 | 0.036 | 0.79 |
| Rosiglitazone | Oral | Medical, 1 mg/12 hours | L3 | High | 0.00 | 0.010 | 0.036 | 0.14 |
| Rosiglitazone | Oral | Medical, 8 mg/day | L1 | High | 0.03 | 8.5E-05 | 0.0094 | 1.2 |
| Rosiglitazone | Oral | Medical, 8 mg/day | L2 | High | 0.00 | 0.00030 | 0.0075 | 0.17 |
| Rosiglitazone | Oral | Medical, 8 mg/day | L3 | High | 0.00 | 0.0013 | 0.0055 | 0.022 |
| Sulforaphane | Oral | Food & Drink, 3.9 mg/day | L1 | Low | 0.34 | 0.0036 | 0.37 | 37 |
| Sulforaphane | Oral | Food & Drink, 3.9 mg/day | L2 | Low | 0.43 | 0.032 | 0.76 | 18 |
| Sulforaphane | Oral | Tablet, 60 mg/day | L1 | Low | 0.12 | 0.00073 | 0.069 | 6.8 |
| Sulforaphane | Oral | Tablet, 60 mg/day | L2 | Low | 0.11 | 0.0075 | 0.16 | 3.6 |


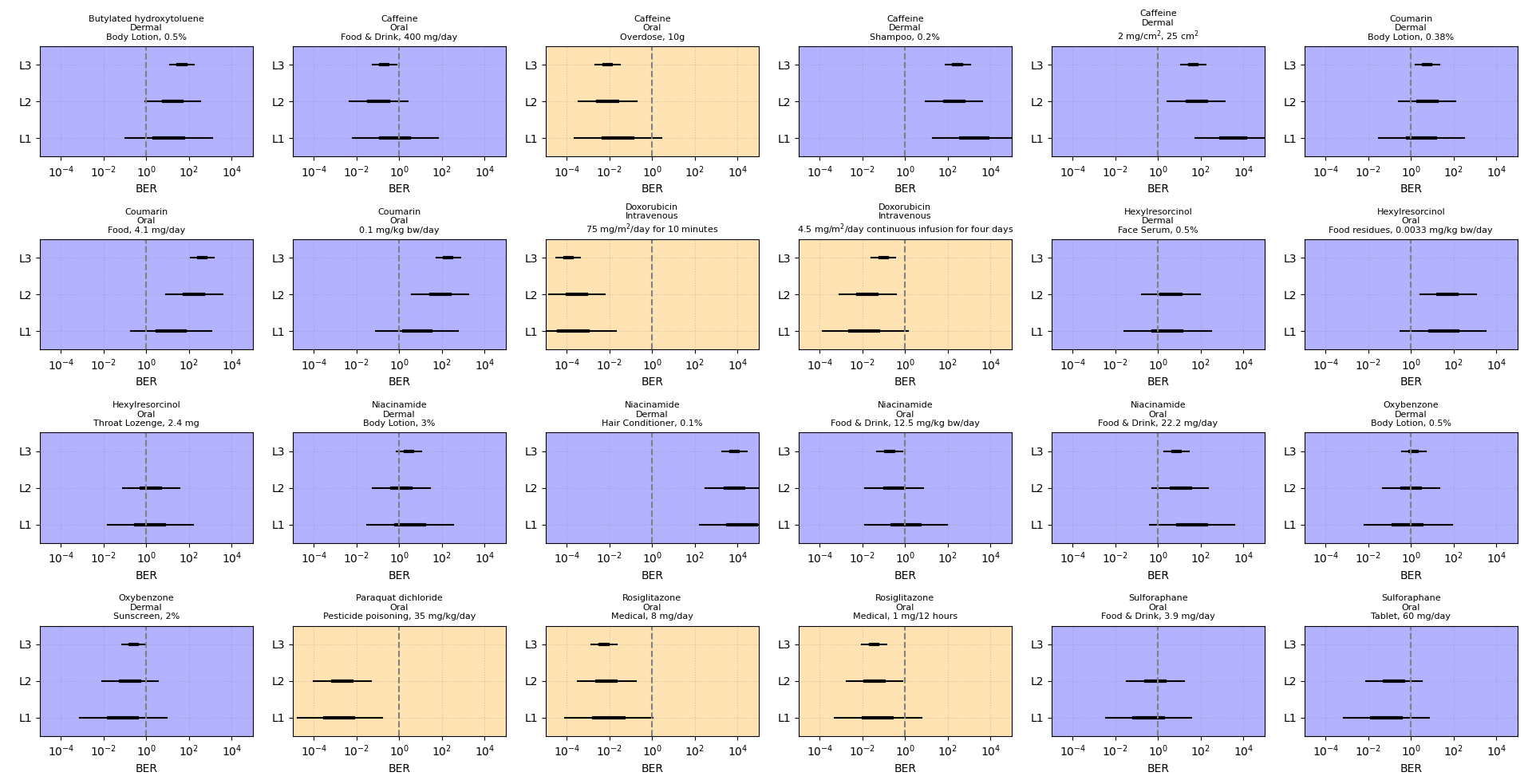


Figure 4 Interval summary of the BER distribution for each PBK prediction. Thin lines span a 95% centred interval of the BER distribution and thick lines span a 50% interval. Background colours indicate the assigned risk classification at Stage 1 for each exposure.

## M5. Consistent Decision Making

A sound decision model will allow an exposure to be classified as low risk at a low PBK parameterisation level if the BER is sufficiently large. However, PBK predictions at low levels carry significant error and it is very possible that the predicted C_max_ will increase considerably at the next PBK parameterisation level. It is desirable that ‘low risk’ classifications at low PBK parameterisation levels remain low risk at higher levels, even if the C_max_ is found to increase. This is achieved by adopting stricter thresholds on the BER to conclude low risk at lower levels. The calculations below use the PBK prediction error model to derive these thresholds.

### Distributions of BERs predicted by the CMED model

The CMED model posits sampling distributions for level $x$ log10 PBK estimates conditional on level $x+1$. These sampling distributions are

,

,

,

as specified in the main manuscript. In the absence of a measured C_max_ estimate and noting that the prior distribution on $\zeta_{i,j}$ is uniform and Gaussians are assumed, these sampling distributions are reversible such that

,

,

.

Furthermore, letting

$$\begin{matrix} \sigma_{\text{L3}}=\sigma_{\text{L3},\text{pop}} , \\ \sigma_{\text{L2}}=\frac{\sigma f_{i,j}^{\text{L2},\text{L3}}}{\beta+f_{i,j}^{\text{L2},\text{L3}}} , \\ \sigma_{\text{L1}}=\frac{\sigma f_{i,j}^{\text{L1},\text{L2}}}{\beta+f_{i,j}^{\text{L1},\text{L2}}} , \end{matrix}$$

and noting that the result of compounding a Gaussian distribution whose mean is itself a Gaussian is, in fact, Gaussian, we can express the sampling distribution for the log10 population mean C_max_ as

,

where $u=1,2,3$ indexes PBK levels 1, 2 and 3, respectively.

Let $b_{i,j}=\theta_{i}-\zeta_{i,j}$ be the distribution of the BER for chemical $i$ and exposure $j$ given chemical-specific POD $\theta_{i}$. Furthermore, denote by $\hat{b}_{i,j}^{u}=\theta_{i}-\hat{\zeta}_{i,j}^{u}$ the point-estimate BER at PBK level $u$. PODs are summarised as point values. It follows that the sampling distribution of BER, conditional on the point-estimate BER is

.

For notational convenience, in the next section subscripts $i$ and $j$ indexing chemical and exposure, respectively are dropped and superscript $u$ is changed to a subscript.

### Derivation of confidence thresholds to ensure low risk decisions are robust

Suppose a decision model is defined such that for a given BER threshold $b^{*}$(within the manuscript we set $b^{*}=\text{log}_{10}(1)=0$) we accept the exposure as low risk if the proportion of BER distribution greater than the threshold exceeds a confidence threshold $q_{u}^{*}$, where $u$, as above, indexes the PBK parameterisation level. If an exposure is deemed low risk, it is undesirable if the same exposure would be considered uncertain risk at the next level of the PBK framework as a consequence of obtaining a higher C_max_ estimate. Therefore, we must choose the decision parameters appropriately to minimise the chance that this event occurs.

Assume an exposure is acceptable at level $u$ of the PBK framework with $u$ equal to either L1 or L2. Then by construction of the decision model we must have

$$\text{Pr}\left(b>b^{*}\mid\hat{b}_{u}\right)=q\geq q_{u}^{*}$$

where $b$ is a random variable representing the true BER, $\hat{b}_{u}$ is the point estimate of the BER obtained from the PBK prediction at level $u$ and $q_{u}^{*}$ is the proportion of the BER distribution which must exceed $b^{*}$ for the exposure to be classified as “low risk”. As discussed in the previous section, the CMED model gives us an estimate of the true BER distribution conditional on point-estimate at level $u$ as

,

and therefore equation is equivalent to the condition

$\hat{b}_{u}=b^{*}-\sum_{k=u}^{3} \alpha_{k}-\sqrt{\sum_{k=u}^{3} \sigma_{k}^{2}}\Phi^{-1}\left(1-q\right)$,

where $\Phi$ is the cumulative density function of a standard normal distribution.

The same exposure supportable at the next level $u+1$ of the PBK framework if

$\text{Pr}\left(b>b^{*}\mid\hat{b}_{u+1}\right)\geq q_{u+1}^{*} \Rightarrow\hat{b}_{u+1}>b^{*}-\mathop{\sum^{3} }\limits_{k=u+1} \alpha_{k}-\sqrt{\sum_{k=u+1}^{3} \sigma_{k}^{2}}\Phi^{-1}\left(1-q_{u+1}^{*}\right)$.

From the CMED model it also follows that

.

and so, under this model, the probability that an exposure is supportable at the next level of the PBK framework is

$$\begin{matrix} \text{Pr}\left(\hat{b}_{u+1}>b^{*}-\sum_{k=u+1}^{3} \alpha_{k}-\sqrt{\sum_{k=u+1}^{3} \sigma_{k}^{2}}\Phi^{-1}\left(1-q_{u+1}^{*}\right)\right) & =1-\text{Pr}\left(\hat{b}_{u+1}\leq b^{*}-\sum_{k=u+1}^{3} \alpha_{k}-\sqrt{\sum_{k=u+1}^{3} \sigma_{k}^{2}}\Phi^{-1}\left(1-q_{u+1}^{*}\right)\right) \\ & =1-\Phi\left(\frac{b^{*}-\sum_{k=u+1}^{3} \alpha_{k}-\sqrt{\sum_{k=u+1}^{3} \sigma_{k}^{2}}\Phi^{-1}\left(1-q_{u+1}^{*}\right)-\hat{b}_{u}-\alpha_{u}}{\sigma_{u}}\right) \\ & =1-\Phi\left(\frac{b^{*}-\sum_{k=u+1}^{3} \alpha_{k}-\sqrt{\sum_{k=u+1}^{3} \sigma_{k}^{2}}\Phi^{-1}\left(1-q_{u+1}^{*}\right)-b^{*}+\sum_{k=u}^{3} \alpha_{k}+ \sqrt{\sum_{k=u}^{3} \sigma_{k}^{2}}\Phi^{-1}\left(1-q\right)-\alpha_{u}}{\sigma_{u}}\right) \\ & =1-\Phi\left(\frac{ \sqrt{\sum_{k=u}^{3} \sigma_{k}^{2}}\Phi^{-1}\left(1-q\right)-\sqrt{\sum_{k=u+1}^{3} \sigma_{k}^{2}}\Phi^{-1}\left(1-q_{u+1}^{*}\right)}{\sigma_{u}}\right) . \end{matrix}$$

Denote by $r$ the chance that an acceptable exposure remains acceptable, under this decision model, at the next level of PBK framework. To minimise the chance that an acceptable exposure decision would be overturned at a higher level of the PBK framework we must choose $q_{u}^{*}$, $q_{u+1}^{*}$ and $r$ such that

$\text{Pr}\left(\hat{b}_{u+1}>b^{*}-\sqrt{\sum_{k=u+1}^{3} \sigma_{k}^{2}}\Phi^{-1}\left(1-q_{u+1}^{*}\right)\right)\geq r$,

which following some algebra can be expressed as

$\sigma_{u}\Phi^{-1}\left(1-r\right)+\sqrt{\sum_{k=u+1}^{3} \sigma_{k}^{2}}\Phi^{-1}\left(1-q_{u+1}^{*}\right)\geq\sqrt{\sum_{k=u}^{3} \sigma_{k}^{2}}\Phi^{-1}\left(1-q\right)$ .

For the exposure to have been acceptable at level $i$ of the PBK framework we have $q\geq q_{u}^{*}$ and therefore a sufficient condition for equation to hold is choosing $q_{u}^{*}$, $q_{u+1}^{*}$ and $r$ such that

$\sigma_{u}\Phi^{-1}\left(1-r\right)+\sqrt{\sum_{k=u+1}^{3} \sigma_{k}^{2}}\Phi^{-1}\left(1-q_{u+1}^{*}\right)\geq\sqrt{\sum_{k=u}^{3} \sigma_{k}^{2}}\Phi^{-1}\left(1-q_{u}^{*}\right)$.

Given a choice for $r$ and $q_{u+1}^{*}$, and samples of $\alpha_{u}$and $\sigma_{u}, u=1,2,3,$ obtained from conditioning the CMED model on the available PBK data, we can sample $q_{u}^{*}$ as being the smallest value such that

$q_{u}^{*}\geq1-\Phi\left(\frac{\sigma_{u}\Phi^{-1}\left(1-r\right)+\sqrt{\sum_{k=u+1}^{3} \sigma_{k}^{2}}\Phi^{-1}\left(1-q_{u+1}^{*}\right)}{\sqrt{\sum_{k=u}^{3} \sigma_{k}^{2}}}\right)$ .

Clearly, the smallest value of $q_{u}^{*}$ is obtained when the inequality above is changed to an equality. This value can be translated into a threshold comparable to the point-estimate of the BER at the PBK level $u$ such that low risk is concluded with confidence if $\hat{b}_{u}\geq\hat{b}_{u}^{*}$ where

$\hat{b}_{u}^{*}=b^{*}-\sum_{k=u}^{3} \alpha_{k}-\sqrt{\sum_{k=u}^{3} \sigma_{k}^{2}}\Phi^{-1}\left(1-q_{u}^{*}\right)$ .

For streamlined decision making, the posterior expectation of $\hat{b}_{u}^{*}$ may be used as a threshold by which to compare the point-estimate BER forgoing the need to refit the CMED model and sample posterior distributions for the population average C_max_.

### Thresholds for consistent decision making

From the PBK uncertainty model we have

$\sigma_{1}=\frac{\sigma f_{i,j}^{\text{L1},\text{L2}}}{\beta+f_{i,j}^{\text{L1},\text{L2}}}\leq\frac{\sigma}{\beta+1}$,

$\sigma_{2}=\frac{\sigma f_{i,j}^{\text{L2},\text{L3}}}{\beta+f_{i,j}^{\text{L2},\text{L3}}}\leq\frac{0.5\sigma}{\beta+0.5}$,

$\sigma_{3}=\sigma_{\text{L3},\text{pop}}$.

Assuming the upper limits for $\sigma_{1}$ and $\sigma_{2}$, posterior expectations of these quantities are:

$$\begin{matrix} \text{E}\left[\sigma_{1}\right]\approx0.74, \\ \text{E}\left[\sigma_{2}\right]\approx0.59, \\ \text{E}\left[\sigma_{3}\right]\approx0.26, \\ \text{E}\left[\sqrt{\sum_{k=1}^{3} \sigma_{k}^{2}}\right]\approx0.99, \\ \text{E}\left[\sqrt{\sum_{k=2}^{3} \sigma_{k}^{2}}\right]\approx0.65. \end{matrix}$$

Posterior expectations of the bias parameters are

$$\begin{matrix} \text{E}\left[\alpha_{1}\right]=-0.14, \\ \text{E}\left[\alpha_{2}\right]=0.20, \\ \text{E}\left[\alpha_{3}\right]=0.028. \end{matrix}$$

We specify that 95% of the BER distribution must exceed one at PBK L3, $q_{3}^{*}=0.95$. Furthermore, we want at least a 90% chance of retaining a low-risk decision from PBK L2 to L3, $r=0.9$. Propagating the distributions of the parameters above through equations and , followed by calculating the means of the resulting distributions, we find $q_{2}^{*}=0.97$. For PBK L1 we calculate $q_{1}^{*}=0.98$. Translating these probabilities into thresholds on the point-estimate of the BER, the following decision model is defined:

1. Conclude low risk at PBK L1 if $\hat{b}_{1}>110$

2. Conclude low risk at PBK L2 if $\hat{b}_{2}>11$

3. Conclude low risk at PBK L3 if $\hat{b}_{3}>2.5$

# Supplementary Tables

## T1. Parameters for PBK Models

Physicochemical and ADME parameters (obtained from *in silico, in vitro* or calibrated against human clinical data) as input in the different PBK levels specified in the tables below.

#### Caffeine

|  | **Value** | **Source** | **Level** |
| --- | --- | --- | --- |
| Molecular weight (g/mol) | 194.2 |  |  |
| Log P | -0.15 | ADMET predictor | L1 |
|  | -0.07 | Measured (Corwin. Hansch et al., 1995) | L2,3 |
| Water solubility (mg/mL) | 13.92 | ADMET predictor at pH 7.56 | L1 |
|  | 0.988 | Measured in phosphate buffer (pH 7.4) (Moxon et al., 2020) | L2,3 |
| pKa | Base 2.24 | ADMET predictor | L1,2,3 |
| Unbound fraction in plasma ($f_{up}$) | 0.83 | ADMET predictor | L1 |
|  | >0.9 | Measured (Moxon et al., 2020) | L2 |
|  | 0.8 | Calibrated against clinical data (Blanchard & Sawers, 1983) | L3 |
| Blood: plasma ratio | 1.08 | ADMET predictor | L1 |
|  | 0.8 | Measured (Moxon et al., 2020) | L2,3 |
| Hepatic intrinsic clearance (L/h) | 67.4 | ADMET predictor with total HLM | L1 |
|  | 1.37 | based on in vitro CLint value 0.13±0.03 ml/min/109 cells using hepatocyte (Shibata et al., 2002) | L2 |
|  | 9 | Calibrated against clinical data (Blanchard & Sawers, 1983) | L3 |
| ECCS classification | Class_1A (Metabolism) | ADMET predictor |  |
| Renal excretion | 0 |  | L1,2,3 |
| Intestinal absorption: effective permeability | 4.01E10-4 | ADMET Predicter | L1,2,3 |
| Vehicle/Water partition coefficient | 1 | GastroPlus suggested default value | L1,2,3 |
| Stratum corneum/water partition coefficient | 4.1 | GastroPlus default value (WKN) | L1 |
|  | 1.96 | (Moxon et al., 2020) | L2,3 |
| Stratum corneum diffusivity (cm^2^/s) | 1.6E-12 | GastroPlus default value (WKN) | L1 |
|  | 1.44E-9 | (Moxon et al., 2020) | L2,3 |
| Epidermis/water partition coefficient | 0.7 | GastroPlus default value (Kretsos) | L1 |
|  | 0.8 | (Moxon et al., 2020) | L2,3 |
| Epidermis diffusivity (cm^2^/s) | 2.2E-06 | GastroPlus default value (Kretsos) | L1 |
|  | 6.67E-9 | (Moxon et al., 2020) | L2,3 |
| Dermis/water partition coefficient | 0.7 | GastroPlus default value (Kretsos) | L1 |
|  | 1.04 | (Moxon et al., 2020) | L2,3 |
| Dermis diffusivity (cm^2^/s) | 2.2E-06 | GastroPlus default value (Kretsos) | L1 |
|  | 3.06E-6 | (Moxon et al., 2020) | L2,3 |

#### Coumarin

|  | **Value** | **Source** | **Level** |
| --- | --- | --- | --- |
| Molecular weight (g/mol) | 147.1 |  |  |
| Log P | 1.89 | ADMET predictor | L1 |
|  | 1.39 | Measured (Corwin. Hansch et al., 1995) | L2,3 |
| Water solubility (mg/ml) | 0.37 | ADMET predictor at pH 7 | L1 |
|  | 0.96 | Measured in phosphate buffer (pH 7.4) (Moxon et al., 2020) | L2,3 |
| Unbound fraction in plasma ($f_{up}$) | 0.24 | ADMET predictor | L1 |
|  | 0.31 | Measured (Moxon et al., 2020) | L2,3 |
| Blood: plasma ratio | 1.08 | ADMET predictor | L1 |
|  | 0.7 | Measured (Moxon et al., 2020) | L2,3 |
| Hepatic intrinsic clearance (L/h) | 105 | ADMET predictor with total HLM | L1 |
|  | 929 | Measured; 105 µL/min/million cells (half-life 13 min) in human cryopreserved hepatocytes, (Moxon et al., 2020) | L2 |
|  | 3000 | Calibrated against clinical data (Ritschel & Grummich, 1981) | L3 |
| Ionization | Neutral | ADMET Predictor | L1,2,3 |
| ECCS classification | Class_2 (Metabolism) |  |  |
| Renal excretion | 0 |  | L1,2,3 |
| Intestinal absorption: effective permeability | 6.39E10-4 | ADMET Predicter | L1,2,3 |
| Stratum corneum/water partition coefficient | 14.9 | GastroPlus default value (WKN) | L1 |
|  | 8 | (Moxon et al., 2020) | L2,3 |
| Stratum corneum diffusivity (cm^2^/s) | 5.283E-11 | GastroPlus default value (WKN) | L1 |
|  | 3.0E-10 | (Moxon et al., 2020) | L2,3 |
| Epidermis/water partition coefficient | 0.7 | GastroPlus default value (Kretsos) | L1 |
|  | 0.7 | (Moxon et al., 2020) | L2,3 |
| Epidermis diffusivity (cm^2^/s) | 2.7E-6 | GastroPlus default value (Kretsos) | L1 |
|  | 2.7E-6 | (Moxon et al., 2020) | L2,3 |
| Dermis/water partition coefficient | 0.7 | GastroPlus default value (Kretsos) | L1 |
|  | 0.7 | (Moxon et al., 2020) | L2,3 |
| Dermis diffusivity (cm^2^/s) | 2.7E-6 | GastroPlus default value (Kretsos) | L1 |
|  | 2.7E-6 | (Moxon et al., 2020) | L2,3 |

#### Valproic acid

|  | **Value** | **Source** | **Level** |
| --- | --- | --- | --- |
| Molecular weight (g/mol) | 144.2 |  |  |
| Log P | 2.61 | ADMET predictor | L1 |
|  | 2.75 | Measured  (Sangster, 1997) | L2,3 |
| Water solubility (mg/mL) | 3.29 | ADMET predictor at pH3.28 | L1 |
|  | 2 | Measured In water, at 20 °C (Kubitschke, 2014) | L2,3 |
| pKa | Acid 4.92 | ADMET predictor | L1 |
|  | Acid 4.6 | (Williams, 2013) | L2,3 |
| Unbound fraction in plasma ($f_{up}$) | 0.12 | ADMET predictor | L1 |
|  | 0.1 | Measured  (Cramer & Mattson, 1979) | L2,3 |
| Blood: plasma ratio | 0.67 | ADMET predictor | L1,2,3 |
| Hepatic intrinsic clearance (L/h) | 40.43 | ADMET predictor with total HLM | L1 |
|  | 52.99 | Measured in human pooled microsomes with in vitro CLint value 13.9 ul/min/mg, respectively. (Argikar & Remmel, 2009) | L2 |
|  | 5.5 | Calibrated against clinical data (Perucca et al., 1978) | L3 |
| ECCS classification | Class_1A (Metabolism) | ADMET predictor |  |
| Renal excretion | 0 |  | L1,2,3 |
| Intestinal absorption: effective permeability | 5.63E-4 | ADMET Predicter | L1 |
|  | 3.4E-4 | Calculated based on measured Papp values of 2.26E-5 cm/s (Torii et al., 2002) using COVCa method in GastroPlus | L2,3 |

#### Paraquat dichloride

|  | **Value** | **Source** | **Level** |
| --- | --- | --- | --- |
| Molecular weight (g/mol) | 186.26 |  |  |
| Log P | -6.29 | ADMET predictor | L1 |
|  | -4.22 | Measured (Platford, 1983) | L2 |
| Water solubility (mg/mL) | 321 | ADMET predictor at pH 7 | L1 |
|  | 620 | Measured in water at 20 °C (MacBean & Council, 2012) | L2 |
| Ionization | Neutral | ADMET Predictor | L1,2 |
| Unbound fraction in plasma ($f_{up}$) | 0.998 | ADMET predictor | L1,2 |
| Blood: plasma ratio | 0.96 | ADMET predictor | L1,2 |
| Hepatic intrinsic clearance (L/h) | 23.39 | ADMET predictor with total HLM | L1,2 |
| ECCS classification | Class_4 (Renal) | ADMET predictor |  |
| Renal excretion | GFR*Fup |  | L1,2 |
| Intestinal absorption: effective permeability | 4.42E-4 | ADMET Predicter | L1,2 |

#### Butylated Hydroxytoluene (BHT)

|  | **Value** | **Source** | **Level** |
| --- | --- | --- | --- |
| Molecular weight (g/mol) | 220.36 |  |  |
| Log P | 5.53 | ADMET predictor | L1 |
|  | 5.1 | Measured (Chemicals., Oct 26, 2016) | L2,3 |
| Water solubility (mg/mL) | 0.017 | ADMET predictor at pH 6.99 | L1 |
|  | 0.6 | Measured In water at 25 °C Pubchem | L2 |
|  | 1.2E-3 | Calibrated against clinical data (Verhagen et al., 1989) | L3 |
| pKa | acid 11.41 | ADMET predictor | L1,2,3 |
| Unbound fraction in plasma ($f_{up}$) | 0.08 | ADMET predictor | L1 |
|  | 0.004 | Measured Pharmacelsus | L2 |
|  | 0.006 | Calibrated against clinical data (Verhagen et al., 1989) | L3 |
| Blood: plasma ratio | 1.05 | ADMET predictor | L1 |
|  | 0.83 | Measured Pharmacelsus | L2,3 |
| Hepatic intrinsic clearance (L/h) | 1340.2 | ADMET predictor with total HLM | L1,2 |
|  | 9103 | Calibrated against clinical data (Verhagen et al., 1989) | L3 |
| ECCS classification | Class_2 (Metabolism) | ADMET predictor |  |
| Renal excretion | 0 |  | L1,2,3 |
| Stratum corneum/water partition coefficient | 1332.7 | GastroPlus default value (WKN) | L1 |
|  | 1500 | Fitted against skin pen data Pharmacelsus | L2,3 |
| Stratum corneum diffusivity (cm^2^/s) | 8.88E-11 | GastroPlus default value (WKN) | L1 |
|  | 1E-10 | Fitted against skin pen data Pharmacelsus | L2,3 |
| Epidermis/water partition coefficient | 0.69994 | GastroPlus default value (Kretsos) | L1 |
|  | 200 | Fitted against skin pen data Pharmacelsus | L2,3 |
| Epidermis diffusivity (cm^2^/s) | 2.07E-06 | GastroPlus default value (Kretsos) | L1 |
|  | 5.00E-08 | Fitted against skin pen data Pharmacelsus | L2,3 |
| Dermis/water partition coefficient | 0.69994 | GastroPlus default value (Kretsos) | L1 |
|  | 20 | Fitted against skin pen data Pharmacelsus | L2,3 |
| Dermis diffusivity (cm^2^/s) | 2.07E-06 | GastroPlus default value (Kretsos) | L1 |
|  | 2.00E-08 | Fitted against skin pen data Pharmacelsus | L2,3 |

#### Oxybenzone

|  | **Value** | **Source** | **Level** |
| --- | --- | --- | --- |
| Molecular weight (g/mol) | 228.25 |  |  |
| Log P | 3.62 | ADMET predictor | L1 |
|  | 3.79 | Measured, Pubchem | L2,3 |
| Water solubility (mg/mL) | 0.0714 | ADMET predictor at pH 6.73 | L1 |
|  | 3.7 | Measured in water at 25 °C (European Commission, July 25, 2017) | L2,3 |
| pKa | Acid 10.11 | ADMET predictor | L1 |
|  | 7.1 (phenol) (est) | (ChemAxon., July 24, 2017) | L2,3 |
| Unbound fraction in plasma ($f_{up}$) | 0.054 | ADMET predictor | L1,2,3 |
| Blood: plasma ratio | 1.07 | ADMET predictor | L1,2,3 |
| Hepatic intrinsic clearance (L/h) | 169 | ADMET predictor with total HLM | L1,2,3 |
| ECCS classification | Class_2 (Metabolism) | ADMET predictor |  |
| Renal excretion | 0 |  | L1,2,3 |
| Vehicle/Water partition coefficient | 1 | GastroPlus suggested default value | L1 |
|  | 50 | Fitted against skin pen data (Hayden et al., 2005) | L2 |
|  | 800 | Calibrated against clinical data (Matta et al., 2020) | L3 |
| Stratum corneum/water partition coefficient | 73.09 | GastroPlus default value (WKN) | L1 |
|  | 5 | Fitted against skin pen data (Hayden et al., 2005) | L2,3 |
| Stratum corneum diffusivity (cm^2^/s) | 5.76E-11 | GastroPlus default value (WKN) | L1 |
|  | 3.00E-10 | Fitted against skin pen data (Hayden et al., 2005) | L2,3 |
| Epidermis/water partition coefficient | 0.69864 | GastroPlus default value (Kretsos) | L1 |
|  | 3 | Fitted against skin pen data (Hayden et al., 2005) | L2,3 |
| Epidermis diffusivity (cm^2^/s) | 2.02E-06 | GastroPlus default value (Kretsos) | L1 |
|  | 1.00E-09 | Fitted against skin pen data (Hayden et al., 2005) | L2,3 |
| Dermis/water partition coefficient | 0.69864 | GastroPlus default value (Kretsos) | L1 |
|  | 4 | Fitted against skin pen data (Hayden et al., 2005) | L2,3 |
| Dermis diffusivity (cm^2^/s) | 2.02E-06 | GastroPlus default value (Kretsos) | L1 |
|  | 1.00E-07 | Fitted against skin pen data (Hayden et al., 2005) | L2,3 |

#### 4-Hexylresorcinol

|  | **Value** | **Source** | **Level** |
| --- | --- | --- | --- |
| Molecular weight (g/mol) | 194.28 |  |  |
| Log P | 3.79 | ADMET predictor | L1 |
|  | 3.45 | Measured (Corwin. Hansch et al., 1995) | L2,3 |
| Water solubility (mg/mL) | 0.31 | ADMET predictor at pH 6.17 | L1 |
|  | 0.5 | Measured in water at 18°C (Yalkowsky, 2003) | L2,3 |
| pKa | acid 10.58, acid 9.57 | ADMET predictor | L1,2,3 |
| Unbound fraction in plasma ($f_{up}$) | 0.089 | ADMET predictor | L1,2,3 |
| Blood: plasma ratio | 1.25 | ADMET predictor | L1,2,3 |
| Hepatic intrinsic clearance (L/h) | 294.9 | ADMET predictor with total HLM | L1,2,3 |
| ECCS classification | Class_2 (Metabolism) | ADMET predictor |  |
| Renal excretion | 0 |  | L1,2,3 |
| Intestinal absorption: effective permeability | 5.29E-4 | ADMET Predicter | L1,2,3 |
| Stratum corneum/water partition coefficient | 90.15 | GastroPlus default value (WKN) | L1 |
|  | 45 | fitted against skin pen | L2,3 |
| Stratum corneum diffusivity (cm^2^/s) | 1.13E-10 | GastroPlus default value (WKN) | L1,2,3 |
| Epidermis/water partition coefficient | 0.695 | GastroPlus default value (Kretsos) | L1 |
|  | 40 | fitted against skin pen | L2,3 |
| Epidermis diffusivity (cm2/s) | 2.24E-06 | GastroPlus default value (Kretsos) | L1 |
|  | 5.00E-09 | fitted against skin pen | L2,3 |
| Dermis/water partition coefficient | 0.695 | GastroPlus default value (Kretsos) | L1 |
|  | 14 | fitted against skin pen | L2,3 |
| Dermis diffusivity (cm^2^/s) | 2.24E-06 | GastroPlus default value (Kretsos) | L1 |
|  | 1.00E-07 | fitted against skin pen | L2,3 |

#### Sulforaphane

|  | **Value** | **Source** | **Level** |
| --- | --- | --- | --- |
| Molecular weight (g/mol) | 177.3 |  |  |
| Log P | 1.22 | ADMET predictor | L1,2 |
| Water solubility (mg/mL) | 5.09 | ADMET predictor at pH 7.56 | L1 |
|  | 0.6 | Measured in phosphate buffer (pH 7.4) (Moxon et al., 2020) | L2 |
| pKa | 5.34 | ADMET predictor | L1,2 |
| Unbound fraction in plasma ($f_{up}$) | 0.61 | ADMET predictor | L1 |
|  | >0.95 | Measure (Moxon et al., 2020) | L2 |
| Blood: plasma ratio | 1.45 | ADMET predictor | L1,2 |
| Hepatic intrinsic clearance (L/h) | 60.03L/h | ADMET predictor with total HLM | L1,2 |
| ECCS classification | Class_2 (Metabolism) | ADMET predictor |  |
| Renal excretion | 0 |  | L1,2 |
| Intestinal absorption: effective permeability | 7.98E-4 | ADMET Predicter | L1,2,3 |

#### Rosiglitazone

|  | **Value** | **Source** | **Level** |
| --- | --- | --- | --- |
| Molecular weight (g/mol) | 357.4 |  |  |
| Log P | 2.59 | ADMET predictor | L1 |
|  | 3.19 | Measured  (EPA, 2018) | L2,3 |
| Water solubility (mg/mL) | 0.0518 | ADMET predictor at pH 7.56 | L1 |
|  | 0.01045 | Measured  (EPA, 2018) | L2,3 |
| pKa | 6.8, 6.1 | Pubchem | L1,2,3 |
| Unbound fraction in plasma ($f_{up}$) | 0.0548 | ADMET predictor | L1 |
|  | 0.00389 | Measure (Lin et al., 2004) | L2 |
|  | 0.0016 | Calibrated against clinical data (Chapelsky et al., 2003) | L3 |
| Blood: plasma ratio | 0.67 | ADMET predictor | L1,2 |
|  | 0.63 | Calibrated against clinical data (Chapelsky et al., 2003) (Moxon et al., 2020) | L3 |
| Hepatic intrinsic clearance (L/h) | 65.64L/h | ADMET predictor with total HLM | L1 |
|  | 1806L/h | Calibrated against clinical data (Chapelsky et al., 2003) | L3 |
| Vmax (nmol/min/mg protein) | 1.64 | (Bazargan et al., 2017) | L2 |
| Km (uM) | 25 |  |  |
| ECCS classification | Class_1A (Metabolism) | ADMET predictor |  |
| Renal excretion | 0 |  | L1,2,3 |
| Intestinal absorption: effective permeability | 3.39 | ADMET Predicter | L1,2,3 |

#### Niacinamide

|  | **Value** | **Source** | **Level** |
| --- | --- | --- | --- |
| Molecular weight (g/mol) | 122.12 |  |  |
| Log P | -0.33 | ADMET predictor | L1 |
|  | -0.37 | Measured (Corwin. Hansch et al., 1995) | L2,3 |
| Water solubility (mg/mL) | 50.57 | ADMET predictor at pH 7.56 | L1 |
|  | 500 | Measured in phosphate buffer at 25°C  MERCK INDEX (1996) | L2,3 |
| pKa | Base 3.79  Acid 12.12 | ADMET predictor | L1,2,3 |
| Unbound fraction in plasma ($f_{up}$) | 0.82 | ADMET predictor | L1,2,3 |
| Blood: plasma ratio | 1.27 | ADMET predictor | L1,2,3 |
| Hepatic intrinsic clearance (L/h) | 116.7L/h | ADMET predictor with total HLM | L1 |
| Vmax (pmol/min/mg protein) | 60.14 | (Real et al., 2013) | L2,3 |
| Km (uM) | 2.98 |  |  |
| ECCS classification | Class_1A (Metabolism) | ADMET predictor |  |
| Renal excretion | 0 |  | L1,2,3 |
| Intestinal absorption: effective permeability | 2.34 | ADMET Predicter | L1,2,3 |
| Stratum corneum/water partition coefficient | 1.9144 | GastroPlus default value (WKN) | L1,2 |
|  | 1 | Calibrated against clinical data (Feldmann & Maibach, 1970) | L3 |
| Stratum corneum diffusivity (cm^2^/s) | 1.111E-10 | GastroPlus default value (WKN) | L1,2 |
|  | 2.00E-11 | Calibrated against clinical data (Feldmann & Maibach, 1970) | L3 |
| Epidermis/water partition coefficient | 0.7 | GastroPlus default value (Kretsos) | L1,2,3 |
| Epidermis diffusivity (cm^2^/s) | 3.042E-6 | GastroPlus default value (Kretsos) | L1,2 |
|  | 3.04E-08 | Calibrated against clinical data (Feldmann & Maibach, 1970) | L3 |
| Dermis/water partition coefficient | 0.7 | GastroPlus default value (Kretsos) | L1,2,3 |
| Dermis diffusivity (cm^2^/s) | 3.042E-6 | GastroPlus default value (Kretsos) | L1,2,3 |

#### Doxorubicin

|  | **Value** | **Source** | **Level** |
| --- | --- | --- | --- |
| Molecular weight (g/mol) | 543.5 |  |  |
| Log P | 0.49 | ADMET predictor | L1 |
|  | 1.27 | Measure (C. Hansch et al., 1995) | L2,3 |
| Water solubility (mg/mL) | 0.61 | ADMET predictor at pH 7.56 | L1,2,3 |
| pKa | Acid 9.5, 6.7  Base 8.43 | ADMET predictor | L1 |
|  | 7.34 (phenol); 8.46 (amine); 9.46 (est) | (SPARC, 2008) | L2,3 |
| Unbound fraction in plasma ($f_{up}$) | 0.28 | ADMET predictor | L1,2 |
|  | 0.25 | (Chassany et al., 1996) (Ryu et al., 2014) | L3 |
| Blood: plasma ratio | 1.09 | ADMET predictor | L1,2 |
|  | 1.72 | Value converted from the measured erythrocyte/plasma concentration ratio of 2.8±0.3 for DOX (Skorokhod et al., 2007) | L3 |
| Hepatic intrinsic clearance (L/h) | 12.37 | ADMET predictor with total HLM | L1,2 |
|  | 4455 | (Yoshida et al., 1994) | L3 |
| ECCS classification | Class_2 (Metabolism) | ADMET predictor |  |
| Renal excretion | 0 |  | L1,2,3 |

## T2. PBK Cmax estimates

Summary of C_max_ values (estimated and measured) on which the Cmax Error Distribution (CMED) model was trained (N/A indicates that a value was not available, e.g. due to a lack of suitable clinical data). a: (Otberg et al., 2008); b: (Ford et al., 2001) c: (Seth, 1992); d: (Kochak et al., 1992) e: (Davis et al., 1997).

| **Chemical** | **Use scenario** | **Route** | **PBK L1 Cmax (μM)** | **PBK L2 Cmax (μM)** | **PBK L3 Cmax (μM)** | **Measured Cmax (μM)** |
| --- | --- | --- | --- | --- | --- | --- |
| Butylated hydroxytoluene | Body Lotion, 0.5% | Dermal | 0.16 | 0.15 | 0.036 | N/A |
| Caffeine | Shampoo, 0.2% | Dermal | 0.0040 | 0.044 | 0.019 | N/A |
| Caffeine | 2 mg/cm^2^, 25 cm^2^ | Dermal | 0.0020 | 0.13 | 0.12 | 0.062^a^ |
| Caffeine | Food & Drink, 400 mg/day | Oral | 11 | 83 | 30 | 52 |
| Caffeine | Overdose | Oral | 278 | 1151 | 745 | 1200 |
| Coumarin | Food, 4.1 mg/day | Oral | 0.31 | 0.037 | 0.011 | N/A |
| Coumarin | Food, 0.1 mg/kg bw/day | Oral | 0.60 | 0.073 | 0.020 | 0.010 |
| Coumarin | Body Lotion, 0.38% | Dermal | 1.6 | 1.2 | 0.75 | N/A |
| Coumarin | 0.02 mg/cm^2^, 100 cm^2^ | Dermal | 0.072 | 0.13 | 0.12 | 0.24^b^ |
| Diclofenac | 300 mg, 400 cm^2^ | Dermal | 4.5 | 0.44 | 0.21 | 0.15­^c^ |
| Doxorubicin | 75 mg/m^2^/day for 10 minutes | Intravenous | 6.4 | 6.3 | 10 | N/A |
| Doxorubicin | 4.5 mg/m^2^/day continuous infusion for four days | Intravenous | 0.11 | 0.11 | 0.012 | N/A |
| Hexylresorcinol | Food residues, 0.0033 mg/kg bw/day | Oral | 0.0070 | 0.006 | N/A | N/A |
| Hexylresorcinol | Face Serum, 0.5% | Dermal | 0.0893 | 0.086 | N/A | N/A |
| Hexylresorcinol | Throat Lozenge, 2.4 mg | Oral | 0.17 | 0.20 | N/A | N/A |
| Niacinamide | Hair Conditioner, 0.1% | Dermal | 0.0020 | 0.006 | 0.0040 | N/A |
| Niacinamide | Food & Drink, 22.2 mg/day | Oral | 0.84 | 3.8 | 3.8 | N/A |
| Niacinamide | Body Lotion, 3% | Dermal | 10 | 34 | 9.8 | N/A |
| Niacinamide | Food & Drink, 12.5 mg/kg bw/day | Oral | 33 | 148 | 148 | 163 |
| Nicotine | 30 cm^2^ patch, 0.7 mg/cm^2^/24h | Dermal | 0.072 | 0.31 | 0.084 | 0.078^d^ |
| Oxybenzone | Body Lotion, 0.5% | Dermal | 1.1 | 1.0 | 0.52 | N/A |
| Oxybenzone | Sunscreen, 2% | Dermal | 8.8 | 6.2 | 2.7 | N/A |
| Paraquat dichloride | Pesticide poisoning, 35 mg/kg/day | Oral | 191 | 191 | N/A | N/A |
| Rosiglitazone | Medical, 8 mg | Oral | 0.59 | 1.0 | 0.86 | 1 |
| Rosiglitazone | Medical, 1 mg/12 hours | Oral | 0.097 | 0.21 | 0.13 | N/A |
| Salicylic acid | 27 mg over face and neck | Dermal | 4.9 | 3.8 | 4.6 | 2.0^e^ |
| Sulforaphane | Food & Drink, 3.9 mg/day | Oral | 0.23 | 0.15 | N/A | 0.070 |
| Sulforaphane | Tablet, 60 mg/day | Oral | 1.2 | 0.77 | N/A | N/A |
| Valproic acid | Tablet, 1000 mg | Oral | 398 | 360 | 856 | N/A |
| Valproic acid | Tablet, 60 mg/kg/day | Oral | 1431 | 1335 | 3080 | N/A |

## T3. Cell Stress Panel: Biomarkers and Reagents

| Assay Plate | Biomarker | Pathway | Measurement method | Primary Antibody/Cellular dye/Assay Kit | | Supplier information | | Positive controls on assay plate |
| --- | --- | --- | --- | --- | --- | --- | --- | --- |
| **HCS GSH/ROS/MMP/ATP Assay** | | | | | | | | |
| 1 | Glutathione Content (GSH) | Oxidative Stress | High Content Imaging | Monochlorobimane (mBCL) | | Sigma-Aldrich (Dorset, UK) | | Rotenone, L-Buthionine Sulfoximine |
|  | Oxidative Stress (ROS) | Oxidative Stress | High Content Imaging | Dihydroethidium (DHE) | | Sigma-Aldrich (Dorset, UK) | |  |
|  | Mitochondrial Membrane Potential (MMP) | Mitochondrial Toxicity | High Content Imaging | MitoTracker™ Deep Red FM | | ThermoFisher Scientific (Loughborough, UK) | |  |
|  | Mitochondrial Mass (Mito-Mass) | Mitochondrial Toxicity | High Content Imaging | MitoTracker™ Deep Red FM | | ThermoFisher Scientific (Loughborough, UK) | |  |
|  | Cellular ATP | Mitochondrial Toxicity | Luminescence Plate Reader | CellTiter-Glo® Luminescent Cell Viability Assay | | Promega (Maddison, WI, US) | |  |
|  | Nuclei (Cell Count/Nuclear Area/DNA Structure) | Cell Health & Physiology | High Content Imaging | SYTO 11 Green Fluorescent Nucleic Acid Stain | | ThermoFisher Scientific (Loughborough, UK) | |  |
| **LDH Assay** | | | | | | | | |
| 2 | LDH release (supernatant) | Cell Health & Physiology | Absorbance Plate Reader | CytoTox 96® Non-Radioactive Cytotoxicity Assay | Promega (Maddison, WI, US) | | Rotenone, L-Buthionine Sulfoximine | |
| **Mitochondrial Oxidative Stress Assay (PCG1alpha)** | | | | | | | | |
| 3 | PGC1alpha | Mitochondrial Toxicity | High Content Imaging | Rabbit anti-PGC1 alpha antibody (ab54481) | | Abcam (Cambridge, UK) | | Rotenone, Rosiglitazone |
|  | Mitochondrial ROS (MitoROS) | Mitochondrial Toxicity | High Content Imaging | MitoSOX™ Red Mitochondrial Superoxide Indicator | | ThermoFisher Scientific (Loughborough, UK) | |  |
|  | Nuclei (Cell Count/Nuclear Area/DNA Structure) | Cell Health & Physiology | High Content Imaging | Hoechst 33342, Trihydrochloride, Trihydrate | | ThermoFisher Scientific (Loughborough, UK) | |  |
| **Oxidative Stress Assay (Heme oxygenase 1 and NRF2)** | | | | | | | | |
| 4 | NRF2 | Oxidative Stress | High Content Imaging | Anti-Nrf2 antibody (ab89443) (Mouse) | | Abcam (Cambridge, UK) | | Carbamazepine, Ethacrynic acid |
|  | Heme Oxygenase 1 (HMOX1) | Oxidative Stress | High Content Imaging | Recombinant Anti-Heme Oxygenase 1 antibody [EPR18161-128] (ab189491) (Rabbit) | | Abcam (Cambridge, UK) | |  |
|  | Nuclei (Cell Count/Nuclear Area/DNA Structure) | Cell Health & Physiology | High Content Imaging | Hoechst 33342, Trihydrochloride, Trihydrate | | ThermoFisher Scientific (Loughborough, UK) | |  |
| **Oxidative Stress Assay (SRXN1 only)** | | | | | | | | |
| 5 | SRXN1 | Oxidative Stress | High Content Imaging | Anti-SRXN1 (SAB2501200) (Goat) | Sigma-Aldrich (Dorset, UK) | | Carbamazepine, Ethacrynic acid | |
|  | Nuclei (Cell Count/Nuclear Area/DNA Structure) | Cell Health & Physiology | High Content Imaging | Hoechst 33342, Trihydrochloride, Trihydrate | ThermoFisher Scientific (Loughborough, UK) | |  |  |
| **DNA Damage Assay** | | | | | | | | |
| 6 | DNA Damage (p-H2AX) | DNA Damage | High Content Imaging | Anti-phospho-Histone H2A.X (Ser139), clone JBW301 Monoclonal Antibody (05-636) (Mouse) | | Merck-Millipore (Darmstadt, Germany) | | Chlorambucil, (S)-(+)-camptothecin |
|  | Phospho-p53 | DNA Damage | High Content Imaging | Anti-p53 (phosho S15) (ab38497) (Rabbit) | | Abcam (Cambridge, UK) | |  |
|  | Nuclei (Cell Count/Nuclear Area/DNA Structure) | Cell Health & Physiology | High Content Imaging | Hoechst 33342, Trihydrochloride, Trihydrate | | ThermoFisher Scientific (Loughborough, UK) | |  |
| **Phospholipidosis & Steatosis Assay** | | | | | | | | |
| 7 | Phospholipidosis (PLD) | Cell Health & Physiology | High Content Imaging | HCS LipidTOX™ Red Phospholipidosis Detection Reagent | | ThermoFisher Scientific (Loughborough, UK) | | Sertraline, Cyclosporine A |
|  | Steatosis | Cell Health & Physiology | High Content Imaging | HCS LipidTOX™ Green Neutral Lipid Stain | | ThermoFisher Scientific (Loughborough, UK) | |  |
|  | Nuclei (Cell Count/Nuclear Area/DNA Structure) | Cell Health & Physiology | High Content Imaging | Hoechst 33342, Trihydrochloride, Trihydrate | | ThermoFisher Scientific (Loughborough, UK) | |  |
| **ER Stress 2 Assay** | | | | | | | | |
| 8 | PERK | ER Stress | High Content Imaging | Anti-PERK antibody (ab65142) (Rabbit) | | Abcam (Cambridge, UK) | | Sertraline, Tunicamycin |
|  | ATF4 | ER Stress | High Content Imaging | Anti-ATF-4 antibody - C-terminal (ab1371) (Goat) | | Abcam (Cambridge, UK) | |  |
|  | CHOP | ER Stress | High Content Imaging | CHOP monoclonal antibody (9C8) Mouse IgG2B (MA1-250) | | ThermoFisher Scientific (Loughborough, UK) | |  |
|  | Nuclei (Cell Count/Nuclear Area/DNA Structure) | Cell Health & Physiology | High Content Imaging | SYTO 11 Green Fluorescent Nucleic Acid Stain | | ThermoFisher Scientific (Loughborough, UK) | |  |
| **ER Stress 1 Assay** | | | | | | | | |
| 9 | Endoplasmic Reticulum (ER) | ER Stress | High Content Imaging | ER-Tracker™ Blue-White DPX | | ThermoFisher Scientific (Loughborough, UK) | | Sertraline, Tunicamycin |
|  | BiP | ER Stress | High Content Imaging | Anti-GRP78 BiP antibody (ab21685) (Rabbit) | | Abcam (Cambridge, UK) | |  |
|  | Nuclei (Cell Count/Nuclear Area/DNA Structure) | Cell Health & Physiology | High Content Imaging | SYTO 11 Green Fluorescent Nucleic Acid Stain | | ThermoFisher Scientific (Loughborough, UK) | |  |
| **Osmotic & Heat Shock Stress Assay** | | | | | | | | |
| 10 | Heat Shock Response (Hsp70)* | Heat Shock Response | High Content Imaging | Anti-Hsp70 antibody [2A4] (ab5442) (Mouse) | | Abcam (Cambridge, UK) | | Sodium Chloride, Sorbitol |
|  | NFAT | Osmotic Stress | High Content Imaging | Anti NFAT5 (PA1-023) (Rabbit) | | ThermoFisher Scientific (Loughborough, UK) | |  |
|  | Nuclei (Cell Count/Nuclear Area/DNA Structure) | Cell Health & Physiology | High Content Imaging | Hoechst 33342, Trihydrochloride, Trihydrate | | ThermoFisher Scientific (Loughborough, UK) | |  |
| **Metal Stress Assay** | | | | | | | | |
| 11 | MTF1 | Metal Stress | High Content Imaging | Rabbit anti-MTF1 antibody (ab183897) (Rabbit) | | Abcam (Cambridge, UK) | | Cadmium (II) chloride, Sodium arsenite |
|  | Metallothionein (MT) | Metal Stress | High Content Imaging | Anti-Metallothionein antibody [UC1MT] (ab12228) (Mouse) | | Abcam (Cambridge, UK) | |  |
|  | Nuclei (Cell Count/Nuclear Area/DNA Structure) | Cell Health & Physiology | High Content Imaging | Hoechst 33342, Trihydrochloride, Trihydrate | | ThermoFisher Scientific (Loughborough, UK) | |  |
| **Inflammation, Hypoxia & pH Assay** | | | | | | | | |
| 12 | HIF1alpha | Hypoxia | High Content Imaging | Anti-HIF-1 alpha antibody [H1alpha67] – ChIP Grade (ab1) (Mouse) | | Abcam (Cambridge, UK) | | Deferoxamine mesylate salt, pH media |
|  | Intracellular pH | Cell Health & Physiology | High Content Imaging | pHrodo Red AM | | ThermoFisher Scientific (Loughborough, UK) | |  |
|  | ICAM1 | Inflammation | High Content Imaging | Anti-ICAM1 antibody [EPR4776] (ab214944) (Rabbit) | | Abcam (Cambridge, UK) | |  |
|  | Nuclei (Cell Count/Nuclear Area/DNA Structure) | Cell Health & Physiology | High Content Imaging | Hoechst 33342, Trihydrochloride, Trihydrate | | ThermoFisher Scientific (Loughborough, UK) | |  |
| **Apoptosis, Necrosis & Cell Cycle Arrest Assay** | | | | | | | | |
| 13 | DNA content (measuring Cell Cycle Arrest) | Cell Health & Physiology | High Content Imaging | Hoechst 33342, Trihydrochloride, Trihydrate | | ThermoFisher Scientific (Loughborough, UK) | | Ionomycin, Staurosporine |
|  | Cell Membrane Permeability (Necrosis) | Cell Health & Physiology | High Content Imaging | TO-PRO™-3 Iodide (642/661) | | ThermoFisher Scientific (Loughborough, UK) | |  |
|  | Caspase 3/7 intensity (Apoptosis) | Cell Health & Physiology | High Content Imaging | CellEvent™ Caspase-3/7 Green Detection Reagent | | ThermoFisher Scientific (Loughborough, UK) | |  |
|  | Nuclei (Cell Count/Nuclear Area/DNA Structure) | Cell Health & Physiology | High Content Imaging | Hoechst 33342, Trihydrochloride, Trihydrate | | ThermoFisher Scientific (Loughborough, UK) | |  |
| **Inflammatory Response Assay** | | | | | | | | |
| 14 | IL-8 (supernatant) | Inflammation | ELISA | IL-8 Human ELISA Kit (KHC0081) | | ThermoFisher Scientific (Loughborough, UK) | | TNF alpha, Ionomycin |
| **AhR translocation Assay** | | | | | | | | |
| 15 | AhR Translocation | Aryl hydrocarbon receptor (AhR) | High Content Imaging | Anti Aryl Hydrocarbon Receptor (ab84833) (Rabbit) | | Abcam (Cambridge, UK) | | Benzo(a)pyrene, Leflunomide |
|  | Nuclei (Cell Count/Nuclear Area/DNA Structure) | Cell Health & Physiology | High Content Imaging | Hoechst 33342, Trihydrochloride, Trihydrate | | ThermoFisher Scientific (Loughborough, UK) | |  |
| **TMRE & ATP Assay** | | | | | | | | |
| 16 | Mitochondrial Membrane Potential (MMP) | Mitochondrial Toxicity | High Content Imaging | Tetramethylrhodamine, Ethyl Ester, Perchlorate (TMRE) | | ThermoFisher Scientific (Loughborough, UK) | |  |
|  | Mitochondrial Mass (Mito-Mass) | Mitochondrial Toxicity | High Content Imaging | Tetramethylrhodamine, Ethyl Ester, Perchlorate (TMRE) | | ThermoFisher Scientific (Loughborough, UK) | |  |
|  | Cellular ATP | Mitochondrial Toxicity | Luminescence Plate Reader | CellTiter-Glo® Luminescent Cell Viability Assay | | Promega (Maddison, WI, US) | |  |
|  | Nuclei (Cell Count/Nuclear Area/DNA Structure) | Cell Health & Physiology | High Content Imaging | Hoechst 33342, Trihydrochloride, Trihydrate | | ThermoFisher Scientific (Loughborough, UK) | |  |
| **Extracellular Flux (Seahorse) Assay** | | | | | | | | |
| 17 | Oxygen consumption rate (OCR) | Mitochondrial Toxicity | XF^e^96 Extracellular Flux Analyzer | XF^e^96 Flux Pack | | Seahorse Biosciences (North Billerica, MA, USA) | | Rotenone |
|  | Reserve Capacity | Mitochondrial Toxicity | XF^e^96 Extracellular Flux Analyzer | XF^e^96 Flux Pack | | Seahorse Biosciences (North Billerica, MA, USA) | |  |
|  | Extracellular Acidification Rate (ECAR) | Mitochondrial Toxicity | XF^e^96 Extracellular Flux Analyzer | XF^e^96 Flux Pack | | Seahorse Biosciences (North Billerica, MA, USA) | |  |
| **XBP1 Assay** | | | | | | | | |
| 18 | XBP1 | ER Stress | High Content Imaging | Anti XBP1 (ab37152) (Rabbit) | | Abcam (Cambridge, UK) | | Sertraline, Tunicamycin |
|  | Nuclei (Cell Count/Nuclear Area/DNA Structure) | Cell Health & Physiology | High Content Imaging | Hoechst 33342, Trihydrochloride, Trihydrate | | ThermoFisher Scientific (Loughborough, UK) | |  |

## T4. Cell Stress Panel and HTTr dosing

### Cell Stress Panel

Stock solutions of all compounds made up in DMSO and diluted down to give a final DMSO concentration of 0.5% in media.

| **Compound** | **Concentrations (µM)** |
| --- | --- |
| Butylated Hydroxytoluene | 0.0128, 0.04, 0.2, 0.78, 3.13, 12.5, 50, 200 |
| Caffeine* | 0.31, 1.22, 4.88, 19.53, 78.13, 312.5, 1250, 5000 |
| Coumarin | 0.06, 0.24, 0.98, 3.91, 15.63, 62.5, 250, 1000 |
| Doxorubicin | 0.000061, 0.00024, 0.00098, 0.0039, 0.016, 0.063, 0.25, 1 |
| 4-Hexylresorcinol | 0.0061, 0.024, 0.098, 0.39, 1.56, 6.25, 25, 100 |
| Niacinamide | 3.66, 14.65, 58.59, 937.5, 3750, 15000, 60000 |
| Oxybenzone | 0.012, 0.049, 0.20, 0.78, 3.13, 12.5, 50, 200 |
| Paraquat* | 0.0061, 0.024, 0.098, 0.39, 1.56, 6.25, 25, 100 |
| Rosiglitazone | 0.012, 0.049, 0.2, 0.78. 3.13, 12.5, 50, 200 |
| Sulforaphane | 0.0061, 0.024, 0.098, 0.39, 1.56, 6.25, 25, 100 |
| Valproic acid | 1.2, 4.9, 19.5, 78.1, 312.5, 1250, 5000, 20000 |

*Stock solution made in water with 0.5% DMSO spiked in at the cell treatment stage.

### HTTr

| **Compound** | **Cell Line** | **Concentrations (µM)** |
| --- | --- | --- |
| Butylated Hydroxytoluene | HepG2 | 0.0128, 0.064, 0.32, 1.6, 8, 40, 200 |
|  | HepaRG | 0.0128, 0.064, 0.32, 1.6, 8, 40, 200 |
|  | MCF7 | 0.0128, 0.064, 0.32, 1.6, 8, 40, 200 |
| Caffeine | HepG2 | 0.32, 1.6, 8, 40, 200, 1000, 5000 |
|  | HepaRG | 0.32, 1.6, 8, 40, 200, 1000, 5000 |
|  | MCF7 | 0.32, 1.6, 8, 40, 200, 1000, 5000 |
| Coumarin | HepG2 | 0.064, 0.32, 1.6, 8, 40, 200, 1000 |
|  | HepaRG | 0.064, 0.32, 1.6, 8, 40, 200, 1000 |
|  | MCF7 | 0.064, 0.32, 1.6, 8, 40, 200, 1000 |
| Doxorubicin | HepG2 | 0.000064, 0.00032, 0.0016, 0.008, 0.04, 0.2, 1 |
|  | HepaRG | 0.000064, 0.00032, 0.0016, 0.008, 0.04, 0.2, 1 |
|  | MCF7 | 0.000064, 0.00032, 0.0016, 0.008, 0.04, 0.2, 1 |
| 4-Hexylresorcinol | HepG2 | 0.0064, 0.032, 0.16, 0.8, 4, 20, 100 |
|  | HepaRG | 0.0064, 0.032, 0.16, 0.8, 4, 20, 100 |
|  | MCF7 | 0.0064, 0.032, 0.16, 0.8, 4, 20, 100 |
| Niacinamide | HepG2 | 3.84, 19.2, 96, 480, 2400, 12000, 60000 |
|  | HepaRG | 0.512, 2.56, 12.8, 64, 320, 1600, 8000 |
|  | MCF7 | 3.84, 19.2, 96, 480, 2400, 12000, 60000 |
| Oxybenzone | HepG2 | 0.0128, 0.064, 0.32, 1.6, 8, 40, 200 |
|  | HepaRG | 0.0128, 0.064, 0.32, 1.6, 8, 40, 200 |
|  | MCF7 | 0.0128, 0.064, 0.32, 1.6, 8, 40, 200 |
| Paraquat | HepG2 | 0.0064, 0.032, 0.16, 0.8, 4, 20, 100 |
|  | HepaRG | 0.192, 0.96, 4.8, 24, 120, 600, 3000 |
|  | MCF7 | 0.032, 0.16, 0.8, 40, 20, 100, 500 |
| Rosiglitazone | HepG2 | 0.0064, 0.032, 0.16, 0.8, 4, 20, 100 |
|  | HepaRG | 0.0128, 0.064, 0.32, 1.6, 8, 40, 200 |
|  | MCF7 | 0.0128, 0.064, 0.32, 1.6, 8, 40, 200 |
| Sulforaphane | HepG2 | 0.0064, 0.032, 0.16, 0.8, 4, 20, 100 |
|  | MCF7 | 0.0032, 0.016, 0.08, 0.4, 2, 10, 50 |
|  | HepaRG | 0.0128, 0.064, 0.32, 1.6, 8, 40, 200 |
| Valproic Acid | HepG2 | 1.28, 6.4, 32, 160, 800, 4000, 20000 |
|  | HepaRG | 1.28, 6.4, 32, 160, 800, 4000, 20000 |
|  | MCF7 | 1.28, 6.4, 32, 160, 800, 4000, 20000 |
| Trichostatin A (positive control) | HepG2 | 0.00128, 0.0064, 0.032, 0.16, 0.8, 4, 20 |
|  | HepaRG | 0.000128, 0.00064, 0.0032, 0.016, 0.08, 0.4, 2 |
|  | MCF7 | 0.0128, 0.064, 0.32, 1.6, 8, 40, 200 |
| Tunicamycin (positive control) | HepG2 | 0.0064, 0.032, 0.16, 0.8, 4, 20, 100 |
|  | HepaRG | 0.0064, 0.032, 0.16, 0.8, 4, 20, 100 |
|  | MCF7 | 0.0064, 0.032, 0.16, 0.8, 4, 20, 100 |

## T5. Cell information

### Cell Stress Panel

|  | **HepG2 cells** | **HepG2 cells Seahorse** | **Seahorse Media** |
| --- | --- | --- | --- |
| Supplier | ECACC | ECACC |  |
| Catalogue number | 85011430 | 85011430 |  |
| Batch | 17K028 | 17K028 |  |
| Media | EMEM, 10% FBS, 2mM Glutamax, 1x NEAA and 0.5% Pen/Strep | EMEM, 10% FBS, 2mM Glutamax, 1x NEAA and 0.5% Pen/Strep | DMEM (Sigma D5030) supplemented with 30mM NaCl, 2mM Glutamax, 10mM Glucose and 1mM Sodium Pyruvate |
| Culturing conditions | 37°C, 5%CO_2_, humidified  Sub-cultured twice a week | 37°C, 5%CO_2_, humidified  Sub-cultured twice a week |  |
| Seeding densities | 6,000 cells per well | 16,000 cells per well |  |
| Seeding volumes | 50uL | 80uL |  |
| Culture time in plates before dosing | 24hr | 24hr |  |
| Plate format | 384 | 96 |  |
| Plate manufacturer | Corning | Agilent |  |
| Plate catalogue number | 3764 | 102416-100 |  |

### HTTr cell lysate generation

|  | **HepG2 cells** | **MCF-7 cells** | **HepaRG cells** |
| --- | --- | --- | --- |
| Supplier | ECACC | ECACC | Life Technologies |
| Catalogue number | 85011430 | 86012803 | HPRGC10 |
| Batch | 17K028 | 17J001 | 2142421 |
| Media | EMEM, 10% FBS, 2mM Glutamax, 1x NEAA and 0.5% Pen/Strep | RPMI, 10% FBS, 2mM Glutamax and 0.5% Pen/Strep | Williams E containing HepaRG Thaw, Plate and General Purpose Medium Supplement, 2mM Ultraglutamine and 0.5% Pen/Strep |
| Culturing conditions | 37°C, 5%CO_2_, humidified  Sub-cultured twice a week | 37°C, 5%CO_2_, humidified  Sub-cultured twice a week | 37°C, 5%CO_2_, humidified  seeded directly into plates, media refreshed every 2 to 3 days |
| Seeding densities | 6,000 cells per well | 6,000 cells per well | 20,000 cells per well |
| Seeding volumes | 50uL | 50uL | 50uL |
| Culture time in plates before dosing | 24hr | 24hr | 168hr |
| Plate format | 384 | 384 | 384 |
| Plate manufacturer | Corning | Corning | Corning |
| Plate catalogue number | 3764 | 3764 | 356667 |

## T6. Summary of the PODs estimated for each bioactivity platform

Platform-specific PODs for each chemical. Separate PODs are provided for each high-throughput transcriptomics platform cell model (HepG2, HepaRG and MCF-7), with two for each POD estimation method (BMDexpress and BIFROST). The minimum POD used to calculate a BER for each chemical is indicated in bold.

| **Chemical** | **IPP lowest AC50** | **cell stress panel Global POD** | **high-throughput transcriptomics Global POD** | | | **Minimum pathway BMDL** | | | | |
| --- | --- | --- | --- | --- | --- | --- | --- | --- | --- | --- |
|  |  |  | **HepaRG** | **HepG2** | **MCF7** | **HepaRG** | | **HepG2** | | **MCF7** |
| Coumarin | 26 | 47 | 150 | 11 | **4.1** | 110 | 190 | | 190 | |
| Paraquat dichloride | 32 | 1.3 | **0.24** | 1.3 | 0.78 | 1.7 | 5.0 | | 0.70 | |
| Butylated hydroxytoluene | >10 | 6.5 | 3.4 | 4.5 | **1.6** | 200 | 6.1 | | 5.1 | |
| Caffeine | **5.3** | 61 | 220 | 33 | 14 | 1800 | 210 | | 140 | |
| Doxorubicin | 0.8 | **0.0011** | 0.0027 | 0.0012 | 0.0056 | 0.21 | 0.033 | | 0.021 | |
| Hexylresorcinol | **0.20** | 3.8 | 8.1 | 7.3 | 0.80 | 53 | 27 | | 15 | |
| Niacinamide | >100 | 1300 | 680 | 100 | **26** | 4500 | 11000 | | 4800 | |
| Oxybenzone | 2.8 | 9.2 | 4.9 | 6.3 | **0.65** | 92 | 36 | | 22 | |
| Rosiglitazone | 0.077 | 0.033 | 0.0044 | **0.0014** | 0.01 | 49 | 41 | | 28 | |
| Sulforaphane | >10 | 0.51 | 0.75 | **0.072** | 0.18 | 9.0 | 1.6 | | 1.9 | |

## T7. Summary of the bioactivity observed for each of the 11 chemicals and comparison to the known mechanism of action

An overview of the individual assay results for the IPP and CSP platforms are provided in the Supplementary Figures section, F2-F3.

| **Chemical** | **Bioactivity observed across the different platforms and comparison to in vitro and in vivo human effects reported in the literature** |
| --- | --- |
| Niacinamide | Niacinamide or vitamin B3 is as essential nutrient used in the synthesis of the NAD+ family of coenzymes, contributing to cellular energy metabolism and defence systems.  As a cosmetic ingredient it has been used as an anti-ageing and skin lightening ingredient. It has been suggested that both effects are related to its ROS/free radical-scavenging action and ability to restore the NAD+ pool and delay the senescence of cells. Both niacinamide and its metabolites exert anti-inflammatory properties in various experiments (Boo, 2021). As expected, Niacinamide was the least potent chemical in the dataset, causing cytotoxicity at high concentrations (>3000 µM) |
| Coumarin | MAO-A inhibition has been previously reported at similar IC50 20-40 µM) (Gnerre et al., 2000; Sipes et al., 2013) as observed here. Carbonic anhydrase II inhibition was not detected in this experiment (Maresca & Supuran, 2010; Maresca et al., 2009).  The observed GSH and oxidative stress biomarkers at high concentrations is consistent with the literature (Lake & Grasso, 1996);Hassanein, 2020 #36}. |
| Caffeine | Caffeine is an antagonist of the adenosine A2A receptor (and other adenosine receptors) at IC50s similar to reported here (Jacobson et al.).  Caffeine has also been reported to inhibit PDE1b, PDE2, PDE3, PDE4, PDE5,GABA_A_, MAO-A. However these activities were detected at concentrations > than 100 µM tested here (Jacobson et al.; Petzer et al., 2013; Pohanka, 2015) |
| Butylated hydroxytoluene (BHT) | BHT and its metabolites act as a radical-scavengers and are used as antioxidants in foods.  At high concentration BHT and its metabolites (e.g. quinone methide metabolites) can act as a prooxidants and increase oxidative stress (Additives & Food, 2012; Nieva-Echevarría et al., 2015), which was observed in the CSP. |
| Paraquat Dichloride (PQ) | All observed responses detected in the CSP are consistent with PQ mode of action. When PQ enters the cell, it goes through redox cycling in the presence of NADPH and oxygen leading to the generation of superoxide and hydroxyl radicals causing oxidative stress (M. T. Baltazar et al., 2014). PQ is known to inhibit complex I and complex III of the electron transport chain leading to an impairment of respiration, and consequently decline of ATP production (Huang et al., 2016).  PQ is also known to induce the translocation of NF-KB transcription factor and IL-8 is a downstream cytokine (Maria Teresa Baltazar et al., 2014).  Acetylcholinesterase inhibition has also been previously reported (Del Pino et al., 2017; El-Demerdash et al., 2001). |
| Oxybenzone/  Benzophenone-3 (BP-3) | Oxybenzone is a commonly used sunscreen agent, absorbing UVB and UVA radiation.  The SCCS considers that the overall evidence is not conclusive enough at present for the SCCS to ascertain whether or not BP-3 is an endocrine disruptor substance (SCCS, 2021). Here we found that oxybenzone did not have any effect on endocrine receptor’s oestrogen, progesterone and androgen. |
| Hexylresorcinol | Anti-ageing effects (anti-inflammatory and antioxidant) (Sivamani, 2015) reported in the literature. Anti-inflammatory effects detected by IPP (COX-1 and COX-2 inhibition). |
| Sulforaphane | Sulforaphane is a soft electrophile which induces a strong Nrf-2 upregulation and its downstream cytoprotective genes (haemoxygenase-1 and NAD(P)H:Quinone Oxireductase, glutathione-S-transferases) which increases the cellular defences against oxidative stress (Dinkova-Kostova et al., 2017). The observed bioactivity in the CSP, e.g. increase of GSH content and Nrf-2, are consistent with the literature. |
| Rosiglitazone | Rosiglitazone’s therapeutic effect as an antidiabetic drug was identified by the IPP data showing the PPAR-Y agonism at IC50 previously reported (Chen et al., 2017; Lecka-Czernik et al., 2007). Glitazones have been shown to be weak inhibitors of monoamino oxidases (Binda et al., 2012) and carbonic anhydrases (Mueller et al., 2021) also detected in the IPP data. Rosiglitazone causes mitochondrial toxicity through decreasing oxygen consumption (Hu et al., 2015), observed in the CPS at very low concentrations. |
| Doxorubicin | Doxorubicin proposed mechanisms: intercalation into DNA and disruption of topoisomerase-II-mediated DNA repair and generation of free radicals and their damage to cellular membranes, DNA and proteins (Thorn et al., 2011). Cell stress panel indicates a strong cytotoxic response with a global PoD of 1 nM. |

## T8. Impact of choosing different bioactivity platforms on toolbox performance

Protectiveness and utility of the toolbox for different platform POD subsets (IPP – *in vitro* pharmacological profiling, HTTr- High Throughput Transcriptomics, CSP – Cell Stress Panel). Values given in bold indicate platform POD subsets that give full protectiveness at all three PBK levels.

| **Platform POD**  **subset** | **Cell type(s)** | **Utility L1** | **Protectiveness L1** | **Utility L2** | **Protectiveness L2** | **Utility L3** | **Protectiveness L3** |
| --- | --- | --- | --- | --- | --- | --- | --- |
| IPP | - | 2/18 (11%) | 6/6 (100%) | 6/18 (33%) | 6/6 (100%) | 6/13 (46%) | 4/5 (80%) |
| CSP | HepG2 | **7/18 (39%)** | **6/6 (100%)** | **12/18 (67%)** | **6/6 (100%)** | **12/13 (92%)** | **5/5 (100%)** |
| HTTr BIFROST | MCF-7 | **4/18 (22%)** | **6/6 (100%)** | **6/18 (33%)** | **6/6 (100%)** | **9/13 (69%)** | **5/5 (100%)** |
|  | HepG2 | **5/18 (28%)** | **6/6 (100%)** | **10/18 (56%)** | **6/6 (100%)** | **10/13 (77%)** | **6/6 (100%)** |
|  | HepaRG | **7/18 (39%)** | **6/6 (100%)** | **12/18 (67%)** | **6/6 (100%)** | **12/13 (92%)** | **5/5 (100%)** |
|  | MCF-7, HepG2 | **4/18 (22%)** | **6/6 (100%)** | **6/18 (33%)** | **6/6 (100%)** | **9/13 (69%)** | **5/5 (100%)** |
|  | MCF-7, HepaRG | **4/18 (22%)** | **6/6 (100%)** | **6/18 (33%)** | **6/6 (100%)** | **9/13 (69%)** | **5/5 (100%)** |
|  | HepG2, HepaRG | **5/18 (28%)** | **6/6 (100%)** | **10/18 (56%)** | **6/6 (100%)** | **10/13 (77%)** | **5/5 (100%)** |
|  | MCF-7, HepG2, HepaRG | **4/18 (22%)** | **6/6 (100%)** | **6/18 (33%)** | **6/6 (100%)** | **9/13 (69%)** | **5/5 (100%)** |
| HTTr BMD | MCF-7 | 11/18 (61%) | 5/6 (83%) | 15/18 (83%) | 4/6 (67%) | 13/13 (100%) | 3/5 (60%) |
|  | HepG2 | 12/18 (67%) | 5/6 (83%) | 14/18 (78%) | 4/6 (67%) | 13/13 (100%) | 2/5 (40%) |
|  | HepaRG | 13/18 (72%) | 5/6 (83%) | 18/18 (100%) | 4/6 (67%) | 13/13 (100%) | 2/5 (40%) |
|  | MCF-7, HepG2 | 11/18 (61%) | 5/6 (83%) | 14/18 (78%) | 4/6 (67%) | 13/13 (100%) | 3/5 (60%) |
|  | MCF-7, HepaRG | 10/18 (56%) | 5/6 (83%) | 15/18 (83%) | 4/6 (67%) | 13/13 (100%) | 3/5 (60%) |
|  | HepG2, HepaRG | 11/18 (61%) | 5/6 (83%) | 14/18 (78%) | 4/6 (67%) | 13/13 (100%) | 2/5 (40%) |
|  | MCF-7, HepG2, HepaRG | 10/18 (56%) | 5/6 (83%) | 14/18 (78%) | 4/6 (67%) | 13/13 (100%) | 3/5 (60%) |

# Supplementary Figures

## F1. Cmax Error Distribution model cross validation

95% prediction interval estimates of the measured C_max_ for the 11 exposure scenarios where this measurement was available (vertical line). Estimates were obtained using a cross-validation procedure (see Materials and Methods). PBK C_max_ estimates for a given PBK level are shown as red crosses.


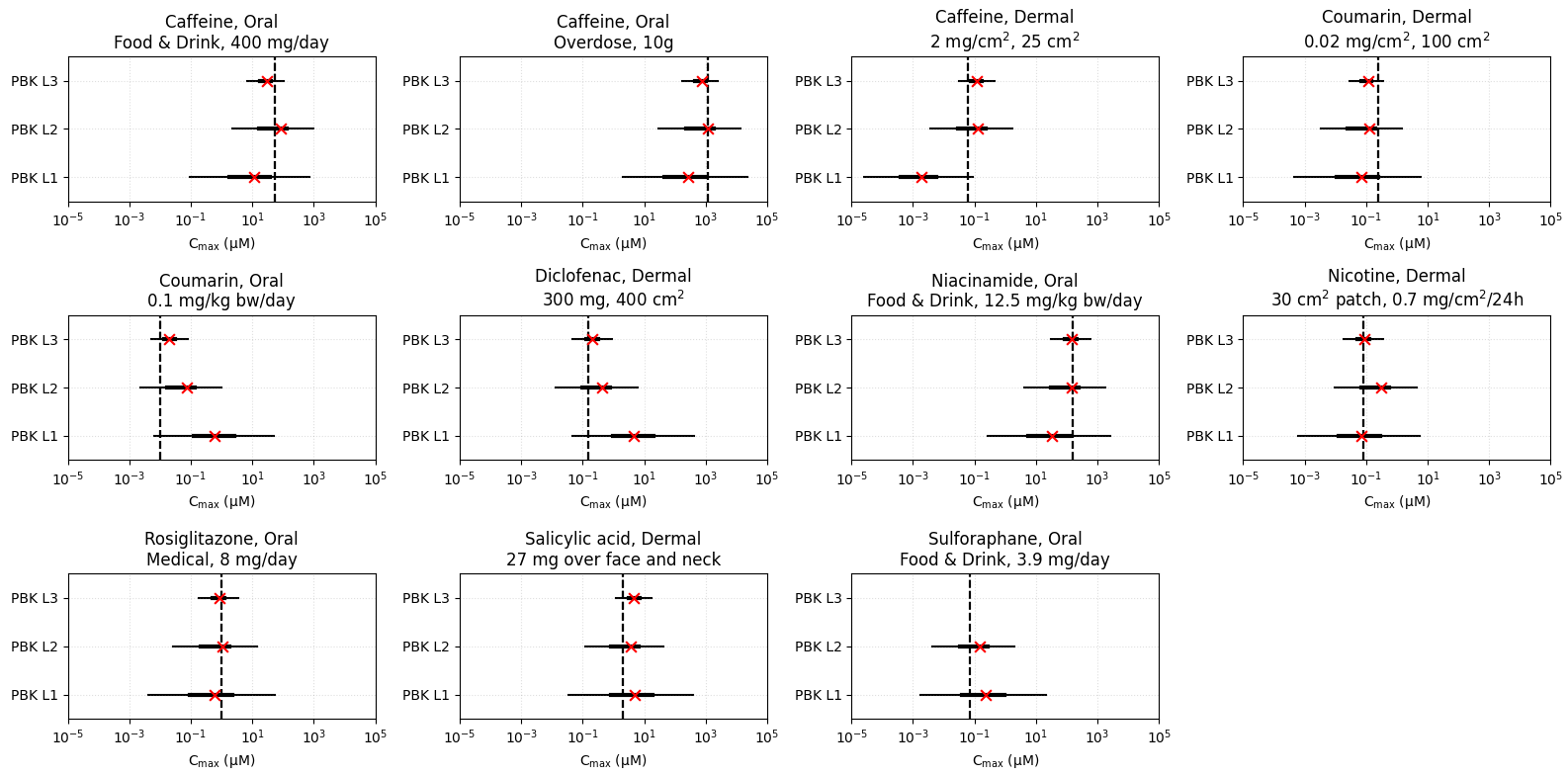


## F2. Dose confirmation data

Summary of the dose confirmation experiments for butylated hydroxytoluene (BHT), coumarin, doxorubicin, oxybenzone, paraquat dichloride, sulforaphane and valproic acid. The top row shows the nominal concentration (red squares) compared to the average measured values for the two test plates (green and black circles). The bottom row shows the corresponding measured to nominal concentration ratios, with horizontal dashed lines indicating different fold differences between the values. Valproic acid was the only test chemical with differences in excess of 2-fold across all tested concentrations, indicating potential systematic issues with the testing of this particular chemical in the *in vitro* assays.


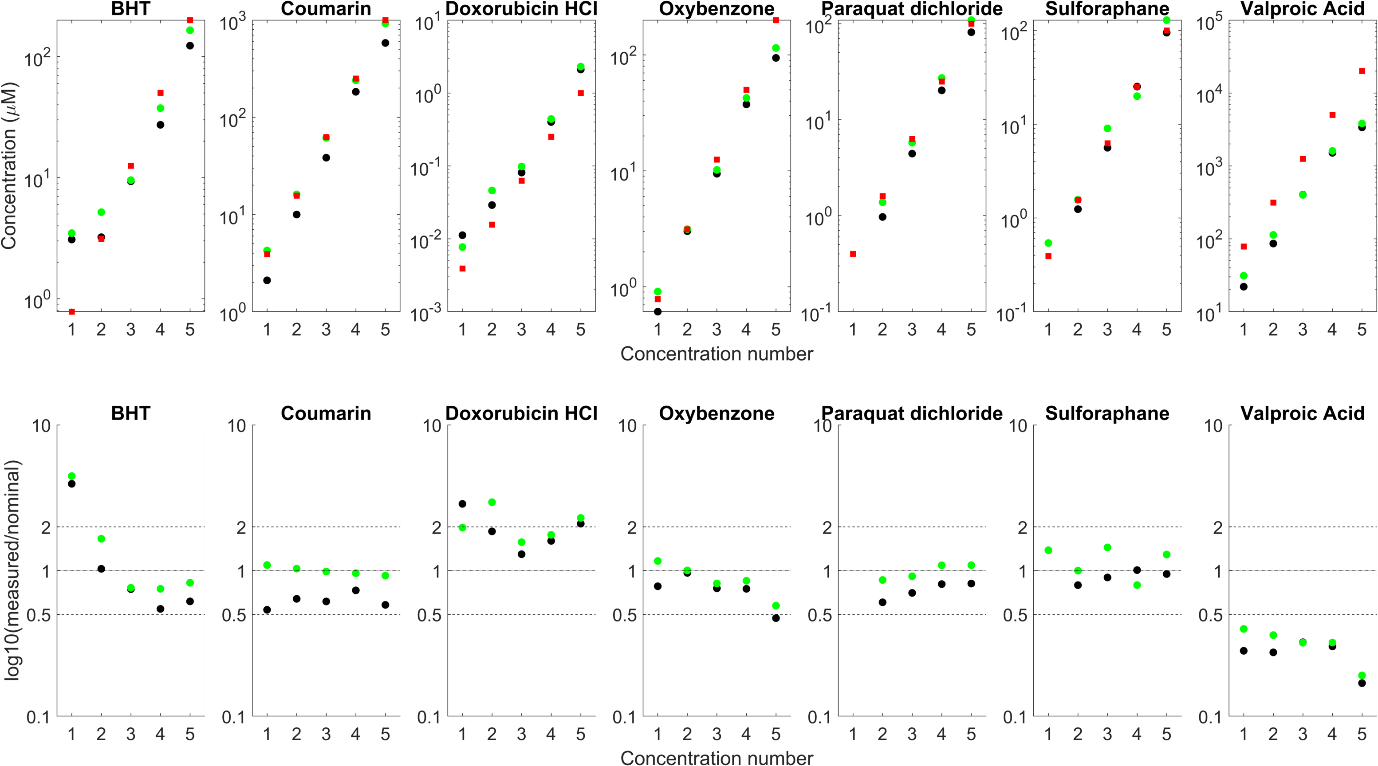


## F3. Cell stress

Summary plots of cell stress panel bioactivity per chemical. Blue densities indicate PoDs for assay-specific biomarkers; orange densities indicate PoDs for assay-specific cell health markers (Cell Count, Nuclear Area, DNA Structure). Corresponding cell health biomarkers are not measured within the Extracellular Flux (Seahorse) assay (biomarkers: ECAR, OCR and Reserve Capacity). Multiple peaks in the density indicate bimodality in the pooled distribution of the data for a single biological replicate or because biological replicates differ in terms of sensitivity. For cell health distributions (orange), bimodality may also occur due to difference in the sensitivity of each cell health biomarker. The vertical black line indicates the global PoD across all measured biomarkers.

### Caffeine


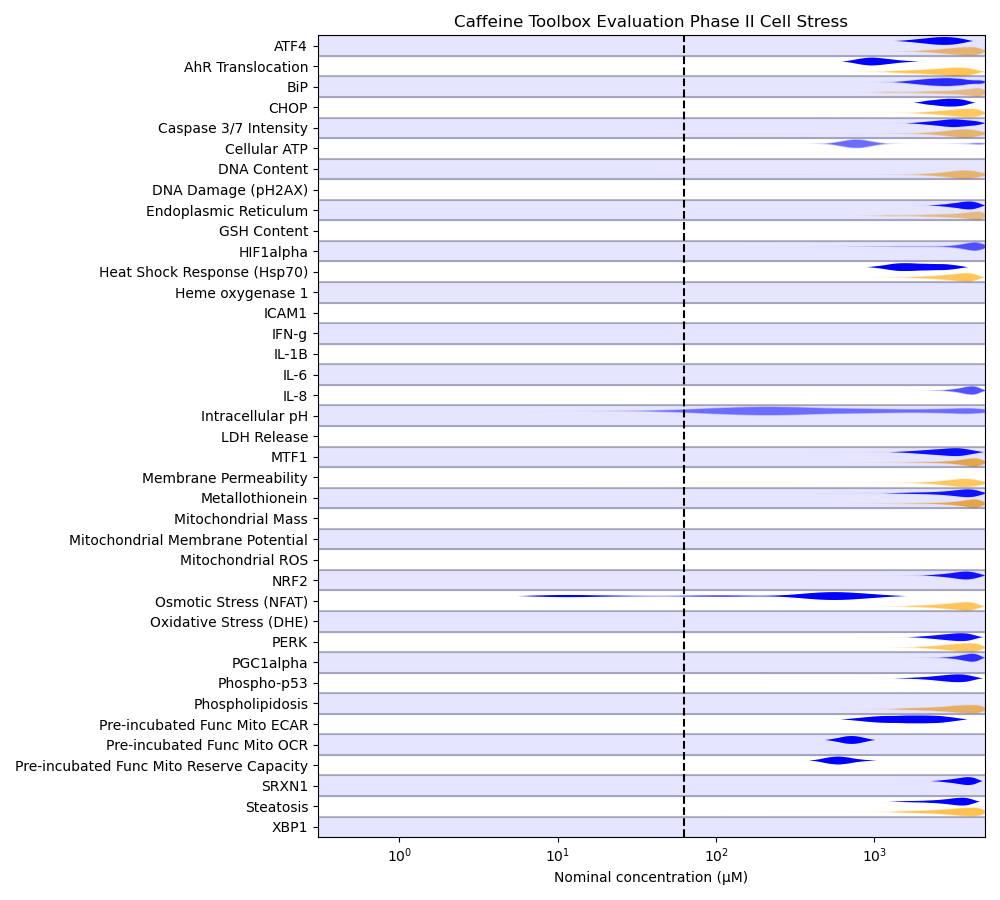


### Coumarin


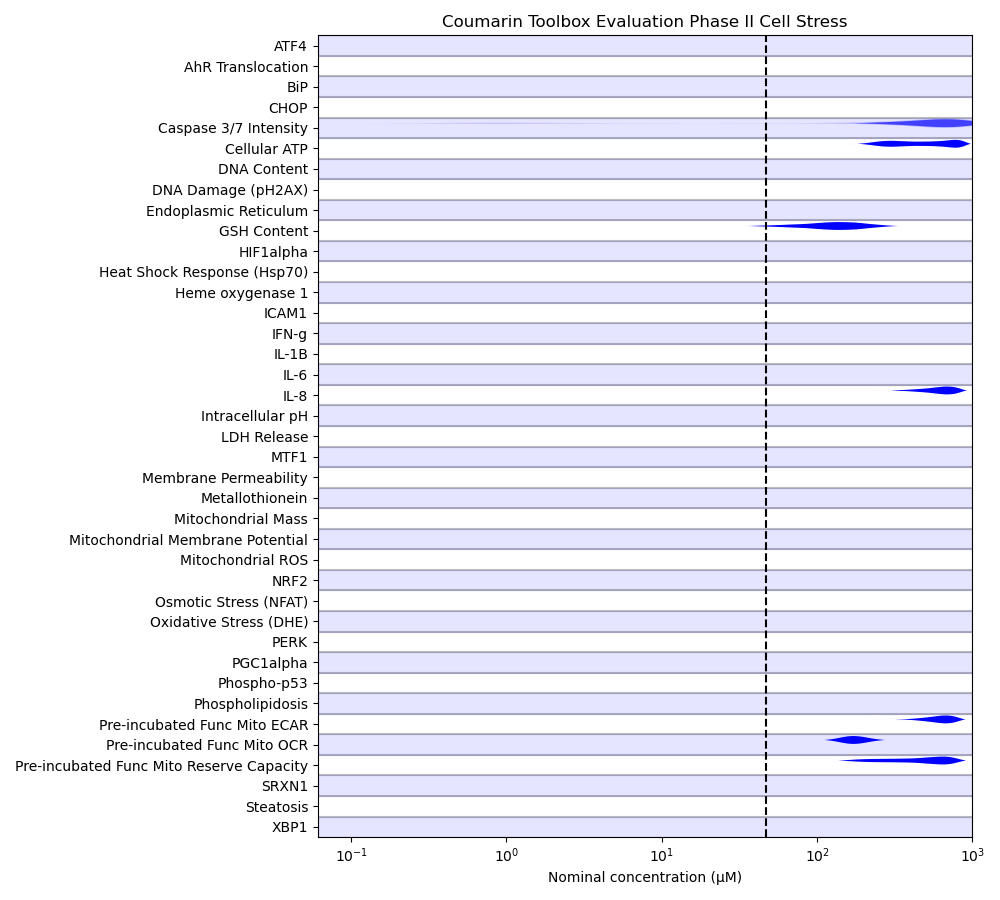


### Paraquat dichloride


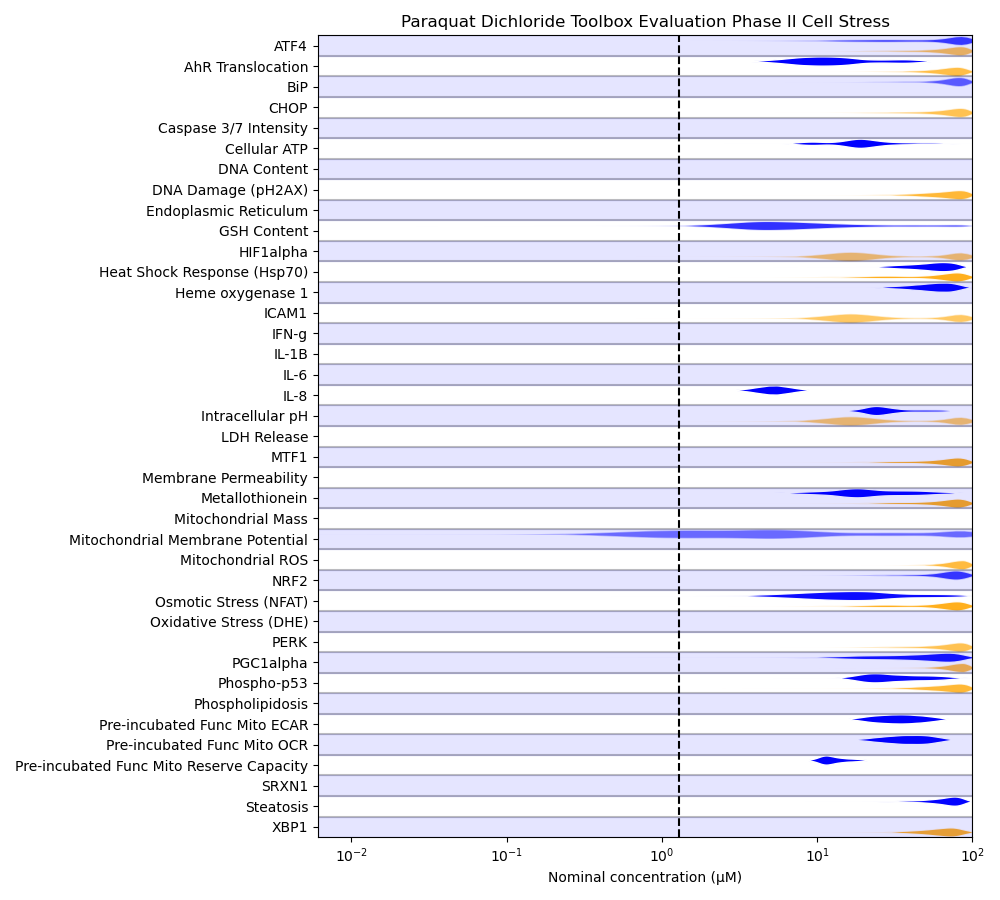


### Butylated Hydroxytoluene (BHT)


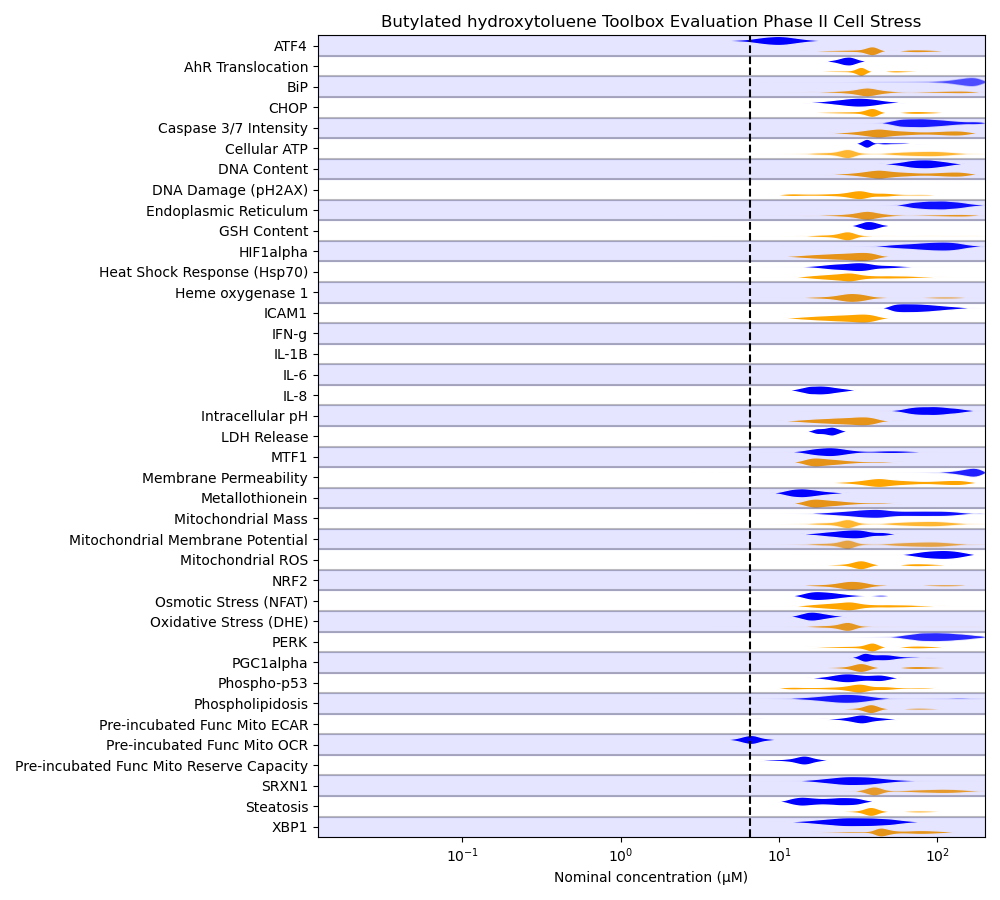


### Oxybenzone


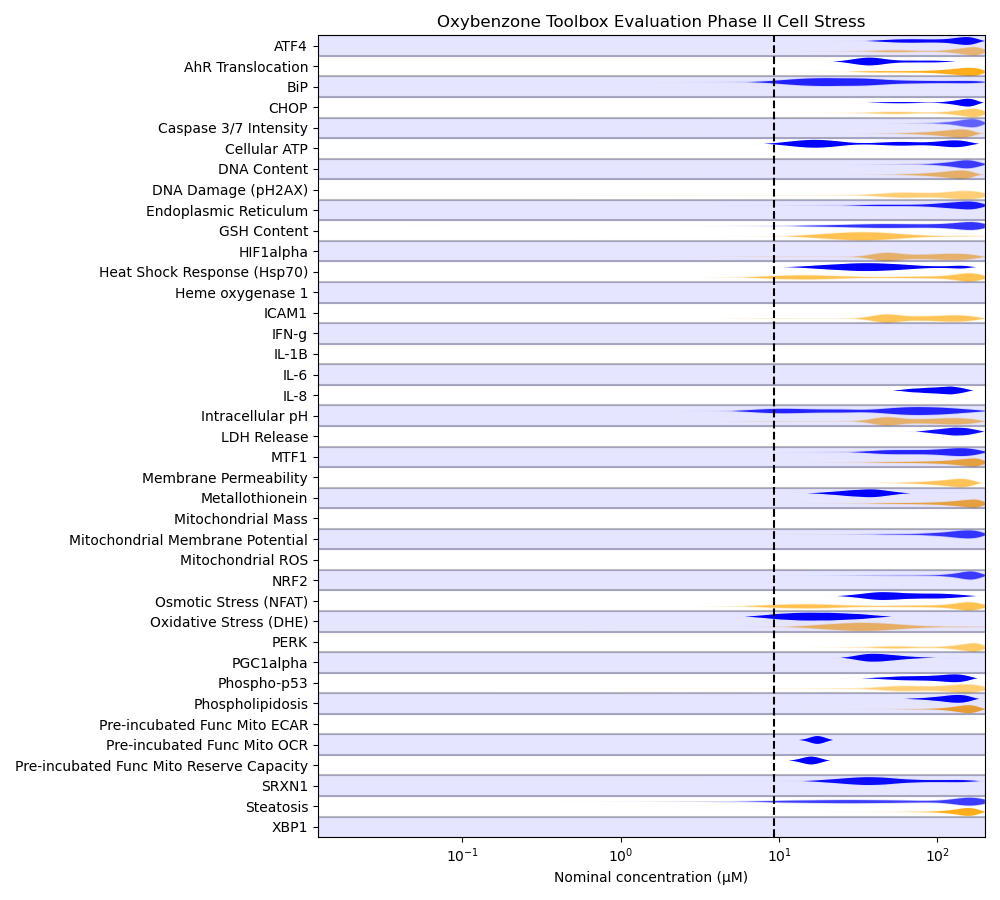


### 4-Hexylresorcinol


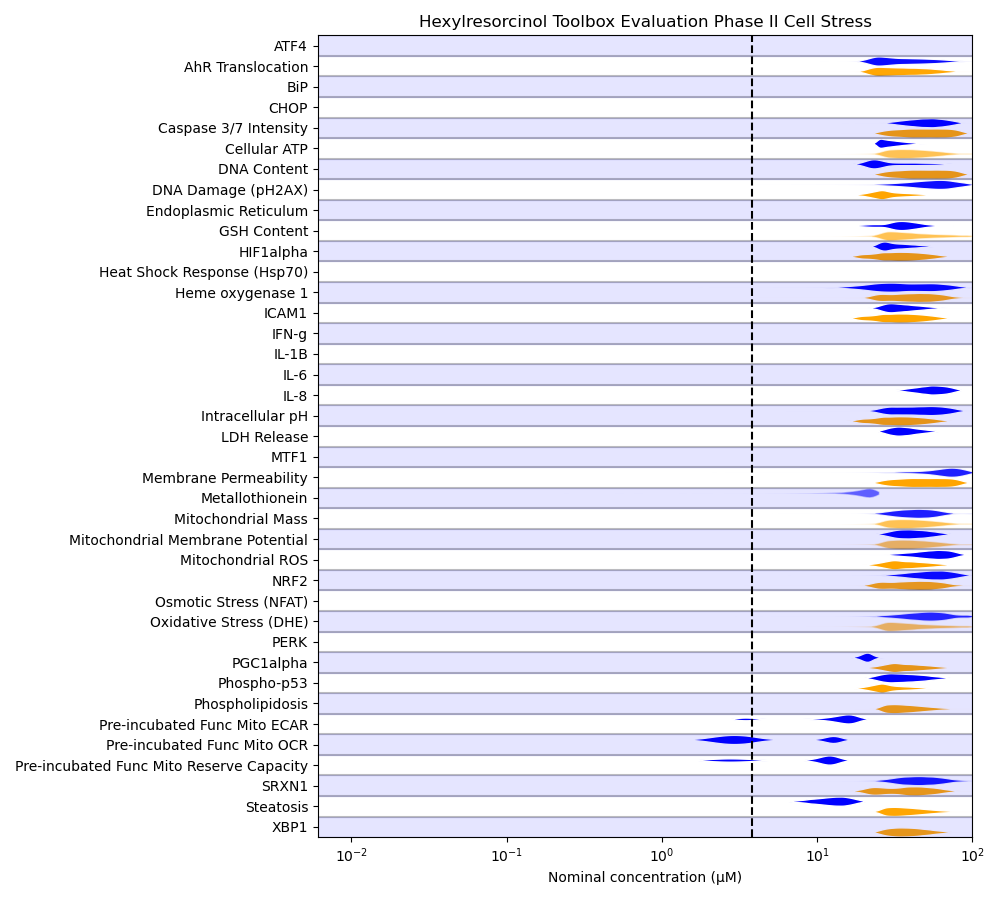


### Sulforaphane


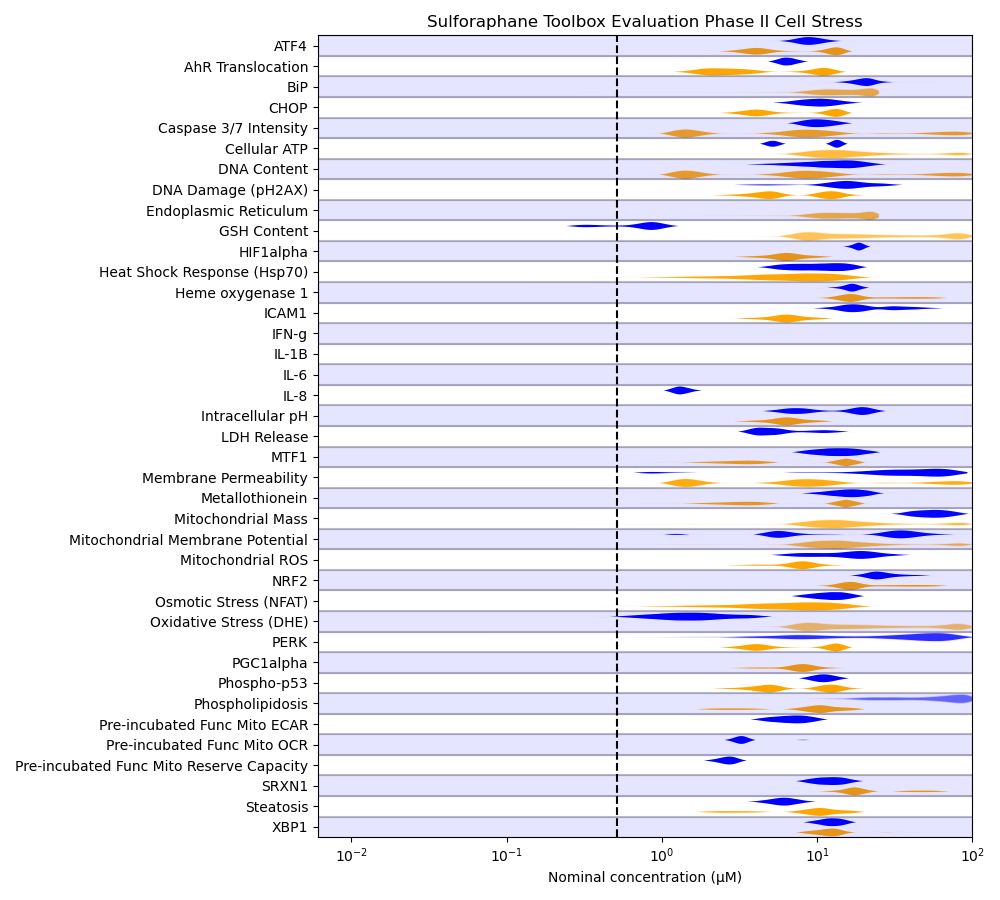


### Rosiglitazone


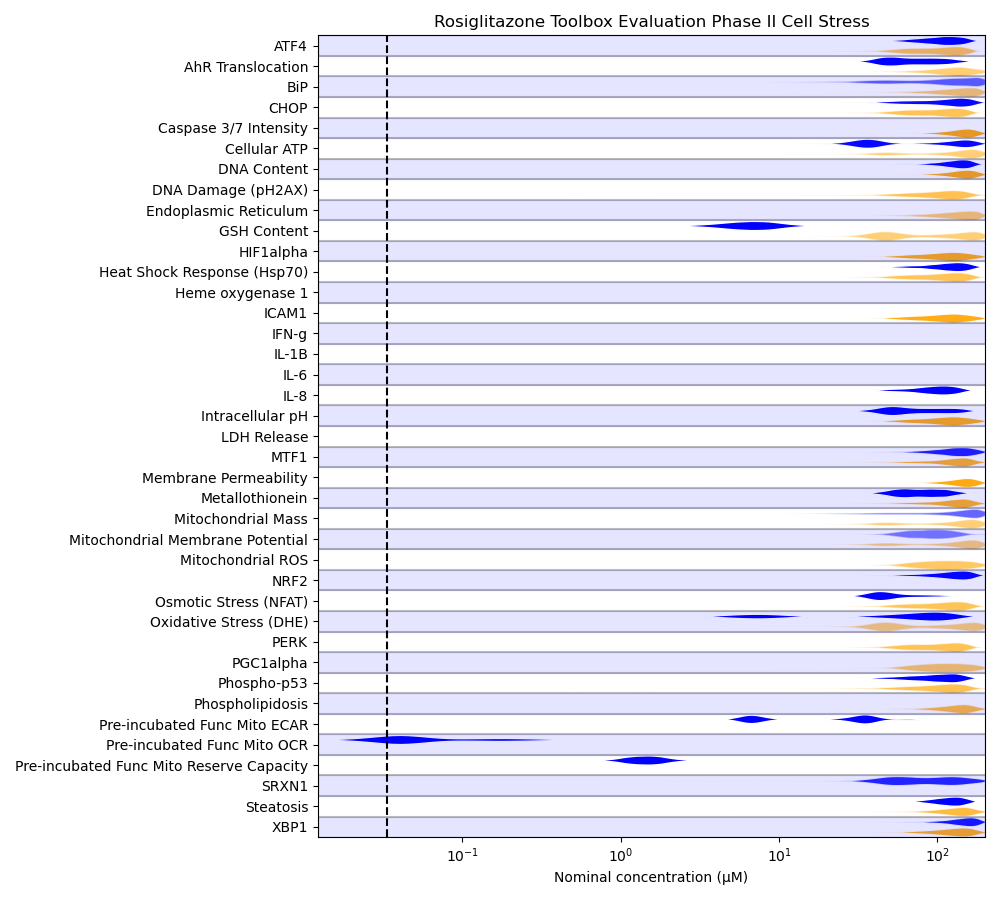


### Niacinamide


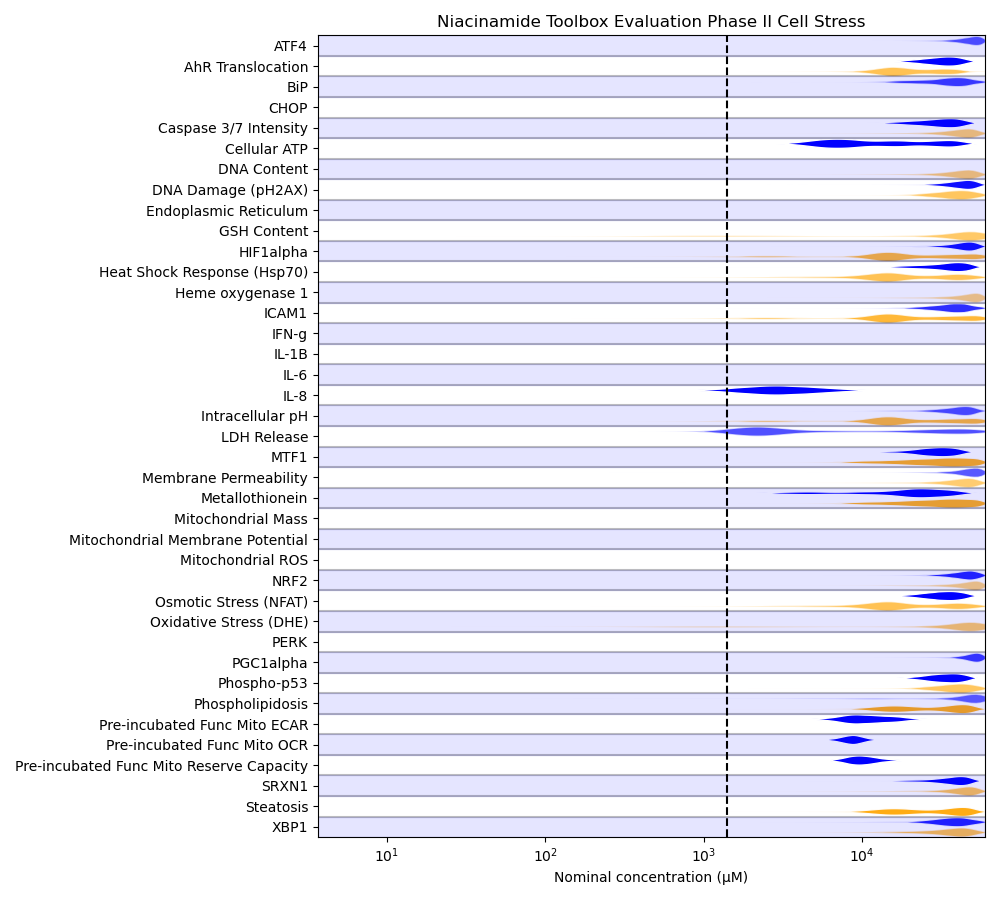


### Doxorubicin hydrochloride


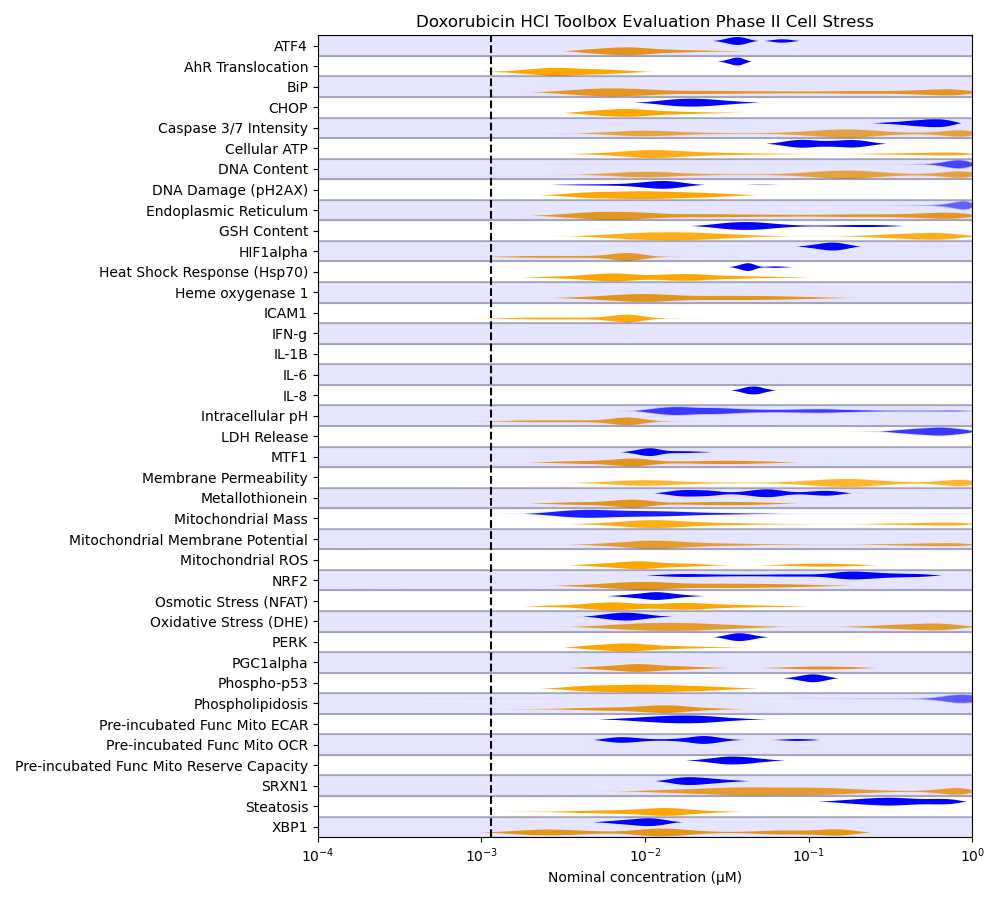


## F4. IPP

Dose response plots for the binding/functional assays that showed a response above 50% threshold following a screening at either 10 uM or 100 uM. Concentration of the chemical is shown on X-axis whereas displacement of the radiolabelled ligand is shown on Y-axis. Fitting of the response curves has been done as per Materials and methods. The dash lines represent 95% C.I., the yellow points are the measured data, and the solid line is the estimated median response.

### Caffeine

Caffeine-ADORA2A, 95% C.I.(IC50) = [5.3µM, 7.6µM]


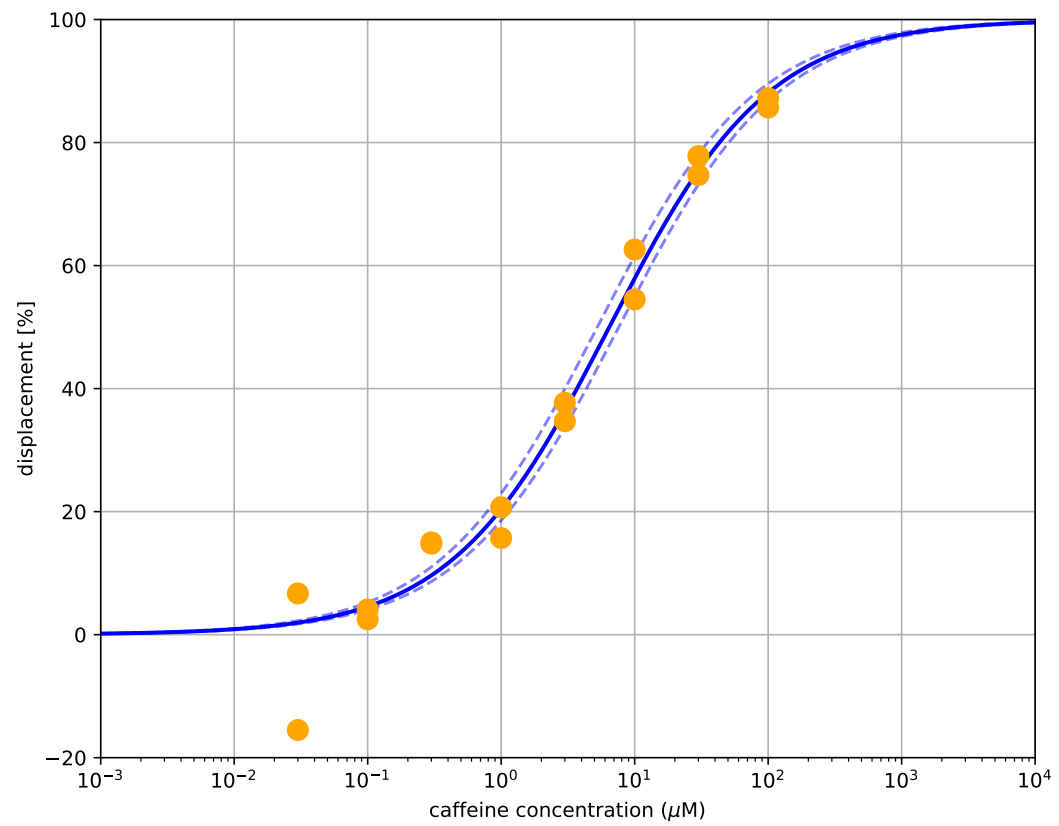


### Coumarin

Coumarin-MAOA, 95% C.I.(IC50) = [26.2µM, 36.5µM]


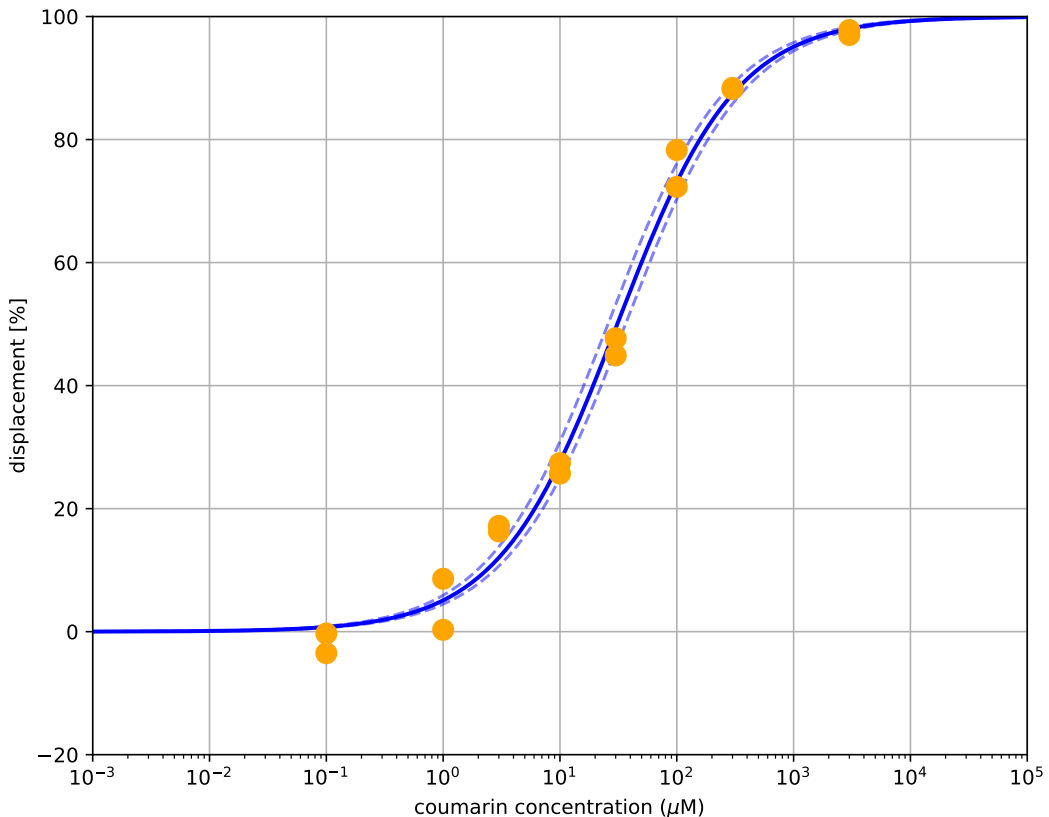


### Doxorubicin Hydrochloride

Doxorubicin-PTGS2, 95% C.I.(IC50) = [0.8µM, 1.7µM]


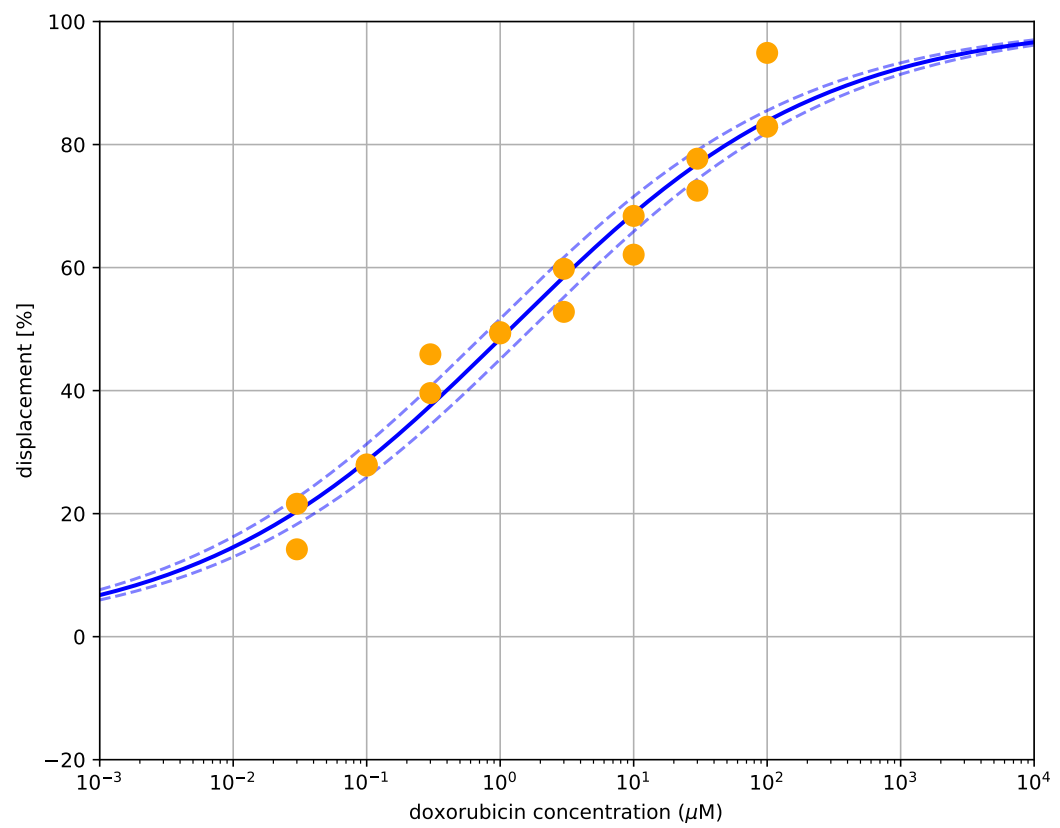


Doxorubicin-LCK, 95% C.I.(IC50) = [9.2µM, 37.2µM]


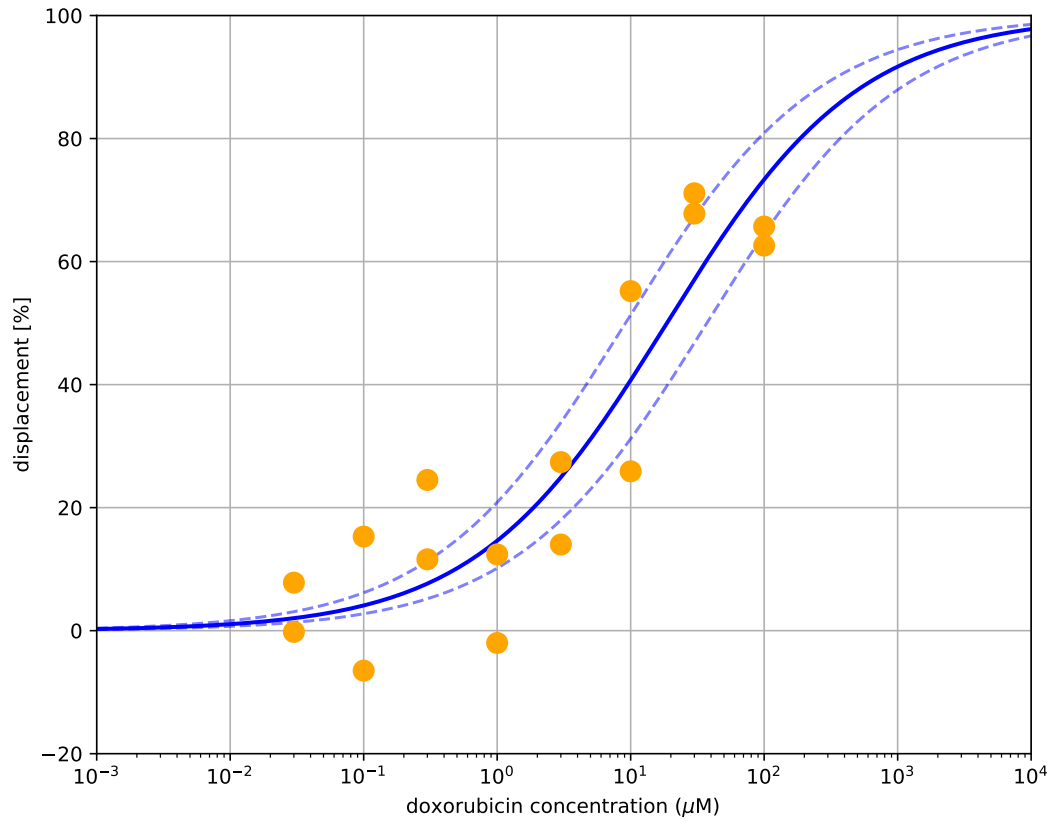


### 4-Hexylresorcinol

4-Hexylresorcinol-PTGS1, 95% C.I.(IC50) = [0.2µM, 0.4µM]


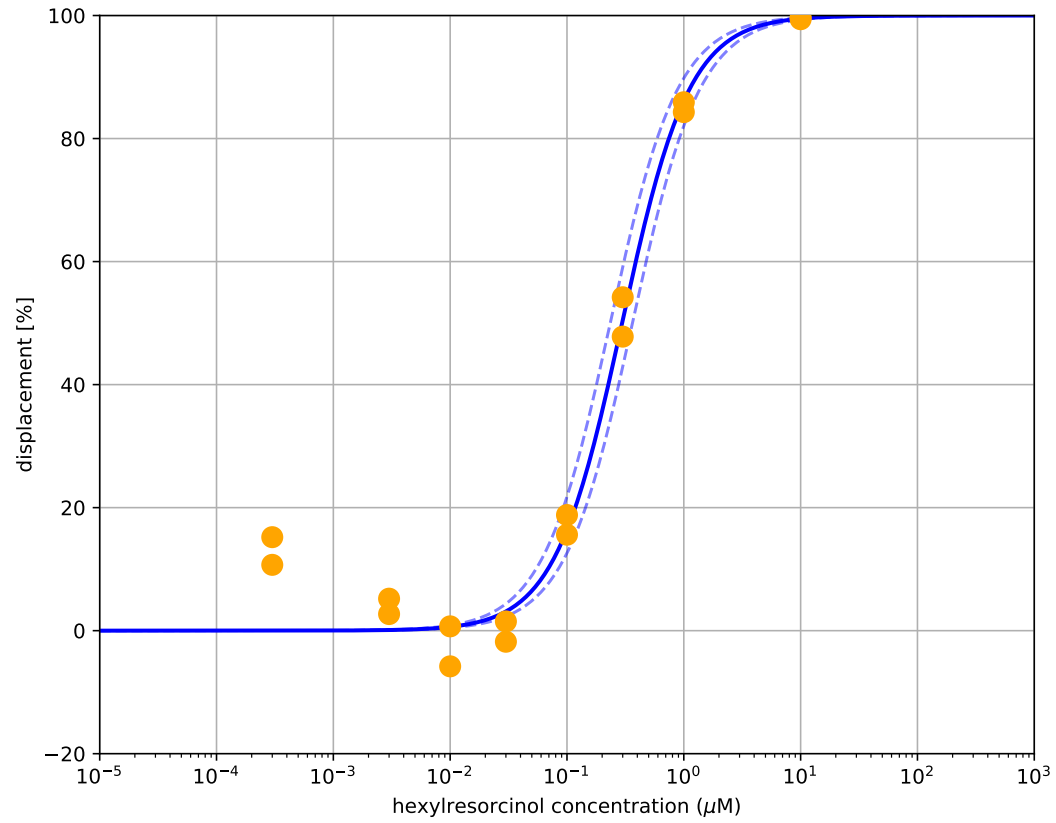


4-Hexylresorcinol-PTGS2, 95% C.I.(IC50) = [1.4µM, 2.1µM]


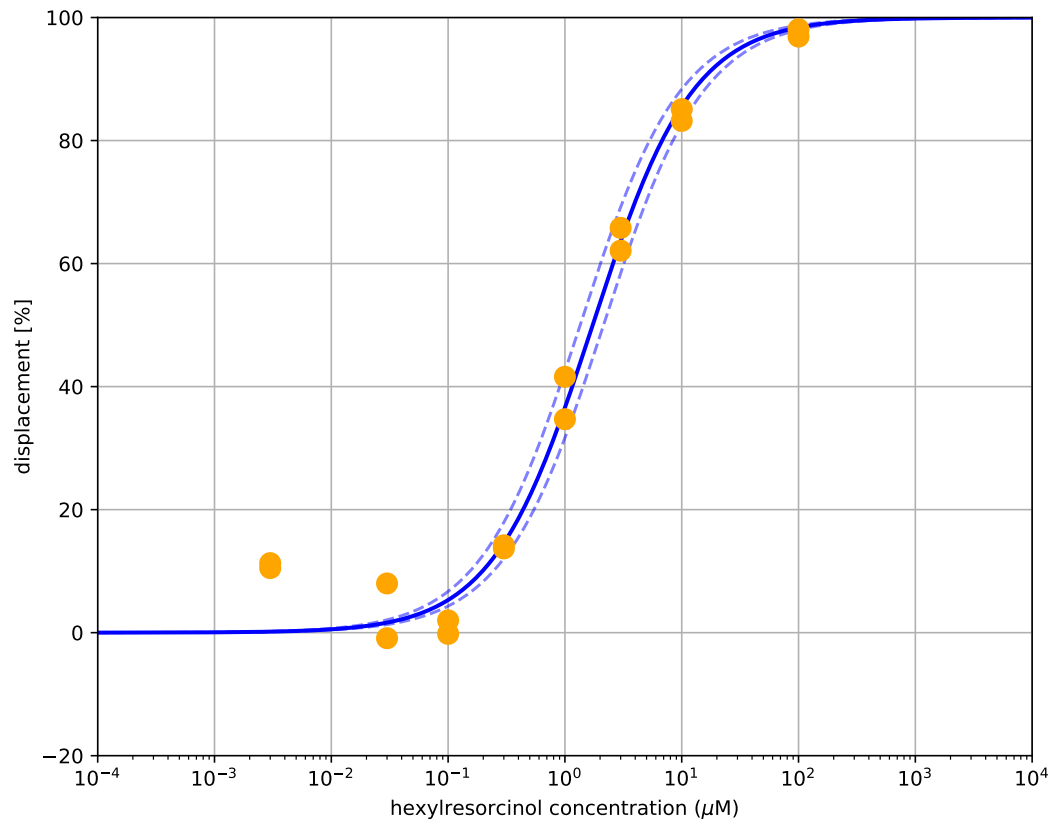


4-Hexylresorcinol-HTR2B, 95% C.I.(IC50) = [5.7µM, 9.6µM]


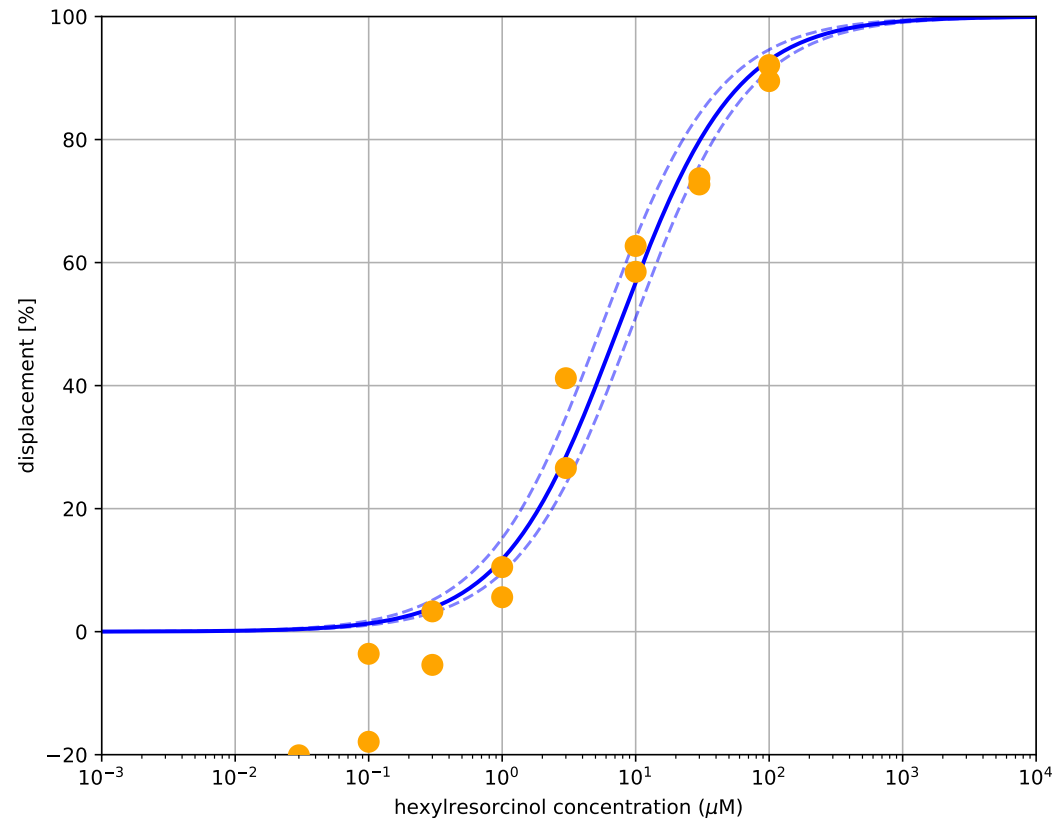


4-Hexylresorcinol-SLC6A2, 95% C.I.(IC50) = [7.3µM, 9.5µM]


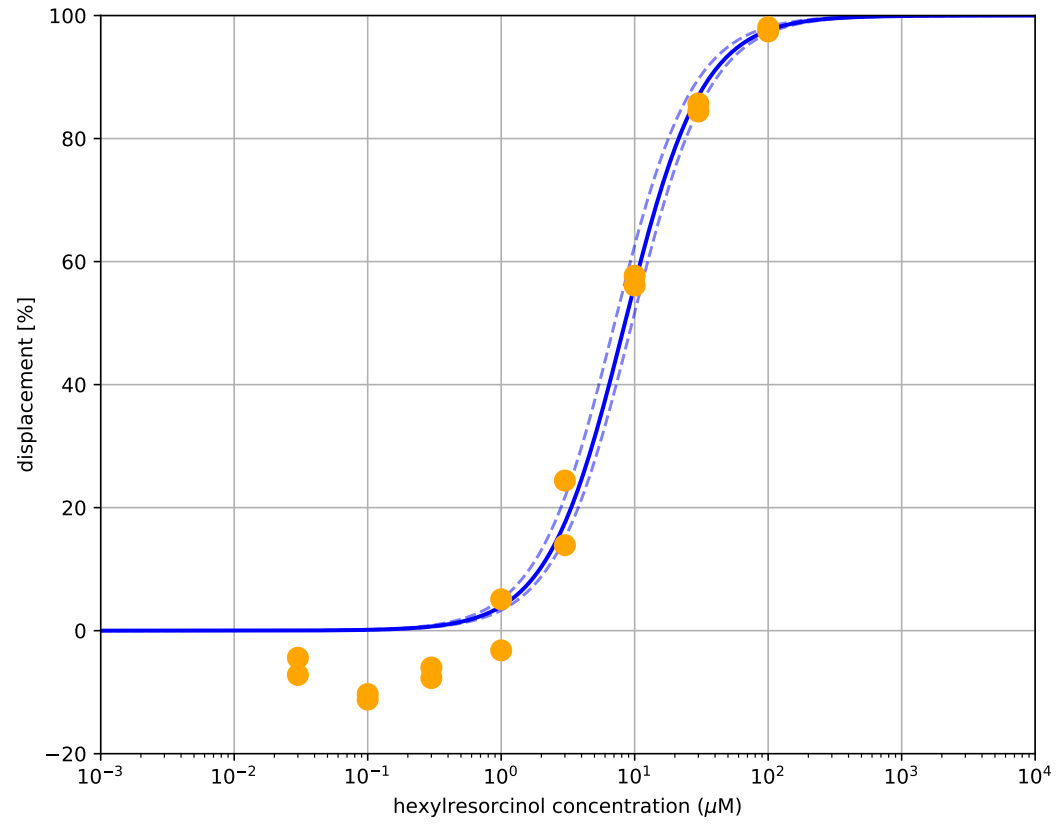


### Oxybenzone

Oxybenzone-MAOA, 95% C.I.(IC50) = [12.9µM, 21.8µM]


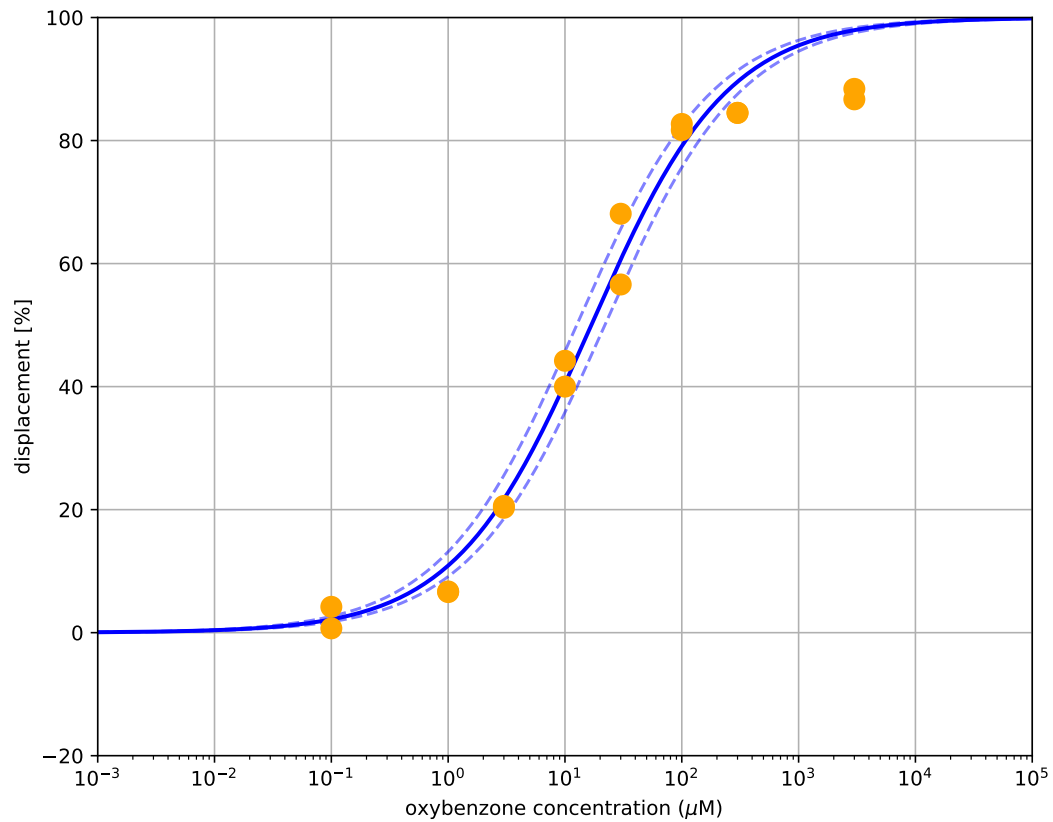


Oxybenzone-PDE4D, 95% C.I.(IC50) = [13.0µM, 27.4µM]


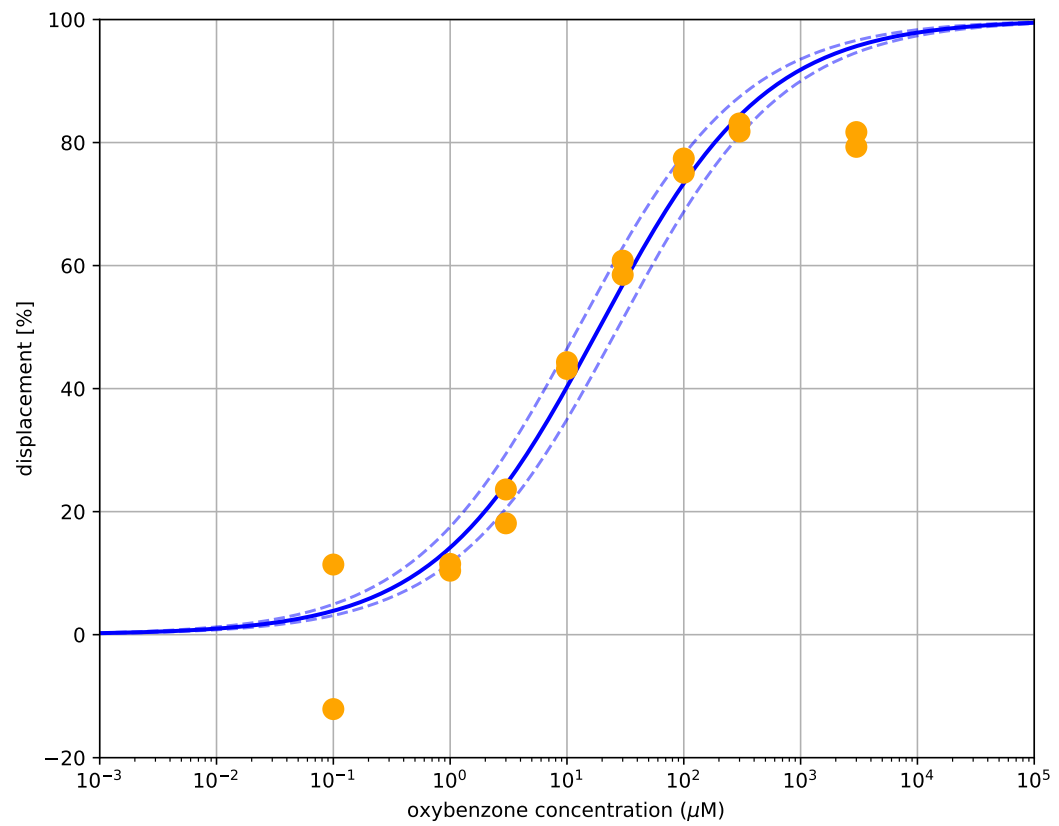


Oxybenzone-OPRK1, 95% C.I.(IC50) = [55.9µM, 130.5µM]


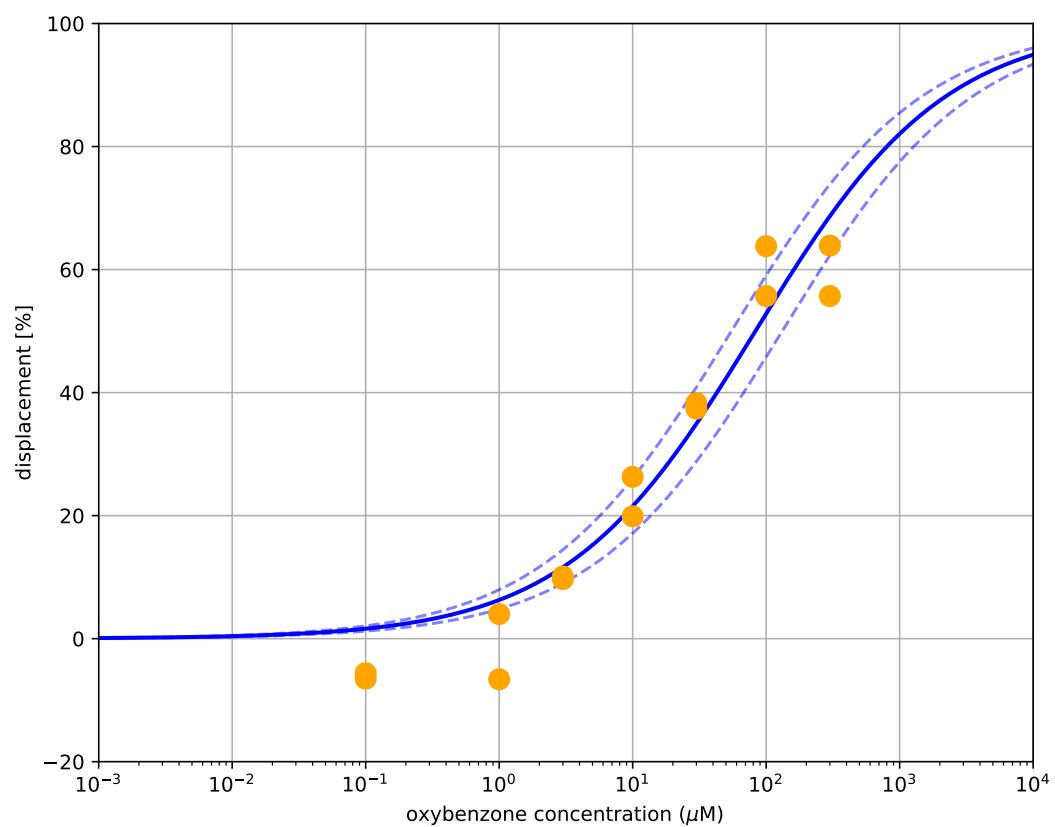


Oxybenzone-PTGS1, 95% C.I.(IC50) = [47.2µM, 117.8µM]


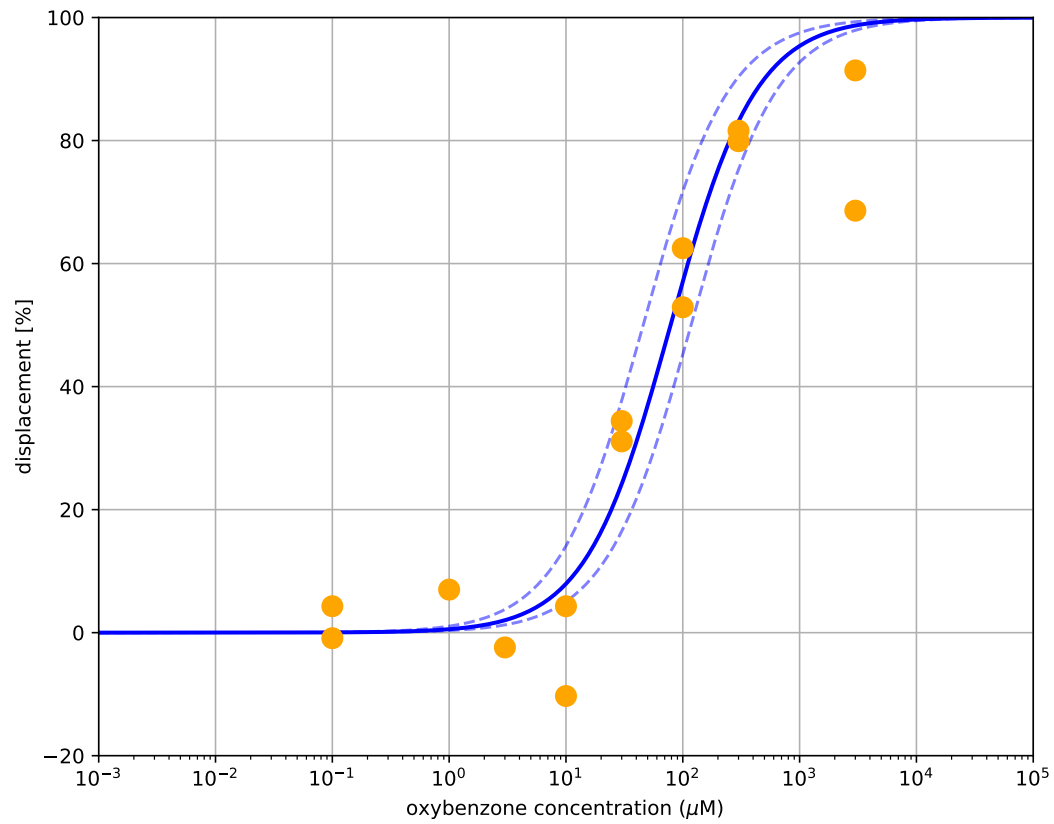


Oxybenzone-CACNA1C, 95% C.I.(IC50) = [48.1µM, 117.9µM]


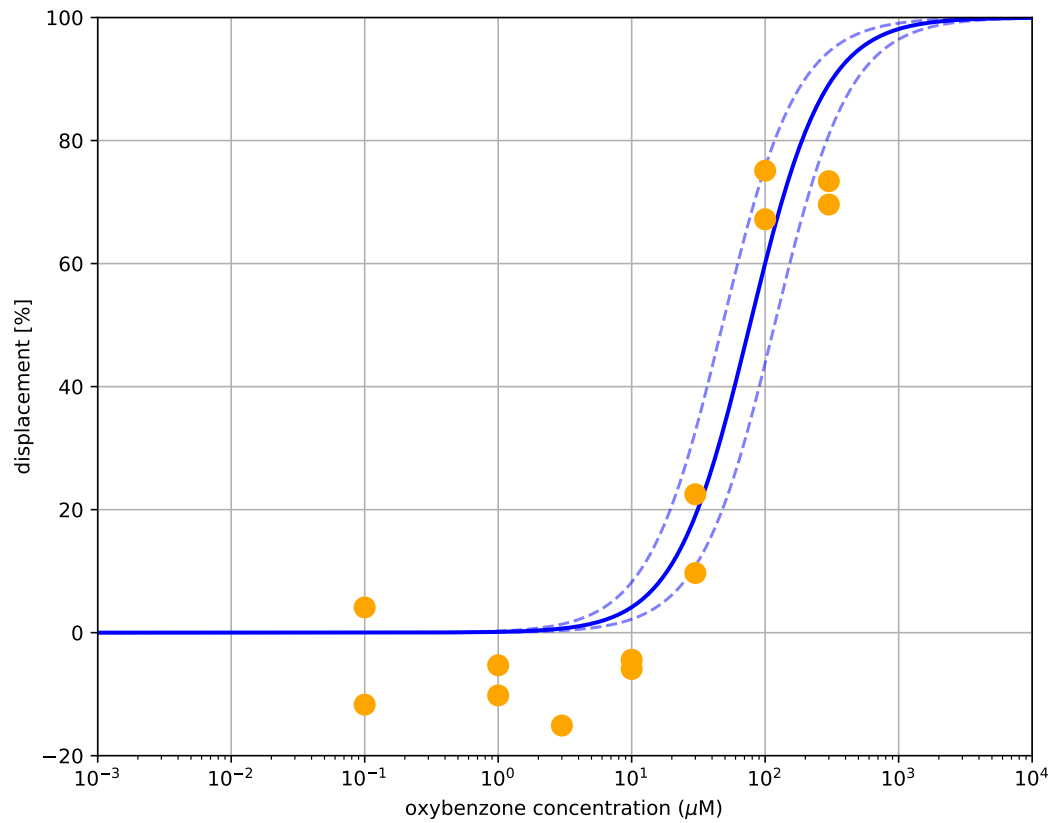


Oxybenzone-CCKAR, 95% C.I.(IC50) = [51.1µM, 85.1µM]


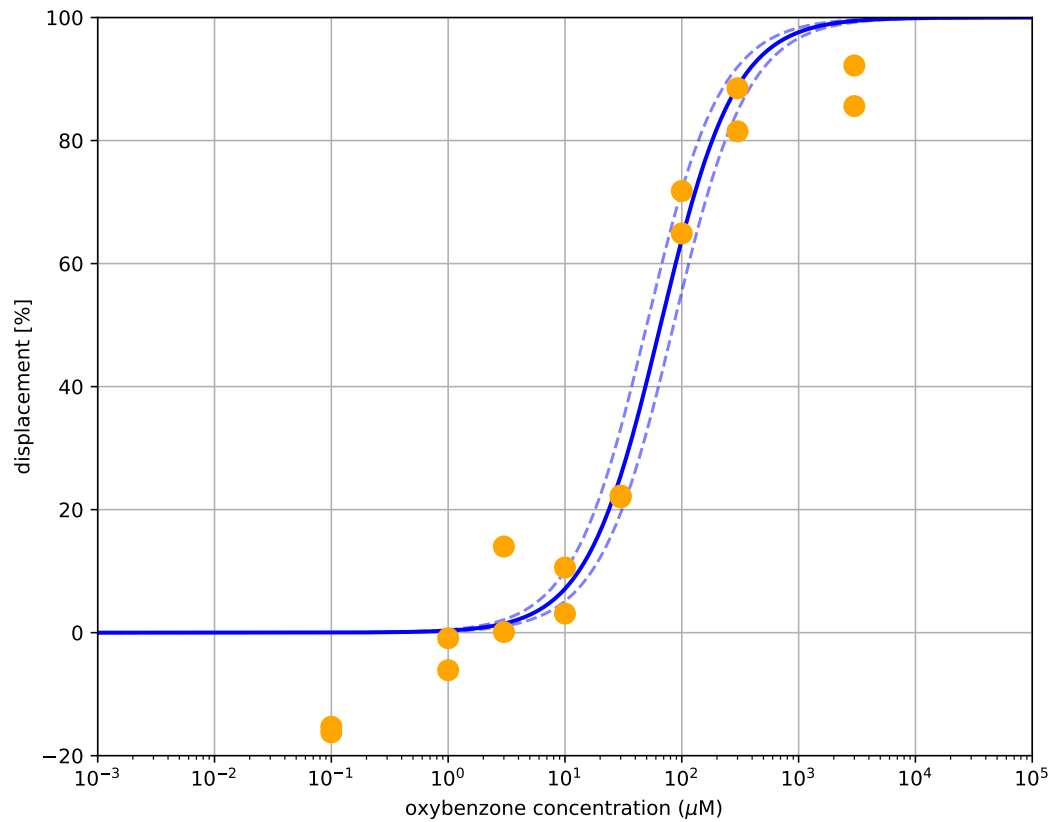


Oxybenzone-SLC6A3, 95% C.I.(IC50) = [28.0µM, 47.5µM]


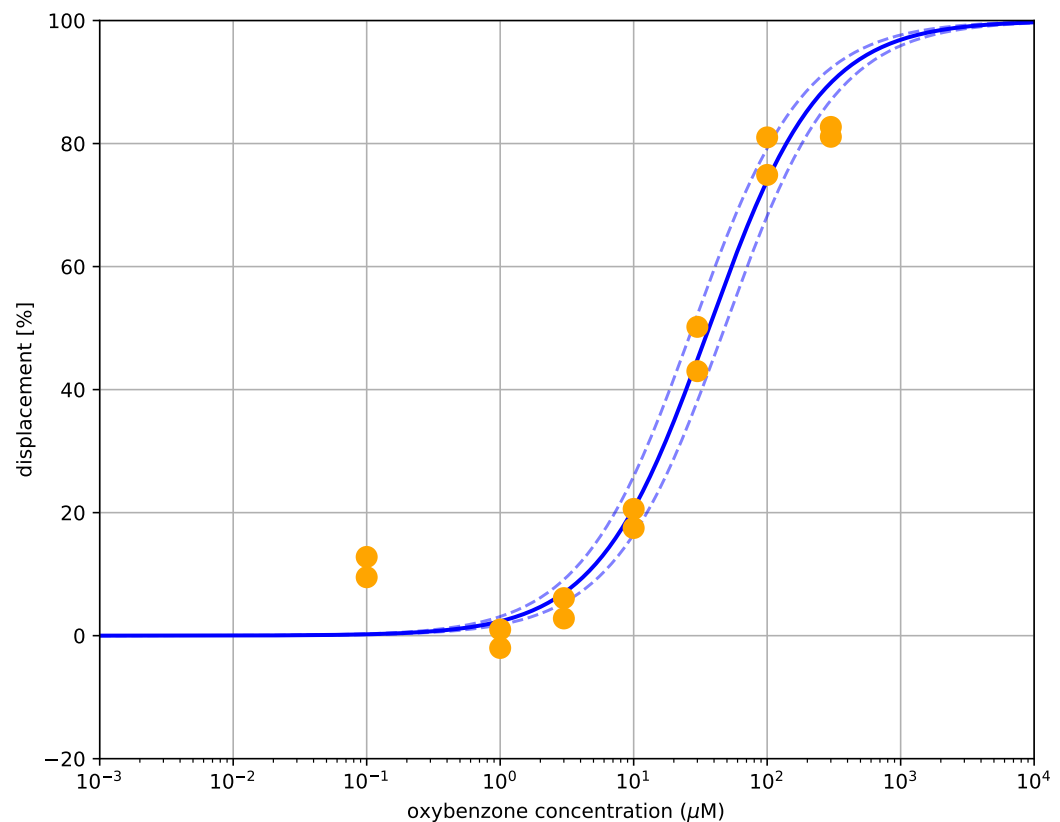


Oxybenzone-ADORA2A, 95% C.I.(IC50) = [29.8µM, 41.0µM]


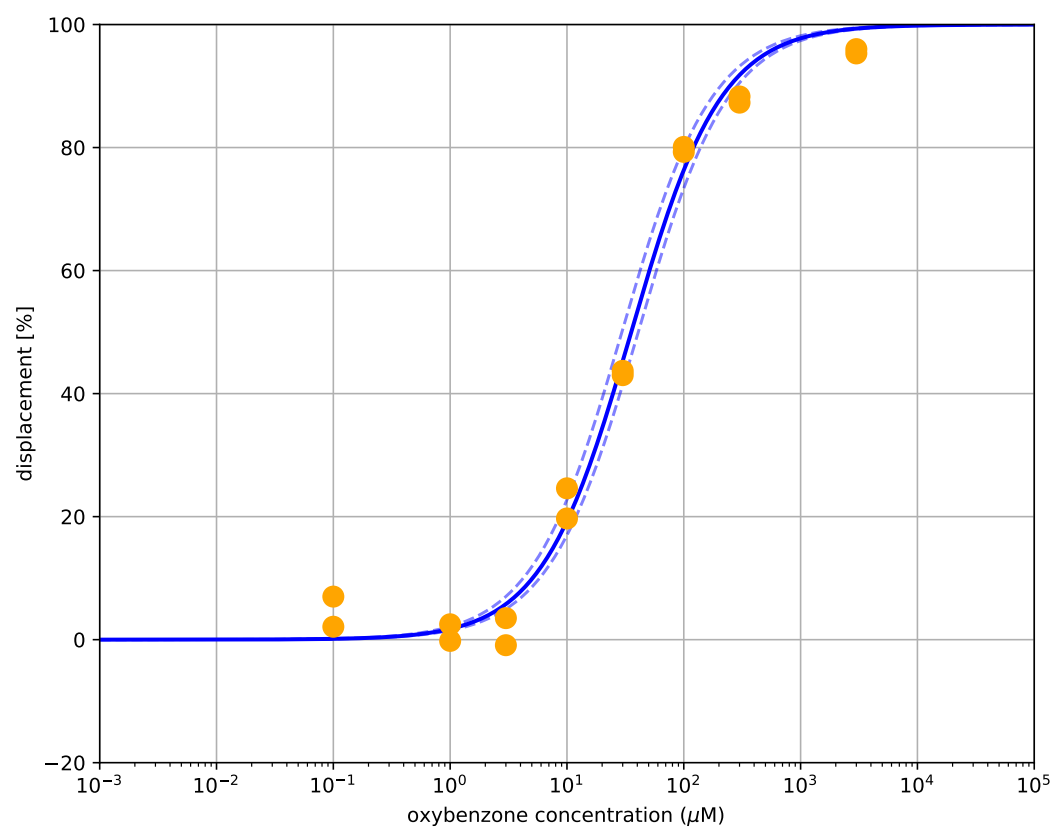


Oxybenzone-HTR2B, 95% C.I.(IC50) = [2.8µM, 4.3µM]


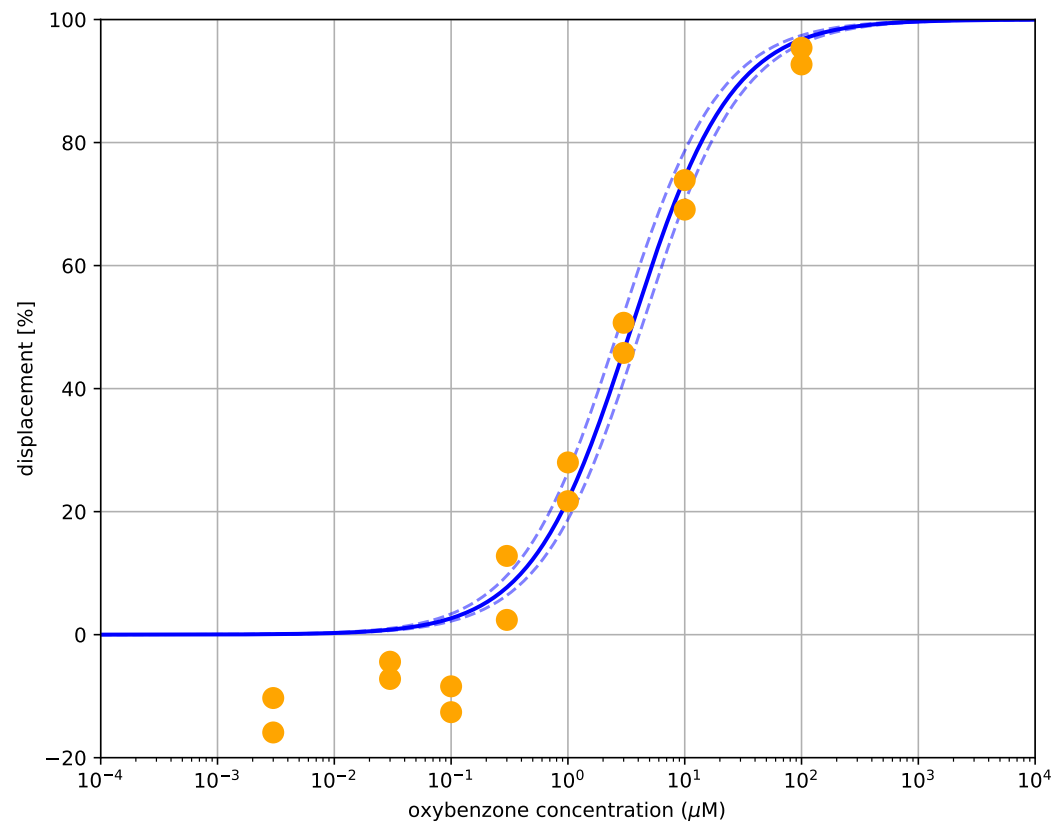


Oxybenzone-HTR2A, 95% C.I.(IC50) = [99.8µM, 152.4µM]


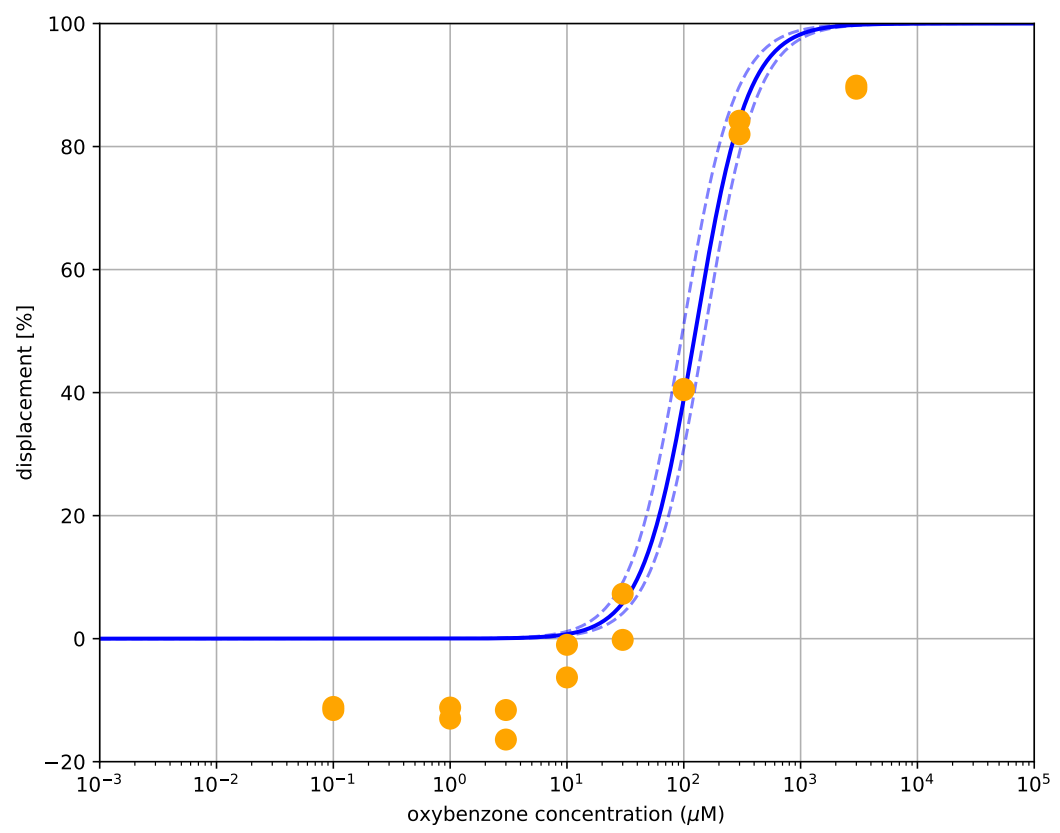


Oxybenzone-KCNH2, 95% C.I.(IC50) = [42.2µM, 101.5µM]


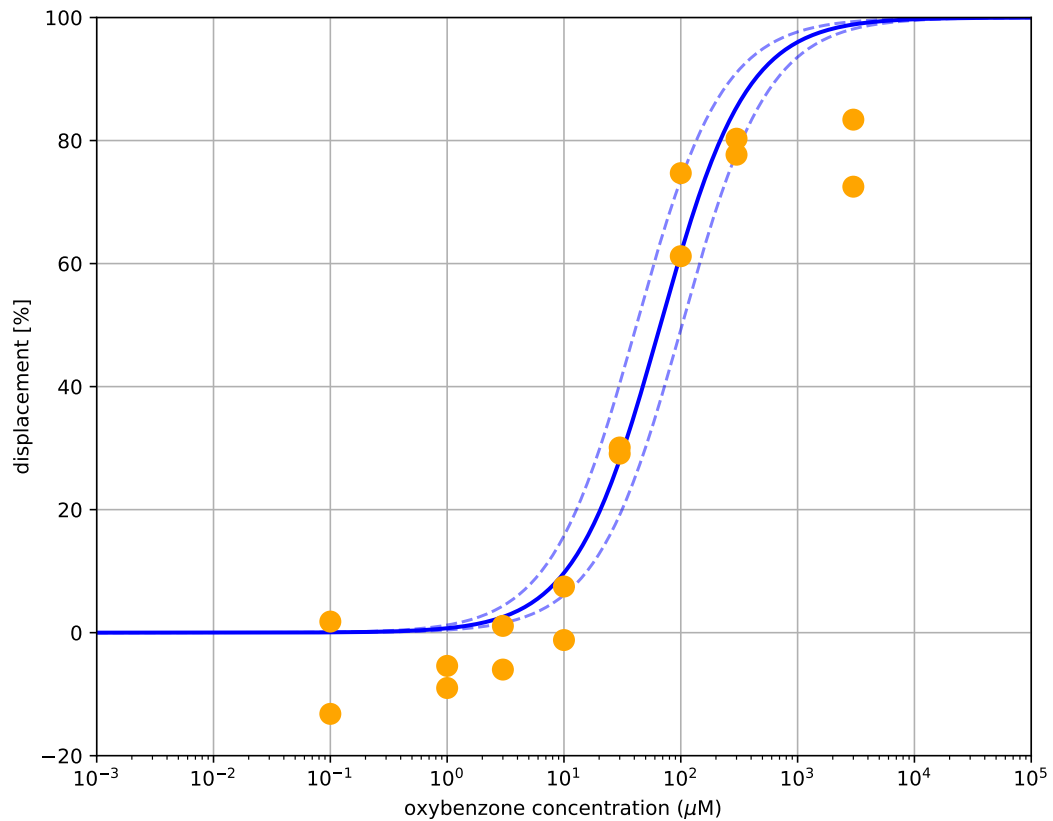


Oxybenzone-SLC6A2, 95% C.I.(IC50) = [35.6µM, 43.8µM]


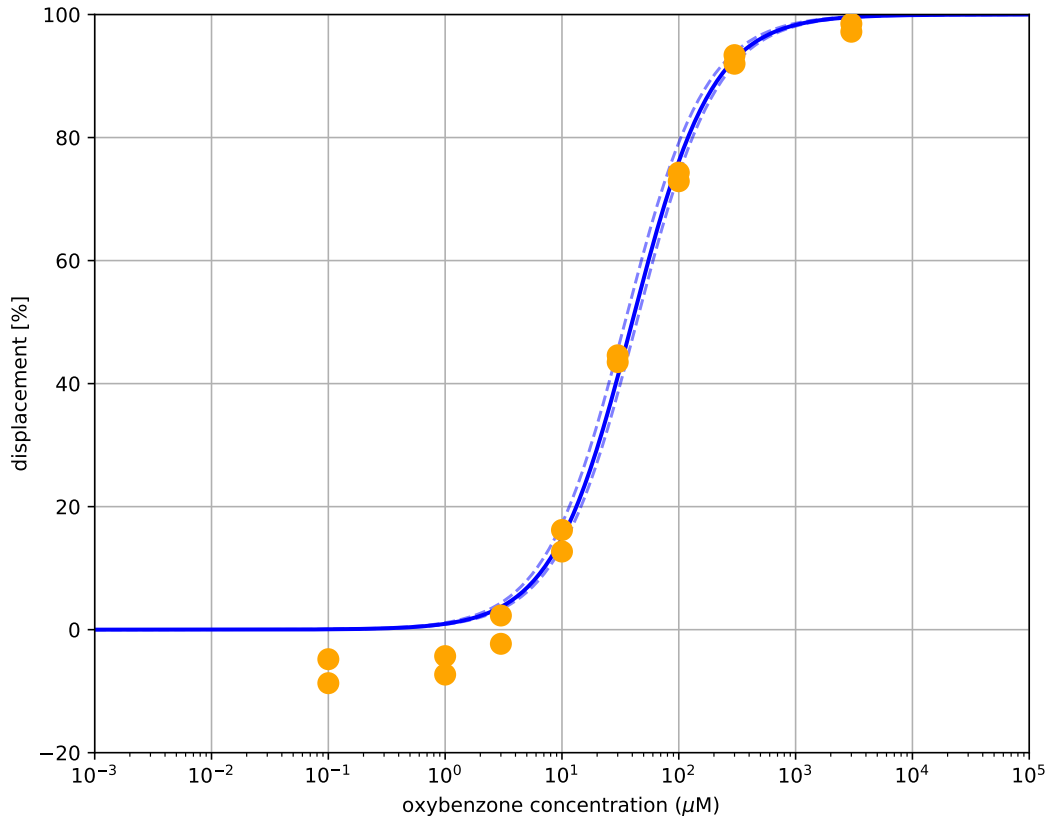


Oxybenzone-CAR, 95% C.I.(IC50) = [59.2µM, 84.1µM]


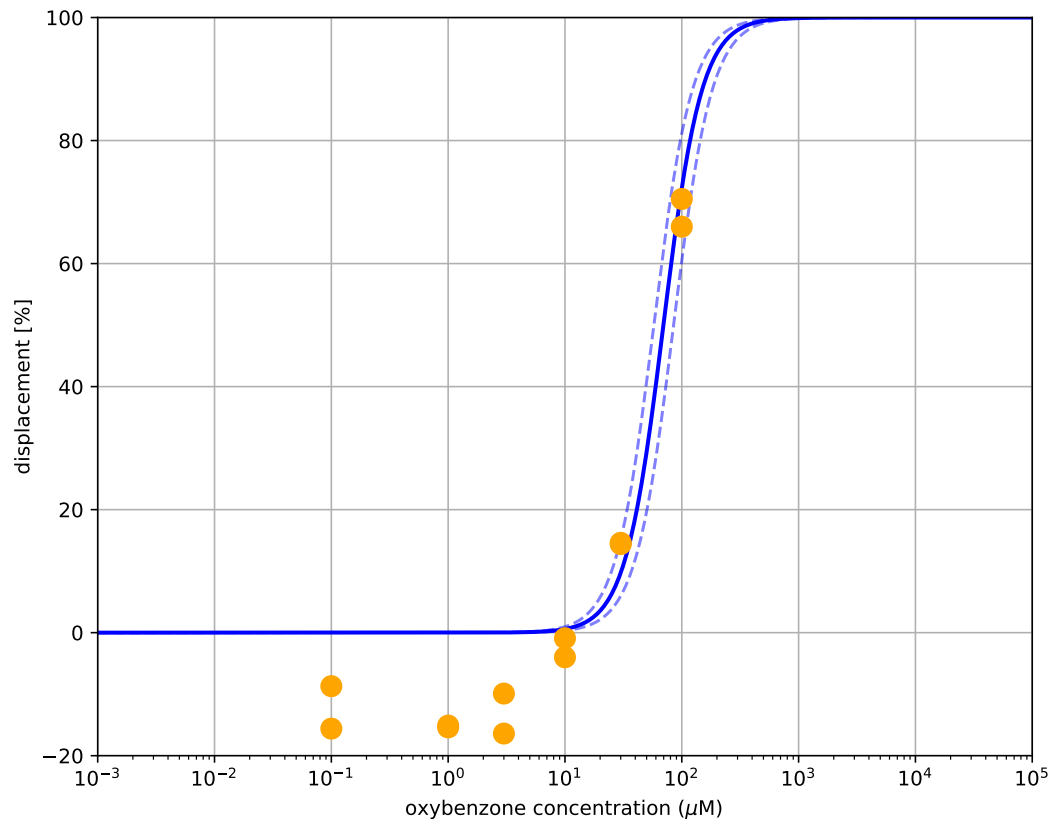


### Paraquat

Paraquat-ACHE, 95% C.I.(IC50) = [31.9µM, 54.2µM]


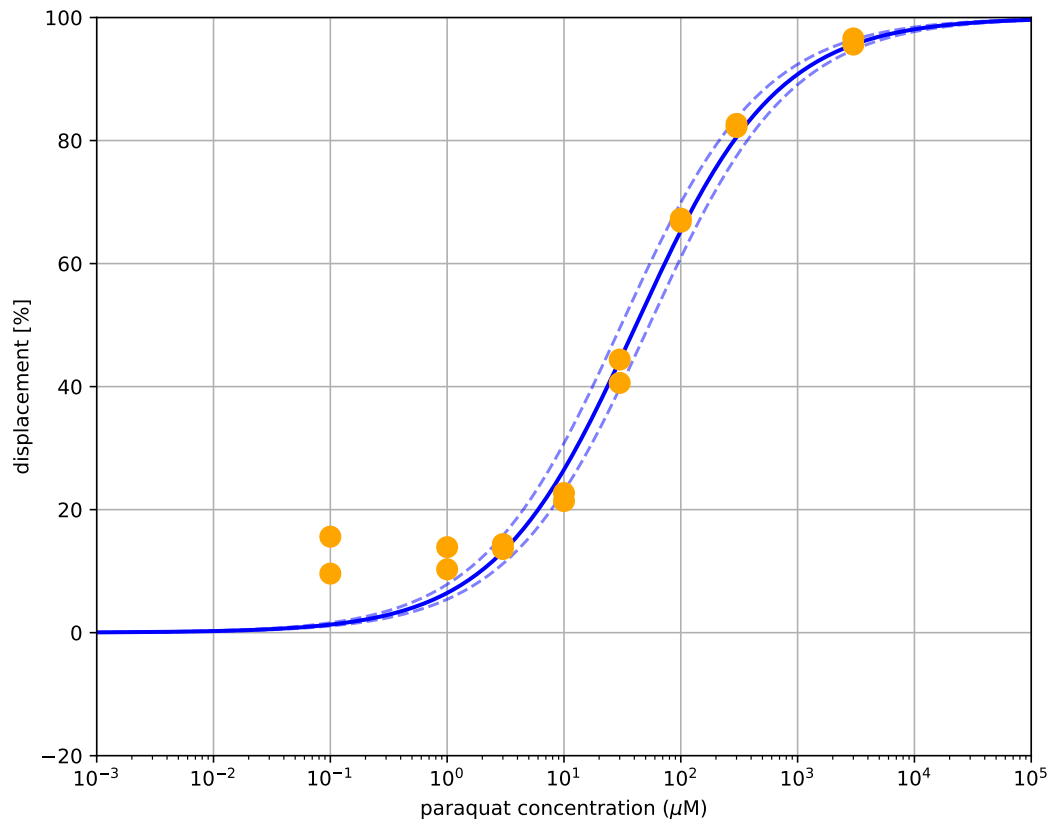


Paraquat-CHRM1, 95% C.I.(IC50) = [174.6µM, 231.2µM]


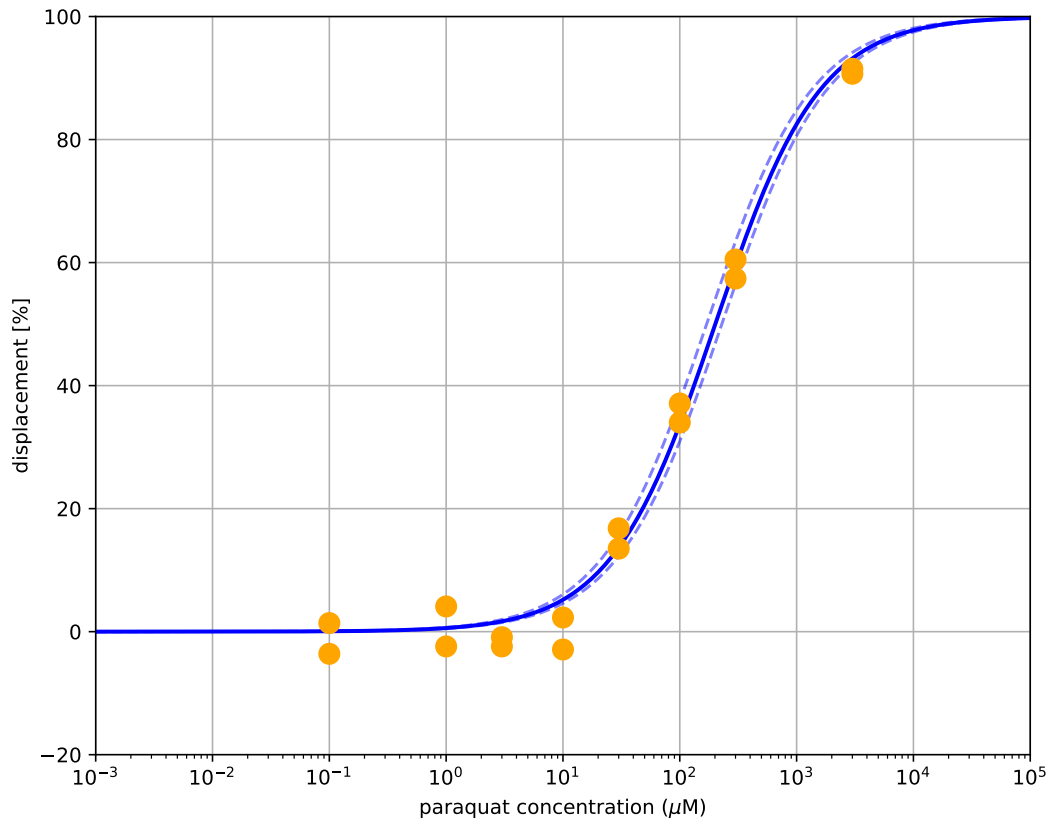


Paraquat-CHRM3, 95% C.I.(IC50) = [62.2µM, 90.1µM]


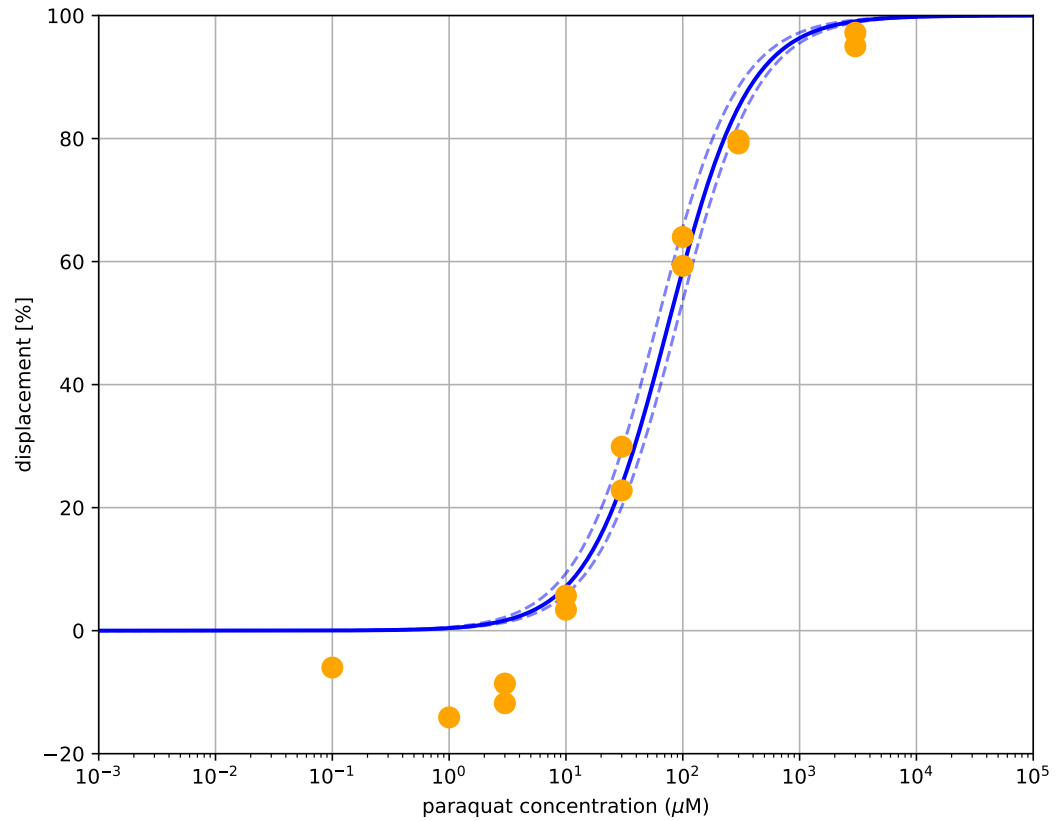


### Rosiglitazone

Rosiglitazone-MAOA, 95% C.I.(IC50) = [1.3µM, 2.0µM]


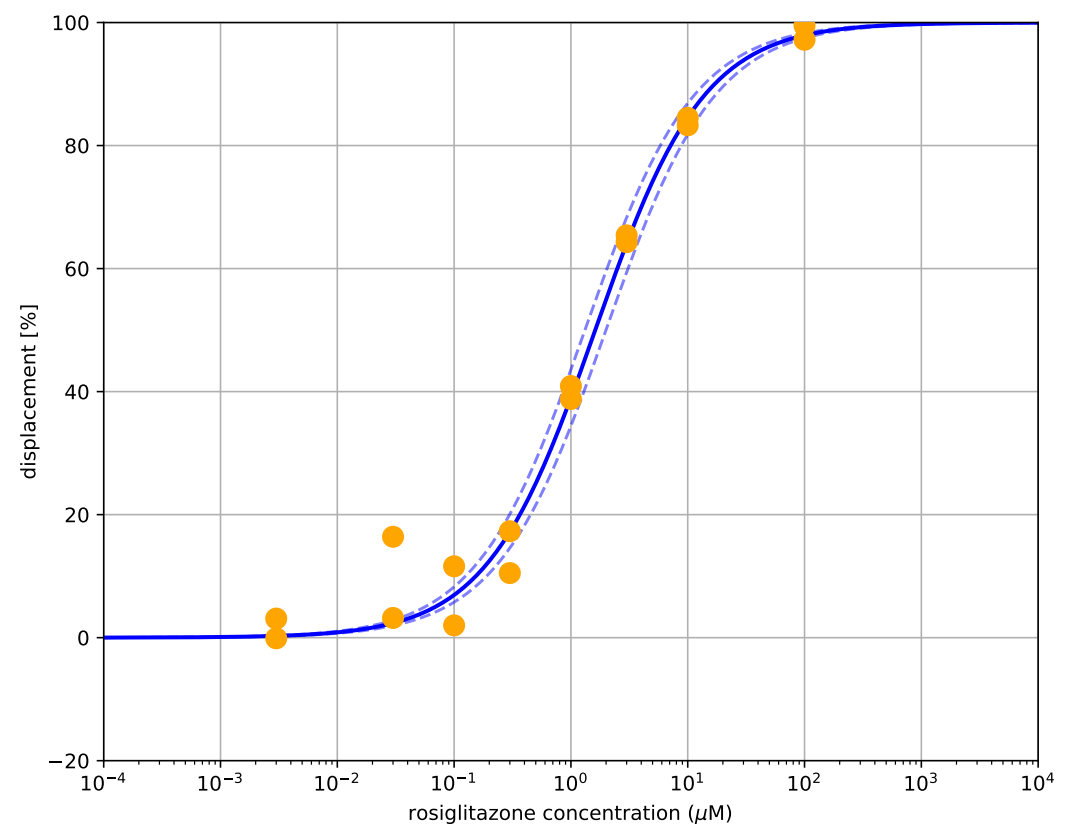


Rosiglitazone-PPARG, 95% C.I.(IC50) = [0.077µM, 0.11µM]


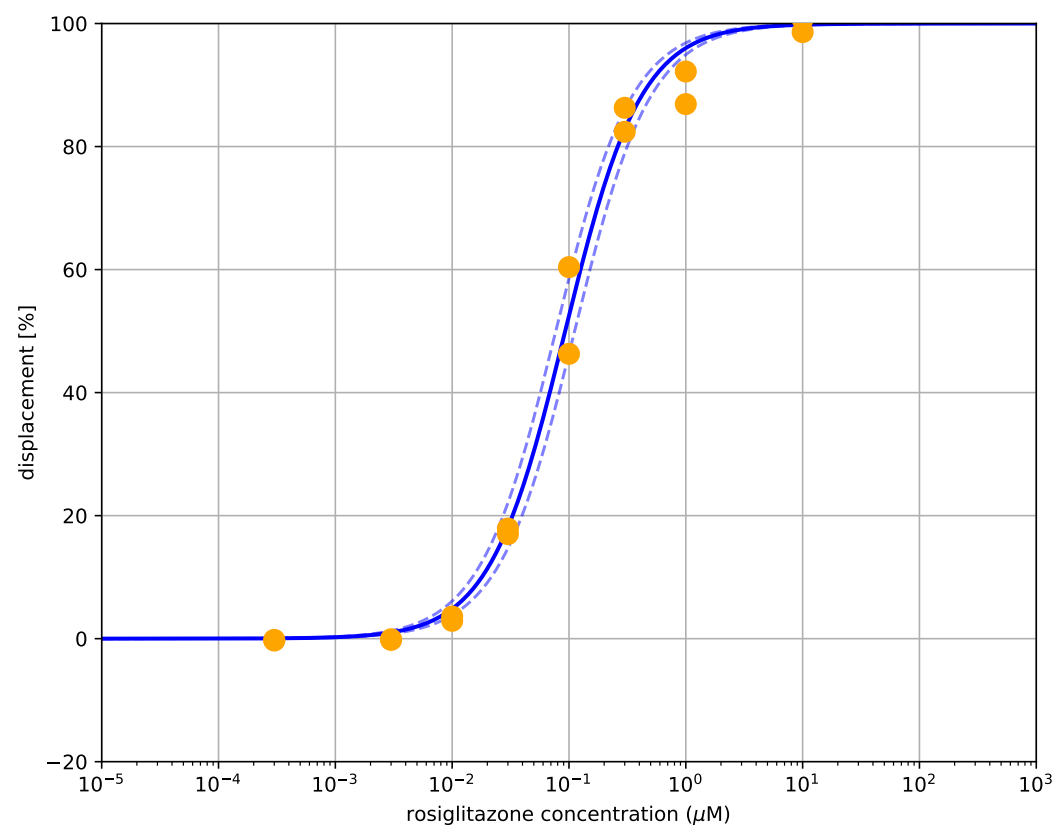


Rosiglitazone-CA2, 95% C.I.(IC50) = [3.9µM, 6.3µM]


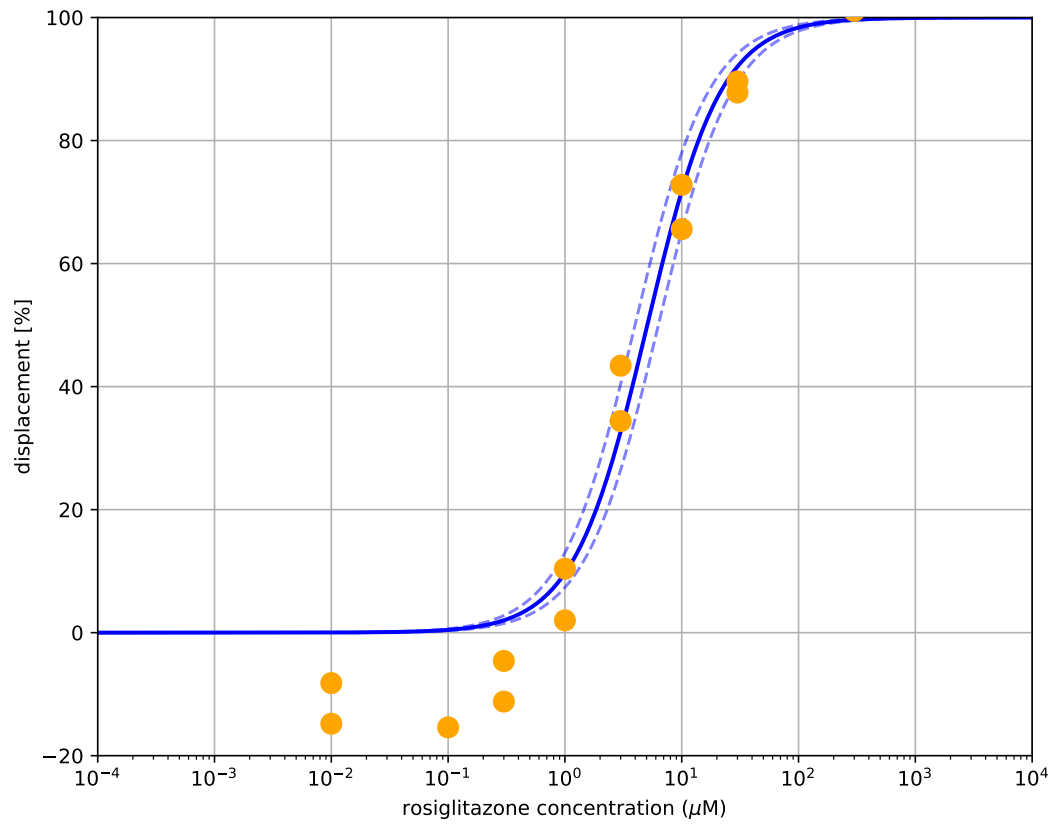


## F5. Cell Stress Panel Reproducibility

Comparison between the Hatherell et al (2020) global PODs and those obtained in this study for six chemicals (doxorubicin, rosiglitazone, coumarin, caffeine, niacinamide). The grey shaded region represents a difference of 1 order of magnitude above and below. All PoDs from this study were within 1 order of magnitude of the initial PoDs obtained from the previous experiment.


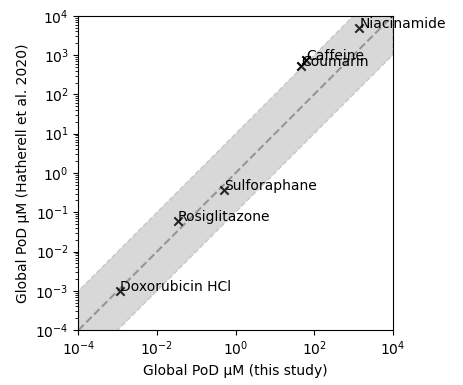


## F6. Utility and protectiveness of the toolbox for different confidence thresholds.

Empirical utility (blue) and protectiveness (orange) of the toolbox plotted against the confidence threshold for the three different PBK levels (L1-L3).


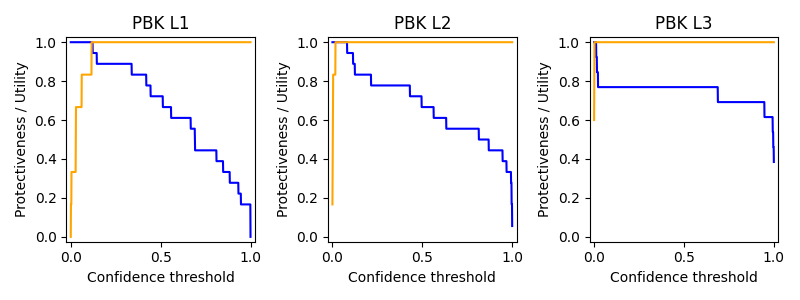


## F7. Toolbox bioactivity exposure ratio decision model.

The variance of the displayed probability distributions represents the uncertainty inherent to a BER, calculated at each PBK level. The requirement that 95% of the distribution of the BER being greater than one (red shaded region) translates into a higher threshold on the average BER (i.e. which is equal to the BER point-estimate, given by the PBK Cmax estimate and the minimum point of departure). This value called the ‘threshold BER’, denoted by the red vertical dashed lines. The condition that low-risk decisions should be maintained at subsequent PBK levels results in a higher confidence threshold (blue shaded region), which is equivalent to requiring higher threshold BER values (blue vertical dashed lines).


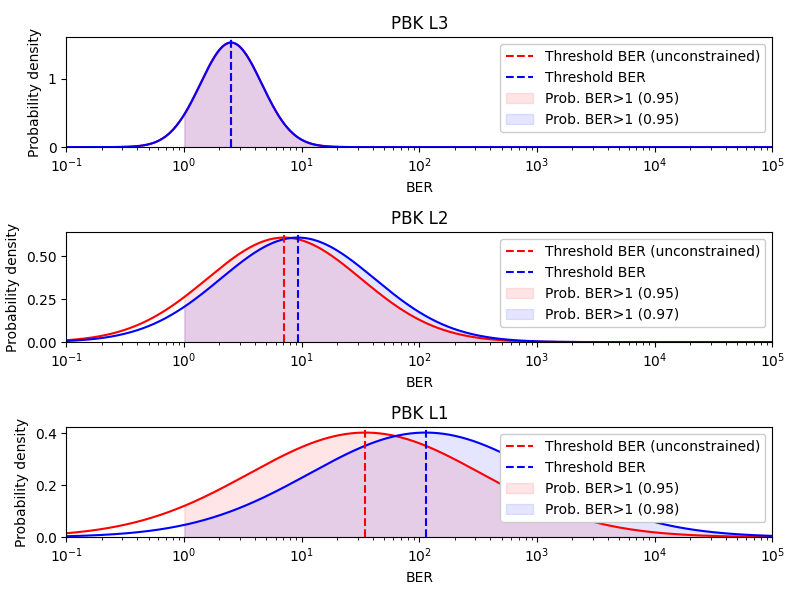


# References

Hatherell, S., M. T. Baltazar, J. Reynolds, P. L. Carmichael, M. Dent, H. Li, S. Ryder, A. White, P. Walker and A. M. Middleton (2020). "Identifying and characterizing stress pathways of concern for consumer safety in next-generation risk assessment." Toxicological Sciences **176**(1): 11-33.

Juárez, M. A. and M. F. J. Steel (2010). "Model-Based Clustering of Non-Gaussian Panel Data Based on Skew-t Distributions." Journal of Business & Economic Statistics **28**(1): 52-66.

Reynolds, J., S. Malcomber and A. White (2020). "A bayesian approach for inferring global points of departure from transcriptomics data." Computational Toxicology **16**: 100138.

Carpenter, B., Gelman, A., Hoffman, M. D., Lee, D., Goodrich, B., Betancourt, M., . . . Riddell, A. (2017). Stan: a probabilistic programming language. *Grantee Submission, 76*(1), 1-32.

Phillips, J. R., Svoboda, D. L., Tandon, A., Patel, S., Sedykh, A., Mav, D., . . . Thomas, R. S. (2019). BMDExpress 2: enhanced transcriptomic dose-response analysis workflow. *Bioinformatics, 35*(10), 1780-1782.

Reynolds, J., Malcomber, S., & White, A. (2020). A bayesian approach for inferring global points of departure from transcriptomics data. *Computational Toxicology, 16*, 100138.

Additives, E. P. o. F., & Food, N. S. a. t. (2012). Scientific Opinion on the re-evaluation of butylated hydroxytoluene BHT (E 321) as a food additive. *EFSA Journal*, *10*(3), 2588. <https://doi.org/https://doi.org/10.2903/j.efsa.2012.2588>

Argikar, U. A., & Remmel, R. P. (2009). Effect of Aging on Glucuronidation of Valproic Acid in Human Liver Microsomes and the Role of UDP-Glucuronosyltransferase UGT1A4, UGT1A8, and UGT1A10. *Drug Metabolism and Disposition*, *37*(1), 229-236. <https://doi.org/10.1124/dmd.108.022426>

Baltazar, M. T., Dinis-Oliveira, R. J., Bastos, M. d. L., Duarte, J. A., & Carvalho, F. (2014). Lysine acetylsalicylate improves the safety of paraquat formulation in rats by increasing its elimination and preventing lung and kidney injury. *Toxicology Research*, *3*(4), 266-277. <https://doi.org/10.1039/c3tx50102g>

Baltazar, M. T., Dinis-Oliveira, R. J., de Lourdes Bastos, M., Tsatsakis, A. M., Duarte, J. A., & Carvalho, F. (2014). Pesticides exposure as etiological factors of Parkinson's disease and other neurodegenerative diseases--a mechanistic approach. *Toxicol Lett*, *230*(2), 85-103. <https://doi.org/10.1016/j.toxlet.2014.01.039>

Bazargan, M., Foster, D. J. R., Davey, A. K., & Muhlhausler, B. S. (2017). Rosiglitazone Metabolism in Human Liver Microsomes Using a Substrate Depletion Method. *Drugs in R&amp;D*, *17*(1), 189-198. <https://doi.org/10.1007/s40268-016-0166-4>

Binda, C., Aldeco, M., Geldenhuys, W. J., Tortorici, M., Mattevi, A., & Edmondson, D. E. (2012). Molecular Insights into Human Monoamine Oxidase B Inhibition by the Glitazone Antidiabetes Drugs. *ACS Medicinal Chemistry Letters*, *3*(1), 39-42. <https://doi.org/10.1021/ml200196p>

Blanchard, J., & Sawers, S. J. (1983). The absolute bioavailability of caffeine in man. *Eur J Clin Pharmacol*, *24*(1), 93-98. <https://doi.org/10.1007/bf00613933>

Boo, Y. C. (2021). Mechanistic Basis and Clinical Evidence for the Applications of Nicotinamide (Niacinamide) to Control Skin Aging and Pigmentation. *Antioxidants*, *10*(8), 1315. <https://www.mdpi.com/2076-3921/10/8/1315>

Carpenter, B., Gelman, A., Hoffman, M. D., Lee, D., Goodrich, B., Betancourt, M., Brubaker, M., Guo, J., Li, P., & Riddell, A. (2017a). Stan: A probabilistic programming language. *Journal of statistical software*, *76*(1).

Carpenter, B., Gelman, A., Hoffman, M. D., Lee, D., Goodrich, B., Betancourt, M., Brubaker, M. A., Guo, J., Li, P., & Riddell, A. (2017b). Stan: a probabilistic programming language. *Grantee Submission*, *76*(1), 1-32.

Chapelsky, M. C., Thompson-Culkin, K., Miller, A. K., Sack, M., Blum, R., & Freed, M. I. (2003). Pharmacokinetics of rosiglitazone in patients with varying degrees of renal insufficiency. *J Clin Pharmacol*, *43*(3), 252-259. <https://doi.org/10.1177/0091270002250602>

Chassany, O., Urien, S., Claudepierre, P., Bastian, G., & Tillement, J.-P. (1996). Comparative serum protein binding of anthracycline derivatives [journal article]. *Cancer Chemotherapy and Pharmacology*, *38*(6), 571-573. <https://doi.org/10.1007/s002800050529>

ChemAxon., A. U.-M. J. (July 24, 2017). ACE and JChem acidity and basicity calculator. . <https://epoch.uky.edu/ace/public/pKa.jsp>

Chemicals., C. S. f. (Oct 26, 2016). *2-tert-Butylhydroquinone (1948-33-0) Registered Substances Dossier.* <http://echa.europa.eu/>

Chen, R., Wan, J., Song, J., Qian, Y., Liu, Y., & Gu, S. (2017). Rational screening of peroxisome proliferator-activated receptor-γ agonists from natural products: potential therapeutics for heart failure. *Pharmaceutical biology*, *55*(1), 503-509. <https://doi.org/10.1080/13880209.2016.1255648>

Cramer, J. A., & Mattson, R. H. (1979). Valproic acid: in vitro plasma protein binding and interaction with phenytoin. *Ther Drug Monit*, *1*(1), 105-116.

Davis, D. A., Kraus, A. L., Thompson, G. A., Olerich, M., & Odio, M. R. (1997). Percutaneous absorption of salicylic acid after repeated (14-day) in vivo administration to normal, acnegenic or aged human skin. *J Pharm Sci*, *86*(8), 896-899. <https://doi.org/10.1021/js960496b>

Del Pino, J., Moyano, P., Díaz, G. G., Anadon, M. J., Diaz, M. J., García, J. M., Lobo, M., Pelayo, A., Sola, E., & Frejo, M. T. (2017). Primary hippocampal neuronal cell death induction after acute and repeated paraquat exposures mediated by AChE variants alteration and cholinergic and glutamatergic transmission disruption. *Toxicology*, *390*, 88-99. <https://doi.org/10.1016/j.tox.2017.09.008>

Dinkova-Kostova, A. T., Fahey, J. W., Kostov, R. V., & Kensler, T. W. (2017). KEAP1 and Done? Targeting the NRF2 Pathway with Sulforaphane. *Trends in food science & technology*, *69*(Pt B), 257-269. <https://doi.org/10.1016/j.tifs.2017.02.002>

El-Demerdash, F. M., Yousef, M. I., & Elagamy, E. I. (2001). INFLUENCE OF PARAQUAT, GLYPHOSATE, AND CADMIUM ON THE ACTIVITY OF SOME SERUM ENZYMES AND PROTEIN ELECTROPHORETIC BEHAVIOR (IN VITRO). *Journal of Environmental Science and Health, Part B*, *36*(1), 29-42. <https://doi.org/10.1081/PFC-100000914>

EPA, U. (2018). *Estimation Program Interface (EPI) Suite.* Version 4.1). <https://www2.epa.gov/tsca-screening-tools>

European Commission, H. a. C. P. D.-G. (July 25, 2017). Opinion on Benzophenone-3. Report SCCP/1069/06 (Dec 2006). . <http://ec.europa.eu/health/ph_risk/committees/04_sccp/docs/sccp_o_078.pdf>

Feldmann, R. J., & Maibach, H. I. (1970). Absorption of some organic compounds through the skin in man. *J Invest Dermatol*, *54*(5), 399-404. <https://doi.org/10.1111/1523-1747.ep12259184>

Ford, R. A., Hawkins, D. R., Mayo, B. C., & Api, A. M. (2001). The in vivo dermal absorption and metabolism of [4-14C] coumarin by rats and by human volunteers under simulated conditions of use in fragrances. *Food Chem Toxicol*, *39*(2), 153-162. <https://doi.org/10.1016/s0278-6915(00)00123-x>

Gnerre, C., Catto, M., Leonetti, F., Weber, P., Carrupt, P.-A., Altomare, C., Carotti, A., & Testa, B. (2000). Inhibition of Monoamine Oxidases by Functionalized Coumarin Derivatives:  Biological Activities, QSARs, and 3D-QSARs. *Journal of Medicinal Chemistry*, *43*(25), 4747-4758. <https://doi.org/10.1021/jm001028o>

Hansch, C., Leo, A., & Hoekman, D. (Eds.). (1995). *Exploring QSAR: Hydrophobic, electronic, and steric constants* (Vol. 2). American Chemical Society.

Hansch, C., Leo, A., & Hoekman, D. H. (1995). *Exploring QSAR : hydrophobic, electronic, and steric constants*. Washington. 348 p.

Hatherell, S., Baltazar, M. T., Reynolds, J., Carmichael, P. L., Dent, M., Li, H., Ryder, S., White, A., Walker, P., & Middleton, A. M. (2020). Identifying and Characterizing Stress Pathways of Concern for Consumer Safety in Next-Generation Risk Assessment. *Toxicological Sciences*, *176*(1), 11-33. <https://doi.org/10.1093/toxsci/kfaa054>

Hayden, C., Cross, S., Anderson, C., Saunders, N., & Roberts, M. (2005). Sunscreen Penetration of Human Skin and Related Keratinocyte Toxicity after Topical Application. *Skin pharmacology and physiology*, *18*, 170-174. <https://doi.org/10.1159/000085861>

Hu, D., Wu, C. Q., Li, Z. J., Liu, Y., Fan, X., Wang, Q. J., & Ding, R. G. (2015). Characterizing the mechanism of thiazolidinedione-induced hepatotoxicity: An in vitro model in mitochondria. *Toxicol Appl Pharmacol*, *284*(2), 134-141. <https://doi.org/10.1016/j.taap.2015.02.018>

Huang, C. L., Chao, C. C., Lee, Y. C., Lu, M. K., Cheng, J. J., Yang, Y. C., Wang, V. C., Chang, W. C., & Huang, N. K. (2016). Paraquat Induces Cell Death Through Impairing Mitochondrial Membrane Permeability. *Mol Neurobiol*, *53*(4), 2169-2188. <https://doi.org/10.1007/s12035-015-9198-y>

Jacobson, K. A., Gao, Z.-G., Matricon, P., Eddy, M. T., & Carlsson, J. Adenosine A2A receptor antagonists: from caffeine to selective non-xanthines. *British Journal of Pharmacology*, *n/a*(n/a). <https://doi.org/https://doi.org/10.1111/bph.15103>

Juárez, M. A., & Steel, M. F. J. (2010). Model-Based Clustering of Non-Gaussian Panel Data Based on Skew-t Distributions. *Journal of Business & Economic Statistics*, *28*(1), 52-66. <https://doi.org/10.1198/jbes.2009.07145>

Kochak, G. M., Sun, J. X., Choi, R. L., & Piraino, A. J. (1992). Pharmacokinetic disposition of multiple-dose transdermal nicotine in healthy adult smokers. *Pharmaceutical Research*, *9*(11), 1451-1455.

Kubitschke, J. (2014). Carboxylic acids, aliphatic. In *Ullmann's Encyclopedia of Industrial Chemistry*. John Wiley & Sons.

Lake, B. G., & Grasso, P. (1996). Comparison of the hepatotoxicity of coumarin in the rat, mouse, and Syrian hamster: a dose and time response study. *Fundam Appl Toxicol*, *34*(1), 105-117. <https://doi.org/10.1006/faat.1996.0181>

Lecka-Czernik, B., Ackert-Bicknell, C., Adamo, M. L., Marmolejos, V., Churchill, G. A., Shockley, K. R., Reid, I. R., Grey, A., & Rosen, C. J. (2007). Activation of Peroxisome Proliferator-Activated Receptor γ (PPARγ) by Rosiglitazone Suppresses Components of the Insulin-Like Growth Factor Regulatory System in Vitro and in Vivo. *Endocrinology*, *148*(2), 903-911. <https://doi.org/10.1210/en.2006-1121>

Lin, Z. J., Desai-Krieger, D., & Shum, L. (2004). Simultaneous determination of glipizide and rosiglitazone unbound drug concentrations in plasma by equilibrium dialysis and liquid chromatography–tandem mass spectrometry. *Journal of Chromatography B*, *801*(2), 265-272. <https://doi.org/https://doi.org/10.1016/j.jchromb.2003.11.019>

Lu, A. T., Frisella, M. E., & Johnson, K. C. (1993). Dissolution modeling: factors affecting the dissolution rates of polydisperse powders. *Pharm Res*, *10*(9), 1308-1314. <https://doi.org/10.1023/a:1018917729477>

MacBean, C., & Council, B. C. P. (2012). *The Pesticide Manual: A World Compendium*. British Crop Protection Council. <https://books.google.co.uk/books?id=EVuZMgEACAAJ>

Maresca, A., & Supuran, C. T. (2010). Coumarins incorporating hydroxy- and chloro-moieties selectively inhibit the transmembrane, tumor-associated carbonic anhydrase isoforms IX and XII over the cytosolic ones I and II. *Bioorganic & Medicinal Chemistry Letters*, *20*(15), 4511-4514. <https://doi.org/https://doi.org/10.1016/j.bmcl.2010.06.040>

Maresca, A., Temperini, C., Vu, H., Pham, N. B., Poulsen, S.-A., Scozzafava, A., Quinn, R. J., & Supuran, C. T. (2009). Non-Zinc Mediated Inhibition of Carbonic Anhydrases: Coumarins Are a New Class of Suicide Inhibitors. *Journal of the American Chemical Society*, *131*(8), 3057-3062. <https://doi.org/10.1021/ja809683v>

Matta, M. K., Florian, J., Zusterzeel, R., Pilli, N. R., Patel, V., Volpe, D. A., Yang, Y., Oh, L., Bashaw, E., Zineh, I., Sanabria, C., Kemp, S., Godfrey, A., Adah, S., Coelho, S., Wang, J., Furlong, L. A., Ganley, C., Michele, T., & Strauss, D. G. (2020). Effect of Sunscreen Application on Plasma Concentration of Sunscreen Active Ingredients: A Randomized Clinical Trial. *Jama*, *323*(3), 256-267. <https://doi.org/10.1001/jama.2019.20747>

Moxon, T. E., Li, H., Lee, M. Y., Piechota, P., Nicol, B., Pickles, J., Pendlington, R., Sorrell, I., & Baltazar, M. T. (2020). Application of physiologically based kinetic (PBK) modelling in the next generation risk assessment of dermally applied consumer products. *Toxicol In Vitro*, *63*, 104746. <https://doi.org/10.1016/j.tiv.2019.104746>

Mueller, S. L., Chrysanthopoulos, P. K., Halili, M. A., Hepburn, C., Nebl, T., Supuran, C. T., Nocentini, A., Peat, T. S., & Poulsen, S.-A. (2021). The Glitazone Class of Drugs as Carbonic Anhydrase Inhibitors-A Spin-Off Discovery from Fragment Screening. *Molecules (Basel, Switzerland)*, *26*(10), 3010. <https://doi.org/10.3390/molecules26103010>

Nieva-Echevarría, B., Manzanos, M. J., Goicoechea, E., & Guillén, M. D. (2015). 2,6-Di-Tert-Butyl-Hydroxytoluene and Its Metabolites in Foods. *Comprehensive Reviews in Food Science and Food Safety*, *14*(1), 67-80. <https://doi.org/https://doi.org/10.1111/1541-4337.12121>

Otberg, N., Patzelt, A., Rasulev, U., Hagemeister, T., Linscheid, M., Sinkgraven, R., Sterry, W., & Lademann, J. (2008). The role of hair follicles in the percutaneous absorption of caffeine. *British journal of clinical pharmacology*, *65*(4), 488-492. <https://doi.org/10.1111/j.1365-2125.2007.03065.x>

Perucca, E., Gatti, G., Frigo, G. M., & Crema, A. (1978). Pharmacokinetics of valproic acid after oral and intravenous administration. *British journal of clinical pharmacology*, *5*(4), 313-318. <http://europepmc.org/abstract/PMC/PMC1429296>

<https://www.ncbi.nlm.nih.gov/pmc/articles/PMC1429296/?tool=EBI>

<https://www.ncbi.nlm.nih.gov/pmc/articles/PMC1429296/pdf/?tool=EBI>

<https://europepmc.org/articles/PMC1429296>

<https://europepmc.org/articles/PMC1429296?pdf=render>

Petzer, A., Pienaar, A., & Petzer, J. P. (2013). The interactions of caffeine with monoamine oxidase. *Life Sci*, *93*(7), 283-287. <https://doi.org/10.1016/j.lfs.2013.06.020>

Phillips, J. R., Svoboda, D. L., Tandon, A., Patel, S., Sedykh, A., Mav, D., Kuo, B., Yauk, C. L., Yang, L., & Thomas, R. S. (2019). BMDExpress 2: enhanced transcriptomic dose-response analysis workflow. *Bioinformatics*, *35*(10), 1780-1782.

Platford, R. F. (1983). The octanol-water partitioning of some hydrophobic and hydrophilic compounds. *Chemosphere*, *12*(7), 1107-1111. <https://doi.org/https://doi.org/10.1016/0045-6535(83)90264-3>

Pohanka, M. (2015). The perspective of caffeine and caffeine derived compounds in therapy. *Bratislavske lekarske listy*, *116*(9), 520-530. <https://doi.org/10.4149/bll_2015_106>

Real, A. M., Hong, S., & Pissios, P. (2013). Nicotinamide N-oxidation by CYP2E1 in human liver microsomes. *Drug metabolism and disposition: the biological fate of chemicals*, *41*(3), 550-553. <https://doi.org/10.1124/dmd.112.049734>

Reynolds, J., Malcomber, S., & White, A. (2020). A bayesian approach for inferring global points of departure from transcriptomics data. *Computational Toxicology*, *16*, 100138.

Ritschel, W. A., & Grummich, K. W. (1981). Pharmacokinetics of coumarin and 7-hydroxycoumarin upon i.v. and p.o. administration in the euthyroid and hypothyroid beagle dog. *Arzneimittel-Forschung*, *31 4*, 643-649.

Ryu, R. J., Eyal, S., Kaplan, H. G., Akbarzadeh, A., Hays, K., Puhl, K., Easterling, T. R., Berg, S. L., Scorsone, K. A., Feldman, E. M., Umans, J. G., Miodovnik, M., & Hebert, M. F. (2014). Pharmacokinetics of doxorubicin in pregnant women [journal article]. *Cancer Chemotherapy and Pharmacology*, *73*(4), 789-797. <https://doi.org/10.1007/s00280-014-2406-z>

Sangster, J. M. (1997). LOGKOW A Databank of Evaluated Octanol-Water Partition Coefficients.

SCCS. (2021). SCCS (Scientific Committee on Consumer Safety), Opinion on Benzophenone-3 (CAS No 131-57-7, EC No 205-031-5), preliminary version of 15 December 2020, final version of 30-31 March 2021, SCCS/1625/20.

Seth, B. L. (1992). Comparative pharmacokinetics and bioavailability study of percutaneous absorption of diclofenac from two topical formulations containing drug as a solution gel or as an emulsion gel. *Arzneimittelforschung*, *42*(2), 120-122.

Shibata, Y., Takahashi, H., Chiba, M., & Ishii, Y. (2002). Prediction of Hepatic Clearance and Availability by Cryopreserved Human Hepatocytes: An Application of Serum Incubation Method. *Drug metabolism and disposition: the biological fate of chemicals*, *30*, 892-896. <https://doi.org/10.1124/dmd.30.8.892>

Sipes, N. S., Martin, M. T., Kothiya, P., Reif, D. M., Judson, R. S., Richard, A. M., Houck, K. A., Dix, D. J., Kavlock, R. J., & Knudsen, T. B. (2013). Profiling 976 ToxCast chemicals across 331 enzymatic and receptor signaling assays. *Chemical research in toxicology*, *26*(6), 878-895. <https://doi.org/10.1021/tx400021f>

Sivamani, R., Jagdeo, J.R., Elsner, P., & Maibach, H.I. . (2015). *Cosmeceuticals and Active Cosmetics*. CRC PRESS.

Skorokhod, O. A., Kulikova, E. V., Galkina, N. M., Medvedev, P. V., Zybunova, E. E., Vitvitsky, V. M., Pivnik, A. V., & Ataullakhanov, F. I. (2007). Doxorubicin pharmacokinetics in lymphoma patients treated with doxorubicin-loaded eythrocytes. *Haematologica*, *92*(4), 570-571. <https://doi.org/10.3324/haematol.10770>

SPARC. (2008). *pKa/property server.*

Thorn, C. F., Oshiro, C., Marsh, S., Hernandez-Boussard, T., McLeod, H., Klein, T. E., & Altman, R. B. (2011). Doxorubicin pathways: pharmacodynamics and adverse effects. *Pharmacogenetics and genomics*, *21*(7), 440-446. <https://doi.org/10.1097/FPC.0b013e32833ffb56>

Torii, M., Takiguchi, Y., Izumi, M., Fukushima, T., & Yokota, M. (2002). Carbapenem antibiotics inhibit valproic acid transport in Caco-2 cell monolayers. *International Journal of Pharmaceutics*, *233*(1), 253-256. <https://doi.org/https://doi.org/10.1016/S0378-5173(01)00916-4>

Verhagen, H., Beckers, H. H. G., Comuth, P. A. W. V., Maas, L. M., ten Hoor, F., Henderson, P. T., & Kleinjans, J. C. S. (1989). Disposition of single oral doses of butylated hydroxytoluene in man and rat. *Food and Chemical Toxicology*, *27*(12), 765-772. <https://doi.org/https://doi.org/10.1016/0278-6915(89)90105-1>

Williams, M. (2013). The Merck Index: An Encyclopedia of Chemicals, Drugs, and Biologicals, 15th Edition Edited by M.J. O'Neil , Royal Society of Chemistry, Cambridge, UK ISBN 9781849736701; 2708 pages. April 2013, $150 with 1-year free access to The Merck Index Online. *Drug Development Research*, *74*(5), 339-339. <https://doi.org/https://doi.org/10.1002/ddr.21085>

Yalkowsky, S. H., He, Yan., . . (2003). Handbook of Aqueous Solubility Data: An Extensive Compilation of Aqueous Solubility Data for Organic Compounds Extracted from the AQUASOL dATAbASE. *CRC Press LLC*.

Yoshida, H., Goto, M., Honda, A., Nabeshima, T., Kumazawa, T., Inagaki, J., Yamanaka, N., & Ota, K. (1994). Pharmacokinetics of doxorubicin and its active metabolite in patients with normal renal function and in patients on hemodialysis. *Cancer Chemother Pharmacol*, *33*(6), 450-454.

1. The simplest of Bayesian model may be univariate. [↑](#footnote-ref-2)
